# Pyruvate Dehydrogenase Kinase 1 inhibition mediated oxidative phosphorylation enhancement in cartilage promotes osteoarthritis progression

**DOI:** 10.1186/s12891-023-06585-6

**Published:** 2023-07-20

**Authors:** Xian Yang, Qingsong Jiang, Tiankuo Luan, Chao Yu, Zhibo Liu, Ting Wang, Jingyuan Wan, Jiayu Huang, Ke Li

**Affiliations:** 1 Department of Pharmacology, Chongqing Medical University, Chongqing, China; 2 Department of Human Anatomy, Basic Medical School, Chongqing Medical University, Chongqing, China; 3 Department of Orthopedic Surgery, University-Town Hospital of Chongqing Medical University, Chongqing, China; 4 Department of Orthopedic Surgery, The Second Affiliated Hospital of Chongqing, Chongqing, China; 5 Reproductive Medicine Center, The First Affiliated Hospital of Chongqing, Chongqing, China; 6 Department of Orthopedics Surgery, the First Affiliated Hospital of Chongqing Medical University, Chongqing, China; 7 Orthopedic Laboratory of Chongqing Medical University, Chongqing, China

**Keywords:** Osteoarthritis, PDK1, ECM, Glucose metabolism, Inflammation

## Abstract

Osteoarthritis (OA) is a common disease characterized by cartilage degradation. Growing evidence showed that glucose metabolism impacts joint homeostasis and an imbalance between glycolysis and oxidative phosphorylation (OXPHOS) may exacerbate OA progression, however, a definitive link is yet to be established. Here, we report that pyruvate metabolism and oxidative phosphorylation pathway is enriched in OA cartilage through gene set enrichment analysis (GSEA) and expression of Pyruvate Dehydrogenase Kinase 1 (PDK1), an enzyme that can phosphorylate Pyruvate Dehydrogenase (PDH), and inhibit pyruvate fluxes into the tricarboxylic acid (TCA) cycle and to OXPHOS, in articular cartilage is notably reduced through destabilization of medial meniscus (DMM). Moreover, by inhibiting PDK1, cartilage loss is markedly accelerated in DMM-induced OA through extracellular matrix (ECM) degradation and apoptosis of chondrocytes. These results indicate that PDK1 is involved in the progression of OA through accelerating cartilage matrix degradation and synovium inflammation to ameliorate cartilage degeneration.

**Supplementary Information:**

The online version contains supplementary material available at 10.1186/s12891-023-06585-6.

## Introduction

Osteoarthritis (OA) is characterized by cartilage erosion, osteophyte formation, subchondral bone remodeling, and synovial hyperplasia [1]. There is currently no viable treatment that can effectively prevent or reverse progressive osteoarthritis in clinical treatment owing to the lack of understanding of disease pathogenesis [2]. The progressive destruction of the extracellular matrix (ECM) is considered as its hallmark [3, 4]. In osteoarthritic cartilage ECM, pathological changes, including the degeneration of the functional matrix and the production of fibrous ECM, are caused by an imbalance of the anabolic and catabolic chondrocytes [5, 6]. Therefore, maintaining the homeostatic balance of anabolic and catabolic is essential for regulating the process of OA.

Evidence is mounting that OA is a degenerative disease and a metabolic disorder [7]. Recently, growing evidence showed that glucose metabolism impacts joint homeostasis and an imbalance between glycolysis and OXPHOS may exacerbate OA progression [8–10]. Knocking out Glucose Transporter Type 1 (Glut1) in chondrocytes could accelerate the process of OA [11]. In addition, recent studies have indicated that chondrocytes in OA cartilage undergo glucose metabolic changes, although evidence of these changes in osteoarthritis pathology remains limited [7]., In healthy cartilage, chondrocytes are highly glycolytic cells, [7]. However, in a pathological state, the incidence of anaerobic glycolysis in chondrocytes in OA is reduced [12, 13]. Pyruvate Dehydrogenase Kinase 1 (PDK1) can phosphorylate Pyruvate Dehydrogenase (PDH), and inhibit pyruvate fluxes into the tricarboxylic acid (TCA) cycle, causing a metabolic shift to glycolysis [14]. PDK1 affects matrix metalloproteinase expression in cancer cells [15]. PDK1 can affect osteoblast differentiation and bone formation [16]. Moreover, PDKs are associated with inflammatory metabolic disorders [17]. However, the role of PDK1 in chondrocyte glucose metabolism and OA progression is yet to be established. Here, we show that PDK1 is notably reduced in OA progression. Moreover, by inhibiting PDK1, cartilage loss is markedly accelerated in DMM-induced OA through ECM degradation and apoptosis of chondrocytes. These results, therefore, indicate a critical role for PDK1-mediated balance between glycolysis and OXPHOS in cartilage homeostasis.

## Materials and methods

### Chemicals and reagents

Modified Safranine O-Fast Green FCF Cartilage Stain Kit (G1371, Solarbio) was purchased from Solarbio Life Science (Beijing, China). Jx06 was obtained from MedChemExpress. Hematoxylin and Eosin were supplied by AMRESCO.

### Microarray data acquisition

Microarray data for OA and normal knee cartilage from the GEO database. Screening criteria included the following: (1) Human normal and OA knee cartilage isolated patients undergoing knee replacement surgery; (2) dataset containing complete information about samples. Finally, GSE55457 (includes 10 normal samples and 10 OA samples), GSE55235 (includes 10 normal samples and 10 OA samples), and GSE98918 (includes 12 normal samples and 12 OA samples) were downloaded.

### Enrichment analysis

GSEA is used to assess gene distribution trends of predefined sets in gene tables to determine their contribution to phenotypes [18]. We downloaded GSEA_4.2.3, c2.cp.kegg. v7.5.1.symbols.gmt, Dc5.go.bp.v7.5.1.symbols.gmt and c5.go.mf. v7.5.1.symbols.gmt for functional enrichment analyses. An ordered list of genes based on the correlation between all genes and OA was generated using GSEA.

### PPI (Protein–protein interaction) Network Construction and Functional analysis of GO and KEGG

The GeneMANIA (http://www.genemania.org) tool was applied to input PDK1 to obtain genes with high correlation with PDK1, and PPI network was constructed based on these genes [19]. We performed gene ontology (GO) and Kyoto Encyclopedia of Genes and Genomes (KEGG) pathways on PDK1-related gene using the R package "clusterprofiler" [20].

### Animals

Eight-week-old C57BL/6 male mice weighing 20 ~ 25 g were provided by the Laboratory Animal Center of Chongqing Medical University (Chongqing, China). Experimental animals were housed in a standard laboratory at controlled temperature (20–25℃), humidity (55 ± 5%), and 12 h light–dark cycle and given free access to food and water. All the experiments involving mice were guided by the Institutional Animal Care and Use Committee of Chongqing Medical University. Mice were fasted for 12 h but had free access to water before surgery. OA was induced by destabilization of the medial meniscus (DMM) surgery in 8-week-old C57BL/6 mice. Mice were also induced by intraarticular injection (once weekly for 3 weeks) of 10 ul of jx06 (30 mg/ml). 24 mice were used in this study. All mice were divided into the Sham group, Sham + jx06 group, DMM 4 w group, DMM 4 w + Jx06 group, DMM 8 w group, and DMM 8w + jx06 group, with 4 mice per group.

### Human samples

Human cartilage was obtained from patients undergoing total knee replacement surgery at the First Affiliated Hospital of Chongqing Medical University (Chongqing, China). The study was approved by the Ethics Committee of the First Affiliated Hospital of Chongqing Medical University.

### Histological and immunohistochemistry

All knee joints were fixed with 4% paraformaldehyde (PFA) for 3 days, decalcified in EDTA for 2 weeks, embedded in paraffin, and sectioned at a thickness of 6 um. Sections were stained with safranin-O/Fast Green (Saf-O). Medial tibial plateau and medial femoral condyle destruction were scored by two independent observers using the Osteoarthritis Research Society International (OARSI) scoring system [21]. Synovitis was determined by hematoxylin (H&E) staining, and synovial inflammation was scored as described previously [22]. Immunohistochemical staining was performed on paraffin sections. The sections were stained with a primary antibody against matrix metalloproteinase (A00420-2, Boster), type II collagen (BS-10589R, Bioss), aggrecan (BA2967-1, Boster), F4/80 (14,480,181, eBioscience) and TNF-a (AMC3012, Thermo) 14 h at 4℃, followed by subsequent experiments according to the ABC-HRP kit instruction (PK4001, Vector).

### Immunofluorescence staining

All knee joints were fixed with 4% PFA for 14 h at 4 ℃ and decalcified in EDTA for 3 days. Next, the joints were infiltrated with 15% and 30% sucrose for 24 h at 4 °C, embedded in optimal cutting temperature (Tissue-Tek), sectioned at a thickness of 8 um, and then maintained at − 20 °C. The sections were stained with a primary antibody against PDK1 (A01268-1, Boster), followed by the secondary antibody and Dapi.

### Tunel assays

Tunel (terminal deoxynucleotidyl transferase dUTP nick end labeling) assays were performed on paraffin sections using the In Site Cell Death Detection Kit, POD (Roche, 11,684,817,910). After antigen retrieval, the sections are permeabilized with 0.1% Triton X-100, then incubated with the reaction mixture for 1 h at 37 °C, incubated with POD for another 30 min, and finally chromogenic with DAB.

### Measurements of PDH activity

PDH activity was determined by using the Pyruvate Dehydrogenase Assay Kit (Bioss, AK290). Briefly, Articular cartilage samples are homogenized using a tissue homogenizer, and precipitation was gained by centrifugation, and the activity of PDH in cartilage was measured according to the manufacturer’s instruction.

### Statistical analysis

All experiments in this study were displayed as mean ± standard deviation (SD). Statistical analysis was performed by using Graphpad Prism software version 9.0.0. Between the two groups, a t-test was used to compare the difference. One-way ANOVA was used for multiple treatments, and followed by Tukey’s Honest Significant Difference post-hoc tests.

## Results

### Identification of OA-related gene signatures using gene set enrichment analysis (GSEA)

**Fig. 1:**
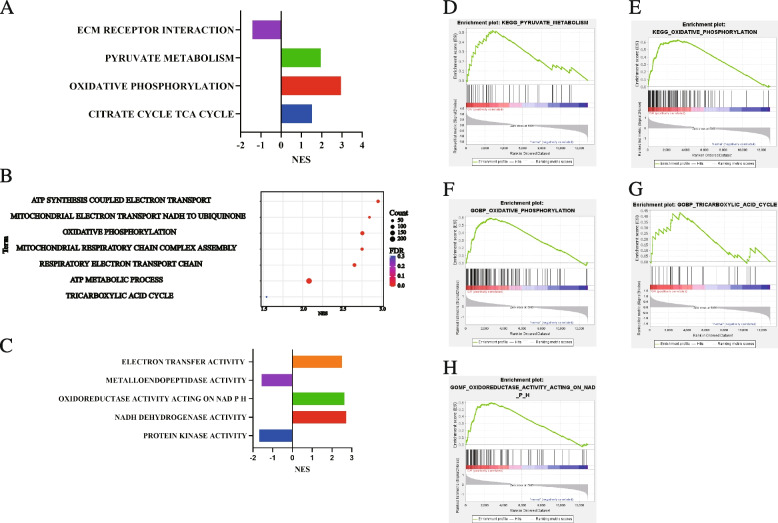
Enrichment plots from gene set enrichment analysis (GSEA). **A** Enrichment plots of metabolism-relevant enrichment pathway in c2.cp.kegg.v2022.1.Hs.symbols.gmt from GSEA **B** Enrichment plots of metabolism-relevant enrichment pathway in c5.go.bp.v2022.1.Hs.symbols.gmt from GSEA. **C** Enrichment plots of metabolism-relevant enrichment pathway in c5.mf.bp.v2022.1.Hs.symbols.gmt from GSEA. **D** Enrichment plot: Kegg Pyruvate Metabolism. **E** Enrichment plot: Kegg Oxidative Phosphorylation. **F** Enrichment plot: GoBP Oxidative Phosphorylation (**G**) Enrichment plot: GoBP Tricarboxylic Acid Cycle. **H** Enrichment plot: GoMF: oxidoreductase activity acting on NADPH. (*FDR* < *0.5, P* < *0.05*)

To gain insight into the effect between metabolism and osteoarthritis, GSEA was used to analysis enriched GO and KEGG pathway in metabolism-related signaling pathways in GSE55457. KEGG enrichment analysis shown that Citrate cycle TCA cycle, pyruvate metabolism, and oxidative phosphorylation gene sets most associated with OA. ECM receptor interaction gene set was enriched in normal (Fig. 1A, D, and E). GO Biological Process (BP) enrichment analysis indicated that ATP synthesis coupled electron transport, mitochondrial electron transport NADH to ubiquinone, oxidative phosphorylation, mitochondrial respiratory chain complex assembly, respiratory electron transport chain, ATP metabolic process, and tricarboxylic acid cycle gene sets were enriched in OA (Fig. 1B, F and G). GO Molecular Function (MF) enrichment analysis meant that electron transfer activity, oxidoreductase activity acting on NADPH, and NADH dehydrogenase activity gene sets were enriched in OA. Metalloendopeptidase activity and protein kinase activity gene sets were enriched in normal. (Fig. 1C and H). Detailed enrichment analysis information is shown in Supplementary table 1.

### PDK1 is involved in OA progression based on GEO

**Fig. 2:**
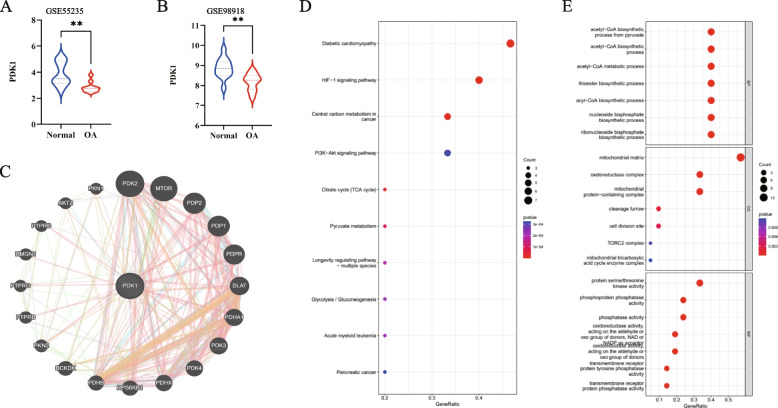
PDK1 is involved in OA progression based on GEO. **A** Differential expression of PDK1 in GSE55235. **B** Differential expression of PDK1 in GSE98918. **C** The PPI network for PDK1 was constructed in GeneMANIA. **D** KEGG analysis for genes that highly correlated with PDK1. **E** GO analysis for genes that highly correlated with PDK1

We further analyzed the expression of PDK1, a key gene in the process of glucose metabolism. First, the mRNA level of PDK1 in OA samples and normal samples of the GEO database was compared, and we found that PDK1 was significantly downregulated in OA samples (Fig. 2A and B). Afterward, the GeneMANIA online tool was used to explore the co-expression network of genes highly correlated with PDK1, and the result is shown in Fig. 2C. As vividly shown in the figure, 20 target genes were identified. Next, to further investigate these 20 target genes, GO and KEGG enrichment analysis was performed. As shown in Fig. 2D and E, the significant functions were the Citrate cycle, Pyruvate metabolism, Glycolysis/Gluconeogenesis in KEGG analysis, and acetyl-CoA biosynthetic process from pyruvate, acetyl-CoA biosynthetic process, acetyl-CoA metabolic process, mitochondrial matrix, and mitochondrial protein-containing complex in GO analysis.

### PDK1 is downregulated in human and mouse osteoarthritic cartilage

**Fig. 3:**
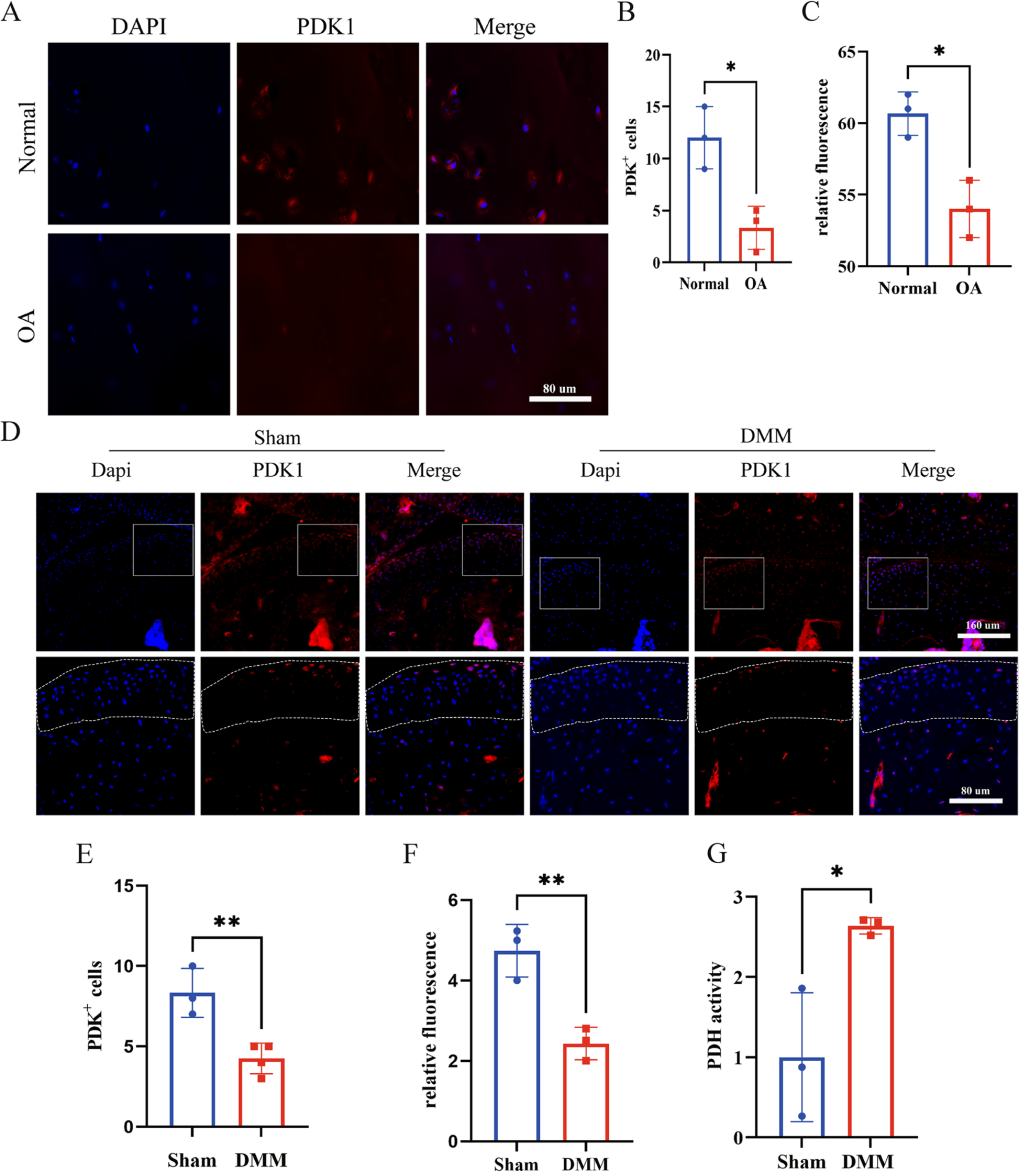
PDK1 is downregulated in human and mice osteoarthritic cartilage. **A** Immunofluorescence staining of PDK1 in articular cartilage from healthy and OA human. PDK1 stains red. Dapi stains the nucleus. **B** Quantification of an absolute number of PDK1.^+^ cells in articular cartilage from human. **C** Quantification analysis of the fluorescence intensity of PDK1 expression in articular cartilage from human. **D** Immunofluorescence staining of PDK1 in articular cartilage at sham or 4 weeks post-surgery. PDK1 stains red. Dapi stains the nucleus. **E** Quantifying an absolute number of PDK1 + cells in upper articular cartilage in the knee at the sham or 4 weeks post-surgery. **F** Quantification analysis of the fluorescence intensity of PDK1 expression in articular cartilage in the knee at sham or 4 weeks post-surgery. **G** PDH activity in articular cartilage at sham or 4 weeks post-surgery. In **B**, **C**, and **E**–**G**, horizontal lines and error bars show the mean ± SD (*n* ≥ 3 mice per group). * = *P* < 0.05; ** = *P* < 0.01; *** = *P* < *0.001*

To further confirm the relationship between PDK1 and OA, immunofluorescence performed in Human and mouse joint sections revealed reduced PDK1 protein expression in osteoarthritic chondrocytes (Fig. 3A-F). As shown in Fig. 3A-C, in human sample, OA cartilage exhibited weakened PDK1 compared to normal group. Similarly, DMM group mice displayed diminished PDK1 compared to sham group in articular cartilage (Fig. 3D-F). In addition, the Enhanced activity of PDH was detected in osteoarthritic articular cartilage (Fig. 3G).

### Inhibition of PDK1 promotes DMM-induced OA in mice

**Fig. 4:**
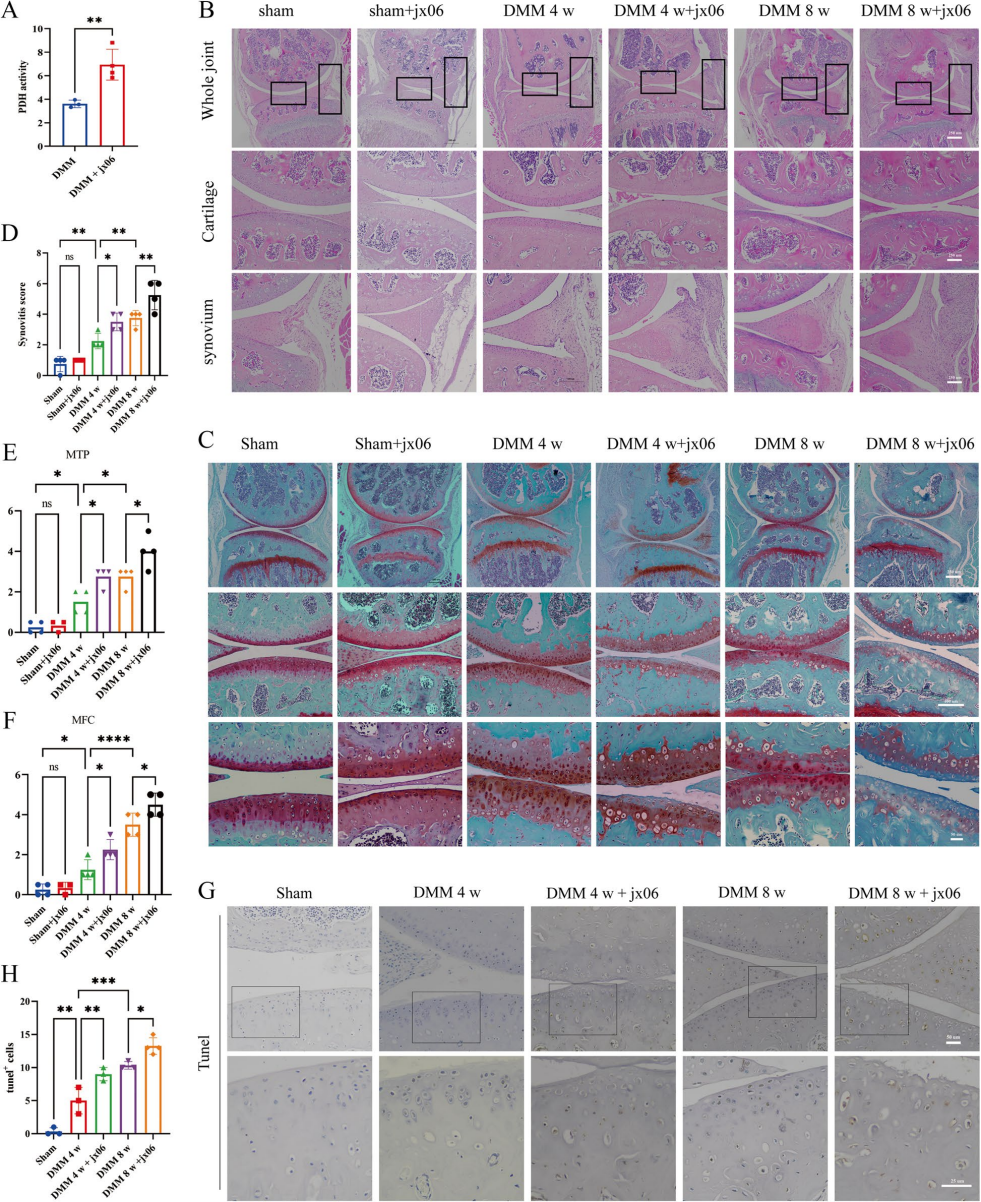
Inhibition of PDK1 aggravated pathological changes in joints. **A** PDH activity in articular cartilage at 8 weeks post-surgery with or without jx06. **B** Representative images of H&E staining of knee joints from mice at sham,4 or 8 weeks after DMM with or without jx06. **C** Representative images of Safranin o/Fast Green-stained sections of knee joints from mice at sham,4 or 8 weeks after DMM with or without jx06. **D** Synovitis score in the synovium of mice at sham,4 or 8 weeks after DMM with or without jx06. **E** and **F** OARSI score of knee joint cartilage at sham, 4 or 8 weeks after DMM with or without jx06, MFC: medial femoral condyle; MTP: medial tibial plateau. **G** Representative images of tunel staining for mice at sham,4 or 8 weeks after DMM with or without jx06. **H** Quantification of absolute number of tunel.^+^ cells in upper articular cartilage in the knee at sham or 4 weeks post-surgery. In **C**, **E**, **F** and **H**, horizontal lines and error bars show the mean ± SD (*n* ≥ 3 mice per group). * = *P* < 0.05; ** = *P* < 0.01; *** = *P* < *0.001*

Next, we investigated whether intra-articular injection of an inhibitor of PDK (jx06) could affect the pathogenesis of OA after DMM in mice. DMM surgery was performed in 2-month-old male C57 wild-type mice with or without jx06 administration commencing either 1 or 5 weeks after DMM surgery. As expected, jx06 significantly enhanced the activity of PDH in articular chondrocytes (Fig. 4A). Furthermore, histological analysis was performed to evaluate OA progression, including synovial tissue hyperplasia and articular cartilage degeneration. Increased synovial lining cell layer and enhanced inflammatory infiltration were observed in the jx06 treatment group subjected to DMM surgery (Fig. 4B and D). An accelerated proteoglycan loss (assess uled by safranin O-fast green) was observed in the jx06 treatment group subjected to DMM surgery (Fig. 4C). Similarly, jx06 treated mice had significantly higher score in medial femoral condyle (MFC) and medial tibial plateau (MTP) (Fig. 4E, and F). Although jx06 intervention alone, without DMM surgery, did not affect proteoglycan loss and inflammatory infiltration (Fig. 4B-F). Moreover, by tunnel staining, mice injected with jx06 showed a significant increase of apoptosis in cartilage (Fig. 4G and H).

### Inhibition of PDK1 affects the matrix anabolism and catabolism in cartilage

**Fig. 5:**
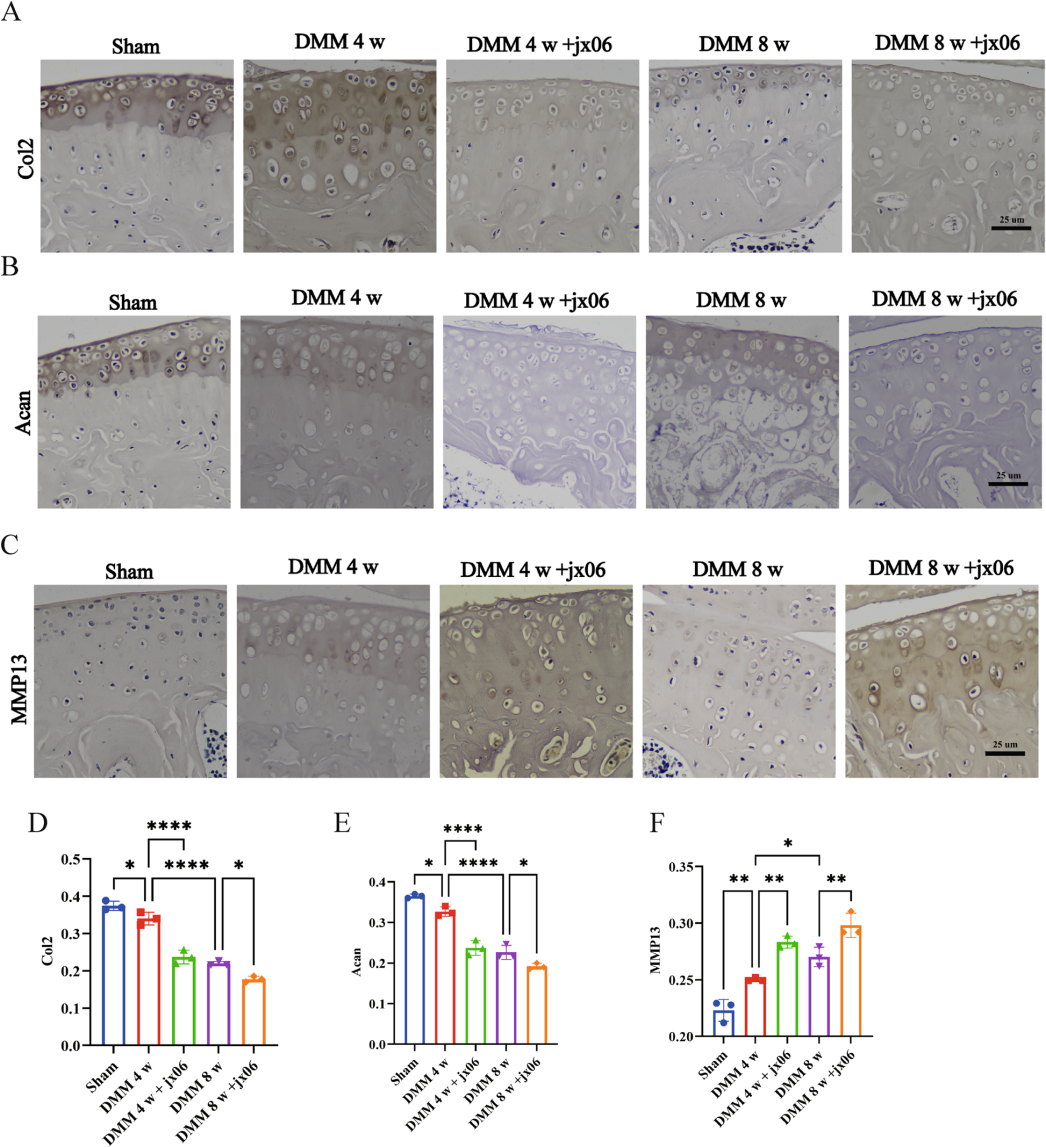
Suppressing of PDK1 disrupted anabolism and catabolism. **A**-**C** Representative immunohistochemistry images of Col2, Acan, MMP13 staining for mice at sham,4 or 8 weeks after DMM with or without jx06. **D**-**F** The mean ratio of integrated optical density (IOD) to area (IOD/area) was used to semi-quantify Col2, Acan, MMP13 amount, horizontal lines and error bars show the mean ± SD (*n* ≥ 3 mice per group). * = *P* < 0.05; ** = *P* < 0.01; *** = *P* < *0.001*

To assess the possible mechanism of PDK1 inhibitor on OA progression, we intraarticular injected jx06 into DMM-induced OA mice. To investigate whether jx06 could modulate ECM degradation by stimulating anabolism and catabolism during OA progression, we performed immunohistochemical staining on paraffin sections. We observed that jx06 reduced the type II collagen (Col2) (Fig. 5A and D), aggrecan (Acan) (Fig. 5B and E), and enhanced matrix metalloproteinase (MMP13) (Fig. 5C and F).

### Inhibition of PDK1 accelerates infiltration of macrophages, enhances the production of inflammatory mediators

**Fig. 6:**
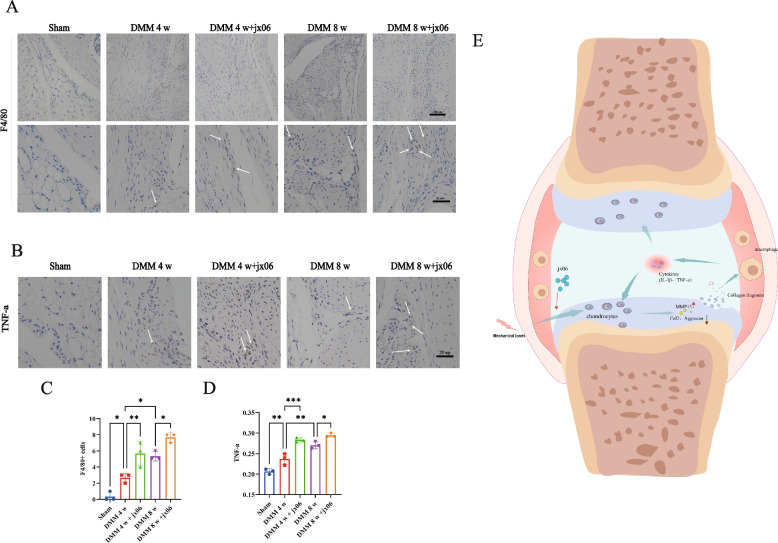
Suppressing of PDK1 accelerated synovium inflammation. **A** Representative immunohistochemistry images of F4/80 staining for mice at sham,4 or 8 weeks after DMM with or without jx06. **B** Representative immunohistochemistry images of TNF-a staining for mice at sham,4 or 8 weeks after DMM with or without jx06. **C** Quantification of absolute number of F4/80.^+^ cells in synovium in the knee at sham or 4 weeks post-surgery. **D** The mean ratio of integrated optical density (IOD) to area (IOD/area) was used to semi-quantify TNF-a amount. **E** Model of PDK1 inhibitor modulates ECM degradation and synovium inflammation in mechanical stress-induced OA. Horizontal lines and error bars show the mean ± SD (*n* ≥ 3 mice per group). * = *P* < 0.05; ** = *P* < 0.01; *** = *P* < *0.001*

Macrophage infiltration of lubricating membranes is one of the important events in OA development. Immunohistochemical staining analysis showed enhanced F4/80 positive macrophage in the synovium of jx06-treated mice compared with the DMM-induced group (Fig. 6A and C). Furthermore, as shown in Fig. 6B and D, the protein level of synovial TNF-a in the jx06 treatment group was increased.

## Discussion

OA is considered the most common joint disease [23]. The disease is first manifested by abnormal metabolism of joint tissue, followed by cartilage degeneration, joint inflammation, and loss of normal joint function, which can eventually lead to disease [1]. In this study, through GSEA analysis, we showed that Citrate cycle TCA cycle, pyruvate metabolism, and oxidative phosphorylation gene sets were enriched in OA. Moreover, the mRNA level of PDK1 was reduced based on GEO, and genes, highly correlated with PDK1, were mainly enriched in metabolism-related pathways. Besides, the protein levels of PDK1 were reduced in OA. After establishing the DMM-induced osteoarthritis, we showed that the inhibition of PDK1 did promote OA progression by enhancing the expression of MMP13, accelerating the degradation of major ECM structural molecules, and promoting inflammatory responses. Our findings indicated that the inhibition of PDK1 worsens osteoarthritis by mediating the degradation of articular cartilage ECM and inflammation.

Due to biomechanical and inflammatory stimuli, OA is driven by the imbalance of anabolic and catabolic in chondrocytes. Here, we report the effects of metabolism shift on osteoarthritis and present definitive data that PDK1 activity is one of the major drivers of pathological changes in OA. Chondrocytes are damaged by metabolism shifts in response to mechanical stress. The damaged chondrocytes can produce and release damage-associated molecular patterns (DAMPs). Next, the DAMPs act on the surrounding chondrocytes, and stimulate macrophages in the synovial tissue, exacerbating the inflammatory stimulation of the synovial space, resulting in further damage to the chondrocytes and creating a malignant positive feedback cycle (Fig. 6e).

Metabolic disorders can lead to the imbalance of anabolic and catabolic in chondrocytes. For example, cholesterol levels in osteoarthritic chondrocytes are elevated due to the upregulation of cholesterol hydroxylase and increased production of oxysterol metabolites [24]. Besides, expression of the major glucose transporter Glut1 is significantly reduced in osteoarthritic chondrocytes, and deletion of Glut1 could accelerate cartilage loss [11, 25]. Through GSEA we showed that pyruvate metabolism, oxidative phosphorylation pathway, and citrate cycle TCA cycle were significantly enriched in OA, indicating the imbalance between glycolysis and OXPHOS may link to OA. Besides, PDH acts as a gatekeeper linking glycolysis to the TCA cycle, maintaining metabolic homeostasis and energy production through the rate-limiting of pyruvate [14]. In addition, we found that genes, highly related to PDK1, are mainly enriched in metabolism-related pathways, indicating that PDK1 may affect metabolism by acting on important genes in metabolic pathways. Phosphorylation of the PDH-E1α subunit by PDK results in decreased PDH activity [26]. Therefore, PDK activity blocks pyruvate fluxes into the TCA cycle, causing metabolism to shift to glycolysis to produce energy [14]. In our study, protein kinase activity-related genes were downregulated in the OA group. The protein level of PDK1 was significantly decreased in both human and mice OA chondrocytes. As expected, the activity of PDH was increased in OA. Exacerbated OA was observed in DMM-induced mice after being treated with an inhibitor of PDK1. As a result, osteoarthritis may be exacerbated by enhancing the activity of PDH in articular chondrocytes through the inhibition of PDK1.

To date, some studies have found that many small molecules can affect OA progression by targeting ECM homeostasis [3, 27]. Cartilage chondrocytes are surrounded by ECM [5]. Col2 and Acan, the most abundant proteoglycan in cartilage, are important components of ECM [28]. MMPs are capable of degrading a variety of ECM proteins [29]. The ECM changes in osteoarthritis appear to be caused by an imbalance in the anabolic and catabolic chondrocytes [6]. Under adverse stimulation, chondrocytes secrete MMP13 in large quantities, while Col2 and Acan synthesis decreases [30]. In our study, mice were treated with intra-articular injection of an inhibitor of PDK1. A few weeks later, we found that inhibiting PDK downregulated the expression of Col2 and Acan in DMM-induced osteoarthritic chondrocytes, and had a promoting effect on MMP13. Enzymatic cartilage degradation leads to the massive production of ECM fragments. These fragments can be recognized by pattern recognition receptors (PRRs) in macrophages [31]. Pro-inflammatory mediators, such as TNF-a, were increased in OA synovium [32]. Once activated by DAMPs, macrophages can release pro-inflammatory mediators, which lead to the recruitment of inflammatory cells [33]. In our experiment, hyperplasia of the synovial lining occurred in mice induced by DMM, possibly due to macrophage recruitment [32, 34]. In response to these DAMPs, macrophages could produce cytokines [35]. Subsequently, through interaction with chondrocytes, these cytokines may accelerate OA [36].

Growing evidence suggests that metabolism plays a key role in the regulation of inflammatory responses, with different cells exhibiting different metabolic signatures to modulate their biological responses [7]. In our study, we present definitive data that PDK1 activity is one of the major drivers of pathological changes in osteoarthritic chondrocytes. OA is a disease mediated by the entire “joint organ”, including the articular cartilage and the synovium [37]. Recent studies have revealed the role of PDKs in synovitis. Ma JD et al. showed that inhibition of PDK1 by an inhibitor of PDK1 or siRNA via suppression of expression of MMP-2 and MMP-9 inhibited RA- fibroblast-like synoviocytes (FLS) migration and invasion [38]. Similarly, Damerau A et al. showed that overexpressed PDK3 was observed in FLS in OA [39]. However, in our study, the protein level of PDK1 was decreased in osteoarthritic chondrocytes. Recent studies showed that jx06 could selectively inhibit PDK1 [12, 40]. Aggravated synovitis was observed in DMM-induced mice with jx06. RA-FLS undergo a shift from oxidative phosphorylation to glycolysis in ATP formation, which could be an adaptation to the joint microenvironment [41]. On the contrary, in healthy cartilage, chondrocytes rely on glycolysis to produce energy and biomass [42]. However, OA cartilage is characterized by reduced glycolysis and an increase in mitochondrial respiration [43, 44].

## Conclusion

In conclusion, we confirmed that PDK1 inhibitor exacerbated the development of OA, and the underlying mechanism might be related to the glucose metabolism disorder, which leads to an imbalance of anabolic and catabolic processes of chondrocytes and accelerates inflammation. It is reasonable to expect that targeting PDK1 may be a promising strategy for improving OA outcomes.

## Supplementary Information

**Additional file 1.**

## Data Availability

The datasets are available from the corresponding author.
