# Supplementary figures and images for: Pyruvate Dehydrogenase Kinase 1 inhibition mediated oxidative phosphorylation enhancement in cartilage promotes osteoarthritis progression (part 2 of 2)
Source: BMC Musculoskelet Disord. 2023 Jul 20;24:597. doi: 10.1186/s12891-023-06585-6 (PMC10357736; doi:10.1186/s12891-023-06585-6)

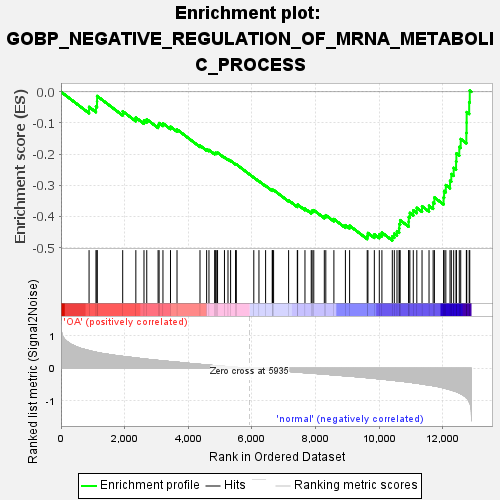

Supplement: Supplementary file 1 — Additional file 1. [file 12891_2023_6585_MOESM1_ESM.zip › BP.Gsea.1653623667859/enplot_GOBP_NEGATIVE_REGULATION_OF_MRNA_METABOLIC_PROCESS_860.png]

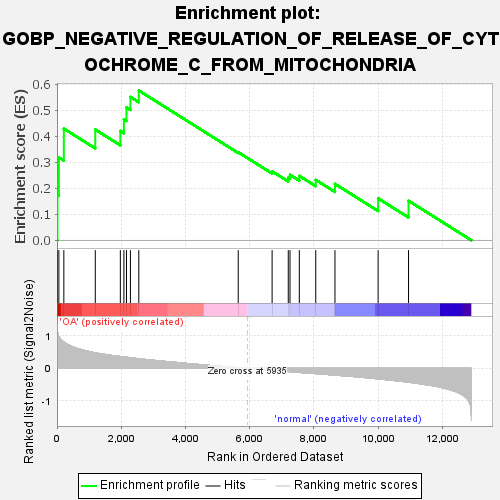

Supplement: Supplementary file 1 — Additional file 1. [file 12891_2023_6585_MOESM1_ESM.zip › BP.Gsea.1653623667859/enplot_GOBP_NEGATIVE_REGULATION_OF_RELEASE_OF_CYTOCHROME_C_FROM_MITOCHONDRIA_605.png]

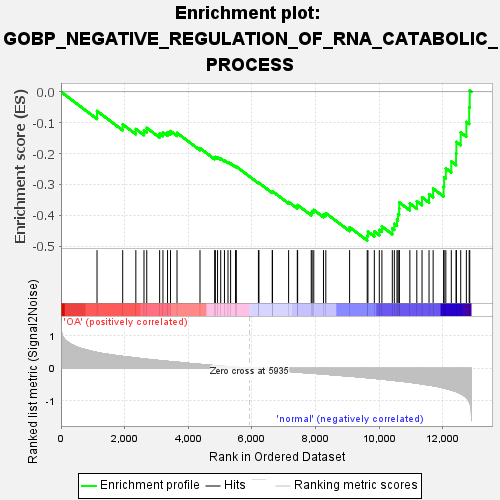

Supplement: Supplementary file 1 — Additional file 1. [file 12891_2023_6585_MOESM1_ESM.zip › BP.Gsea.1653623667859/enplot_GOBP_NEGATIVE_REGULATION_OF_RNA_CATABOLIC_PROCESS_881.png]

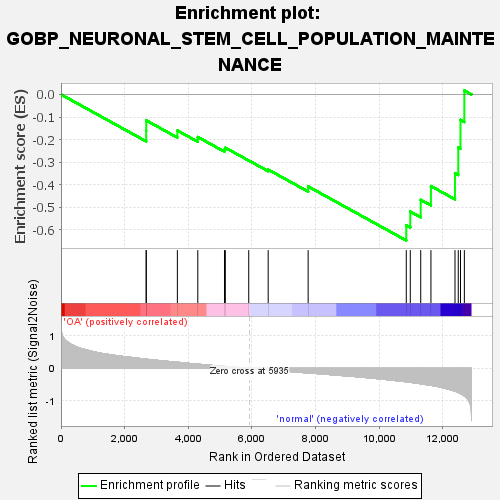

Supplement: Supplementary file 1 — Additional file 1. [file 12891_2023_6585_MOESM1_ESM.zip › BP.Gsea.1653623667859/enplot_GOBP_NEURONAL_STEM_CELL_POPULATION_MAINTENANCE_893.png]

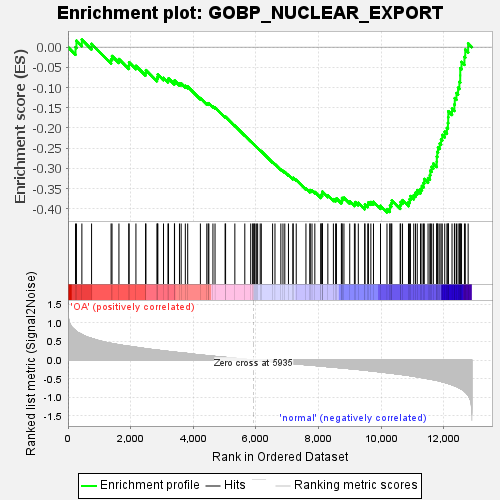

Supplement: Supplementary file 1 — Additional file 1. [file 12891_2023_6585_MOESM1_ESM.zip › BP.Gsea.1653623667859/enplot_GOBP_NUCLEAR_EXPORT_911.png]

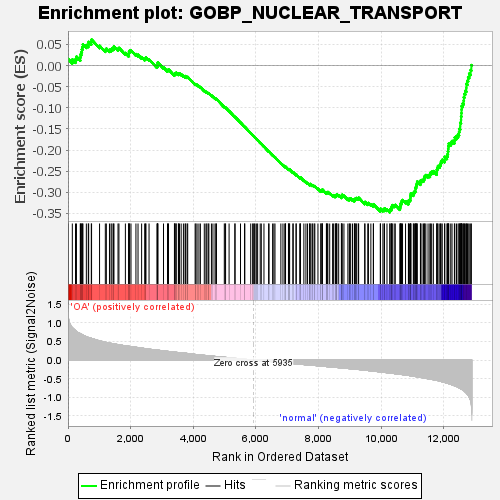

Supplement: Supplementary file 1 — Additional file 1. [file 12891_2023_6585_MOESM1_ESM.zip › BP.Gsea.1653623667859/enplot_GOBP_NUCLEAR_TRANSPORT_1097.png]

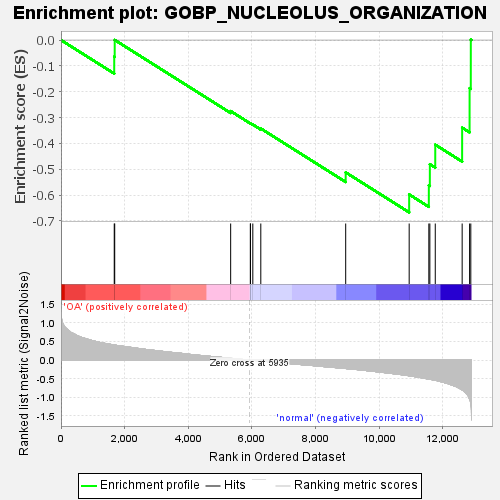

Supplement: Supplementary file 1 — Additional file 1. [file 12891_2023_6585_MOESM1_ESM.zip › BP.Gsea.1653623667859/enplot_GOBP_NUCLEOLUS_ORGANIZATION_896.png]

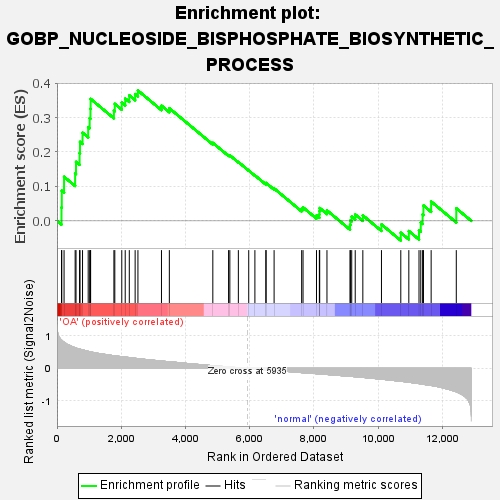

Supplement: Supplementary file 1 — Additional file 1. [file 12891_2023_6585_MOESM1_ESM.zip › BP.Gsea.1653623667859/enplot_GOBP_NUCLEOSIDE_BISPHOSPHATE_BIOSYNTHETIC_PROCESS_692.png]

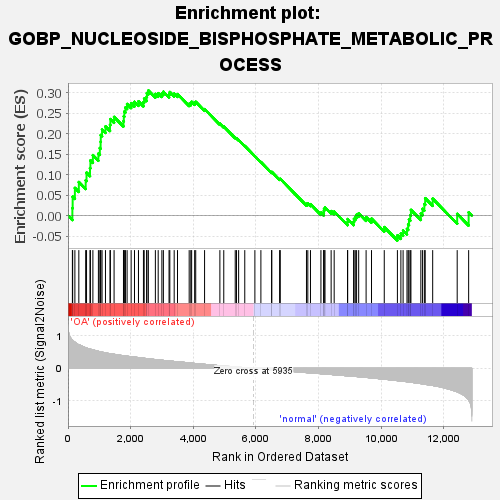

Supplement: Supplementary file 1 — Additional file 1. [file 12891_2023_6585_MOESM1_ESM.zip › BP.Gsea.1653623667859/enplot_GOBP_NUCLEOSIDE_BISPHOSPHATE_METABOLIC_PROCESS_806.png]

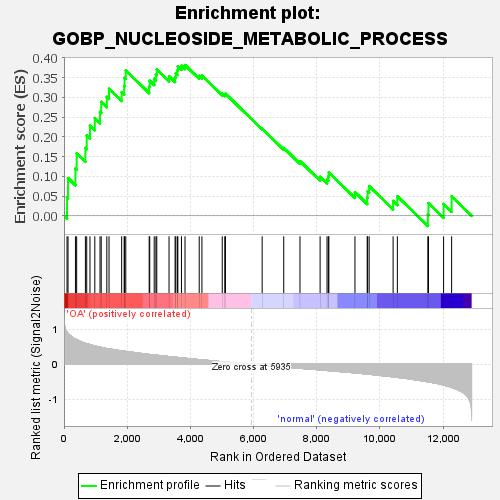

Supplement: Supplementary file 1 — Additional file 1. [file 12891_2023_6585_MOESM1_ESM.zip › BP.Gsea.1653623667859/enplot_GOBP_NUCLEOSIDE_METABOLIC_PROCESS_701.png]

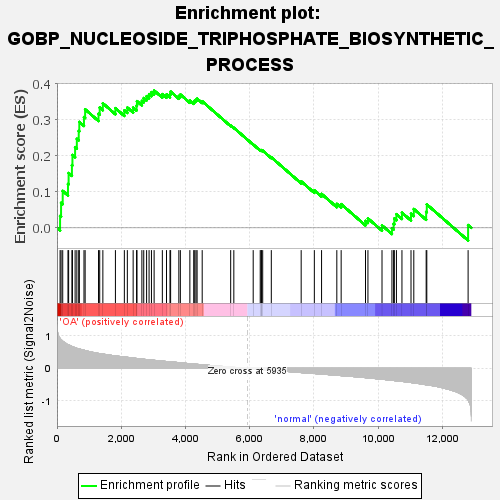

Supplement: Supplementary file 1 — Additional file 1. [file 12891_2023_6585_MOESM1_ESM.zip › BP.Gsea.1653623667859/enplot_GOBP_NUCLEOSIDE_TRIPHOSPHATE_BIOSYNTHETIC_PROCESS_647.png]

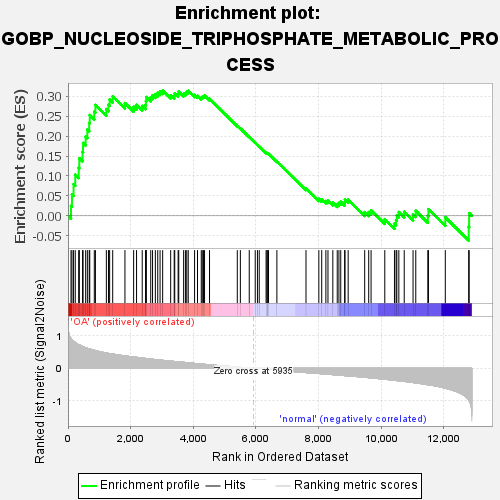

Supplement: Supplementary file 1 — Additional file 1. [file 12891_2023_6585_MOESM1_ESM.zip › BP.Gsea.1653623667859/enplot_GOBP_NUCLEOSIDE_TRIPHOSPHATE_METABOLIC_PROCESS_800.png]

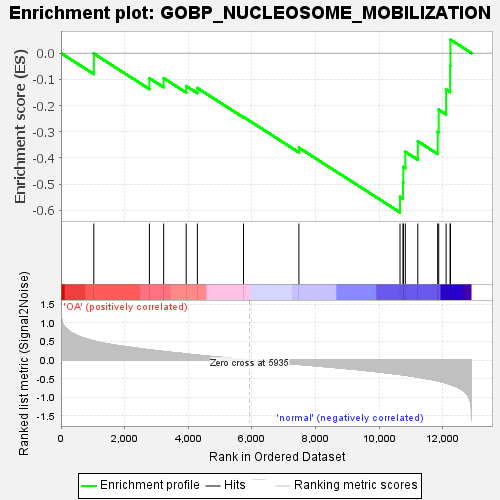

Supplement: Supplementary file 1 — Additional file 1. [file 12891_2023_6585_MOESM1_ESM.zip › BP.Gsea.1653623667859/enplot_GOBP_NUCLEOSOME_MOBILIZATION_1016.png]

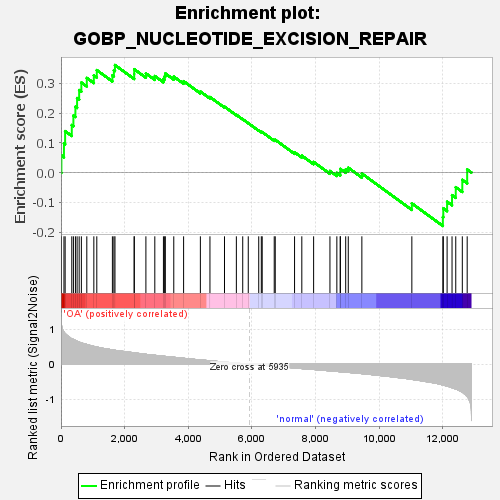

Supplement: Supplementary file 1 — Additional file 1. [file 12891_2023_6585_MOESM1_ESM.zip › BP.Gsea.1653623667859/enplot_GOBP_NUCLEOTIDE_EXCISION_REPAIR_746.png]

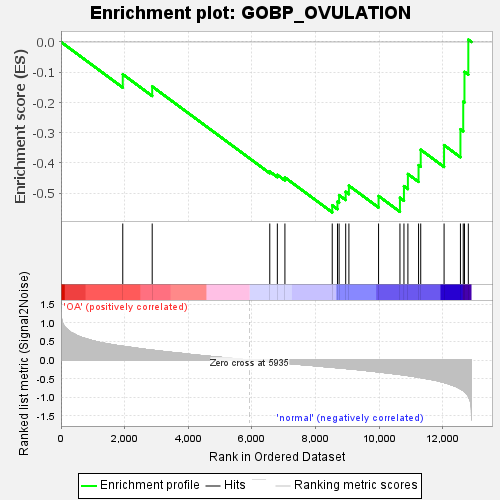

Supplement: Supplementary file 1 — Additional file 1. [file 12891_2023_6585_MOESM1_ESM.zip › BP.Gsea.1653623667859/enplot_GOBP_OVULATION_1001.png]

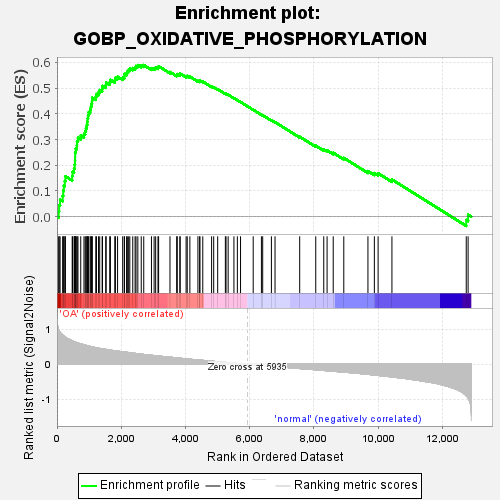

Supplement: Supplementary file 1 — Additional file 1. [file 12891_2023_6585_MOESM1_ESM.zip › BP.Gsea.1653623667859/enplot_GOBP_OXIDATIVE_PHOSPHORYLATION_518.png]

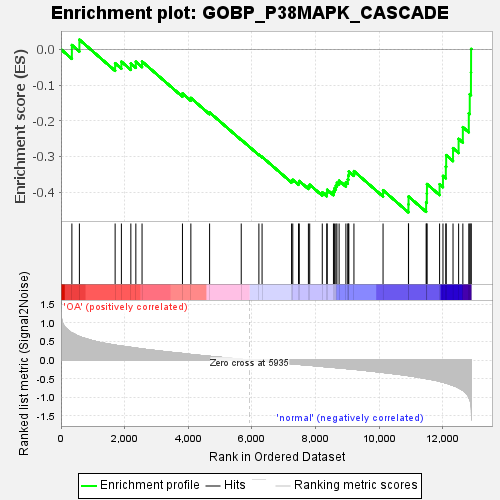

Supplement: Supplementary file 1 — Additional file 1. [file 12891_2023_6585_MOESM1_ESM.zip › BP.Gsea.1653623667859/enplot_GOBP_P38MAPK_CASCADE_995.png]

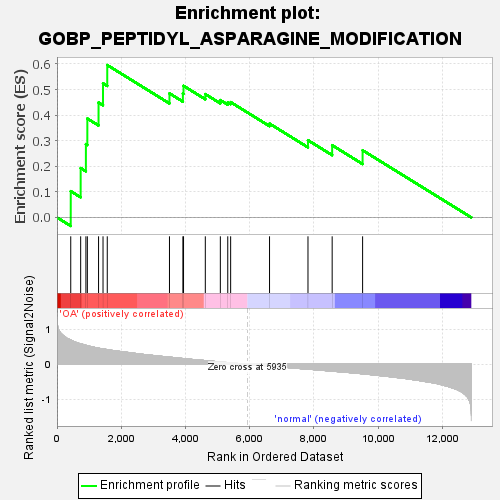

Supplement: Supplementary file 1 — Additional file 1. [file 12891_2023_6585_MOESM1_ESM.zip › BP.Gsea.1653623667859/enplot_GOBP_PEPTIDYL_ASPARAGINE_MODIFICATION_590.png]

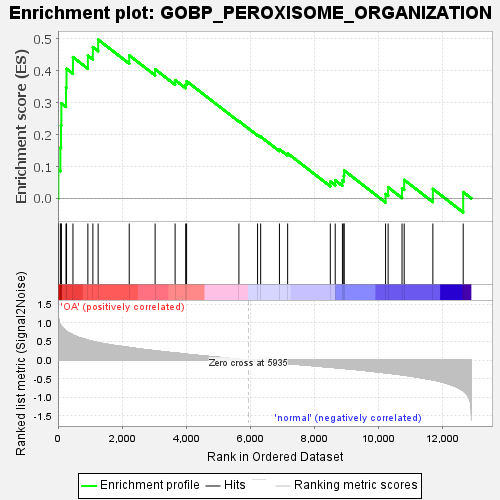

Supplement: Supplementary file 1 — Additional file 1. [file 12891_2023_6585_MOESM1_ESM.zip › BP.Gsea.1653623667859/enplot_GOBP_PEROXISOME_ORGANIZATION_596.png]

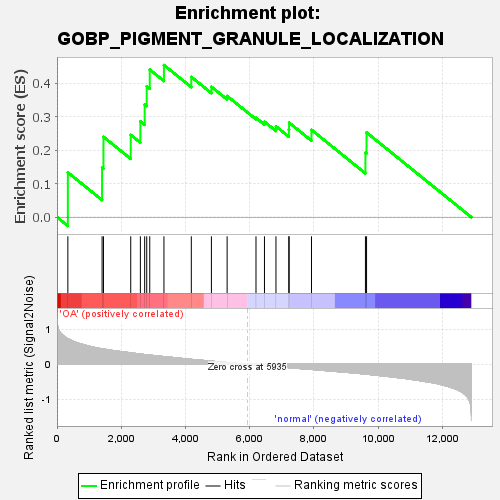

Supplement: Supplementary file 1 — Additional file 1. [file 12891_2023_6585_MOESM1_ESM.zip › BP.Gsea.1653623667859/enplot_GOBP_PIGMENT_GRANULE_LOCALIZATION_782.png]

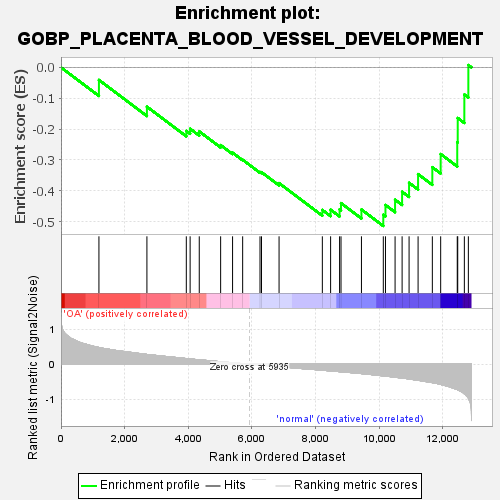

Supplement: Supplementary file 1 — Additional file 1. [file 12891_2023_6585_MOESM1_ESM.zip › BP.Gsea.1653623667859/enplot_GOBP_PLACENTA_BLOOD_VESSEL_DEVELOPMENT_1040.png]

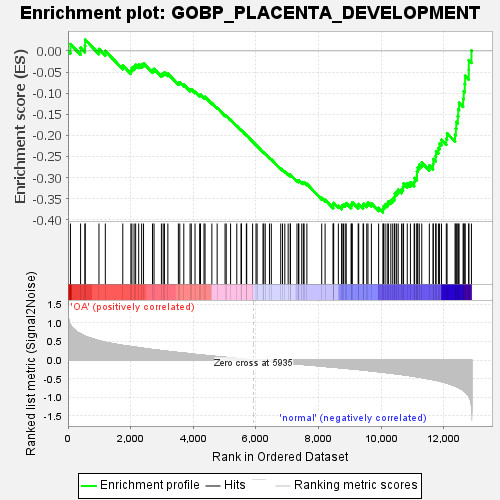

Supplement: Supplementary file 1 — Additional file 1. [file 12891_2023_6585_MOESM1_ESM.zip › BP.Gsea.1653623667859/enplot_GOBP_PLACENTA_DEVELOPMENT_1100.png]

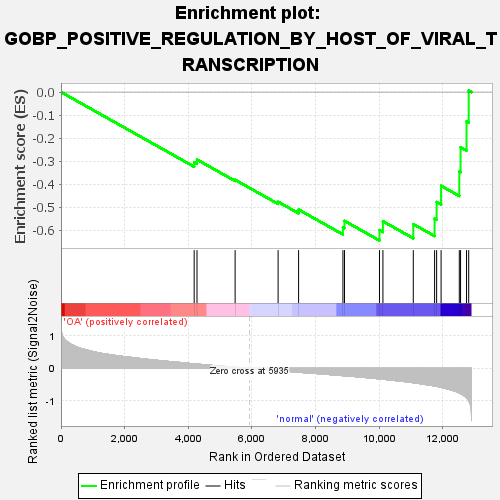

Supplement: Supplementary file 1 — Additional file 1. [file 12891_2023_6585_MOESM1_ESM.zip › BP.Gsea.1653623667859/enplot_GOBP_POSITIVE_REGULATION_BY_HOST_OF_VIRAL_TRANSCRIPTION_899.png]

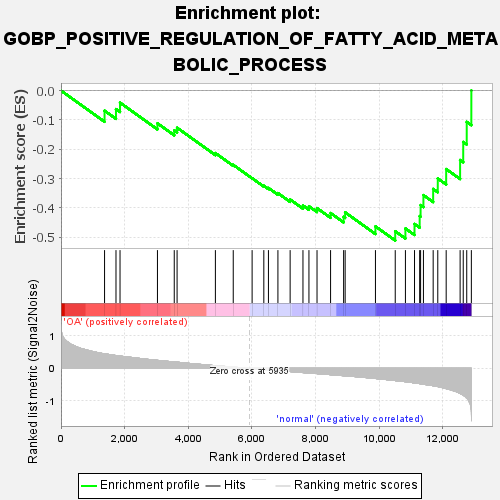

Supplement: Supplementary file 1 — Additional file 1. [file 12891_2023_6585_MOESM1_ESM.zip › BP.Gsea.1653623667859/enplot_GOBP_POSITIVE_REGULATION_OF_FATTY_ACID_METABOLIC_PROCESS_998.png]

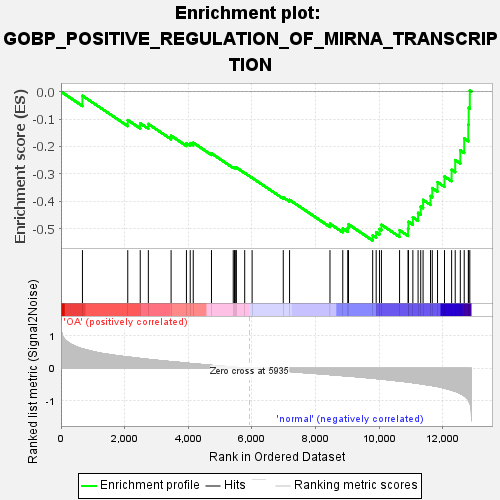

Supplement: Supplementary file 1 — Additional file 1. [file 12891_2023_6585_MOESM1_ESM.zip › BP.Gsea.1653623667859/enplot_GOBP_POSITIVE_REGULATION_OF_MIRNA_TRANSCRIPTION_845.png]

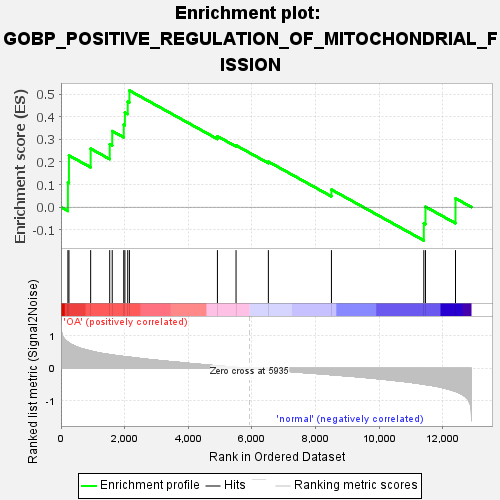

Supplement: Supplementary file 1 — Additional file 1. [file 12891_2023_6585_MOESM1_ESM.zip › BP.Gsea.1653623667859/enplot_GOBP_POSITIVE_REGULATION_OF_MITOCHONDRIAL_FISSION_674.png]

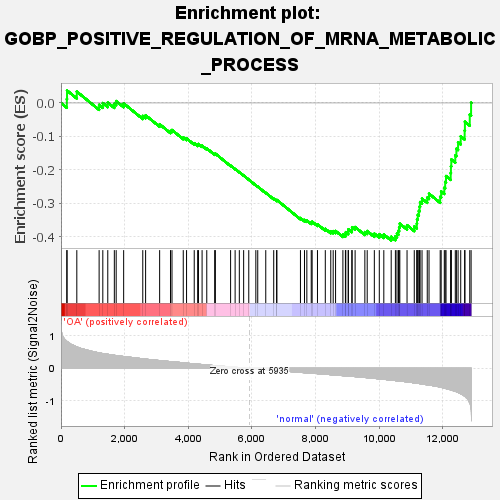

Supplement: Supplementary file 1 — Additional file 1. [file 12891_2023_6585_MOESM1_ESM.zip › BP.Gsea.1653623667859/enplot_GOBP_POSITIVE_REGULATION_OF_MRNA_METABOLIC_PROCESS_989.png]

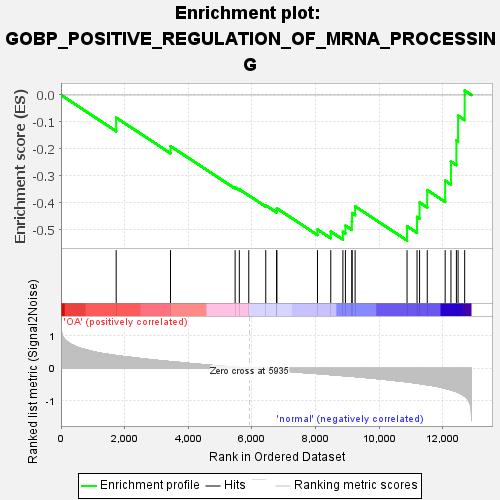

Supplement: Supplementary file 1 — Additional file 1. [file 12891_2023_6585_MOESM1_ESM.zip › BP.Gsea.1653623667859/enplot_GOBP_POSITIVE_REGULATION_OF_MRNA_PROCESSING_1019.png]

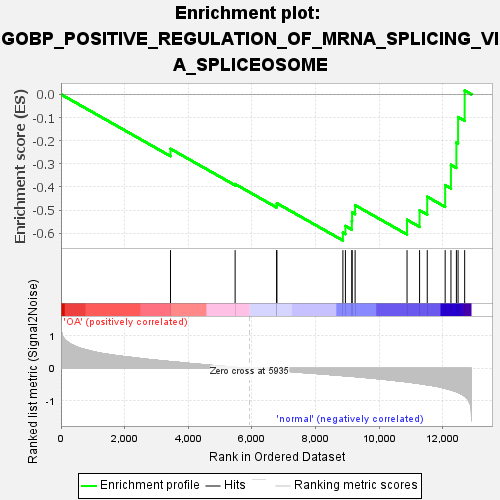

Supplement: Supplementary file 1 — Additional file 1. [file 12891_2023_6585_MOESM1_ESM.zip › BP.Gsea.1653623667859/enplot_GOBP_POSITIVE_REGULATION_OF_MRNA_SPLICING_VIA_SPLICEOSOME_926.png]

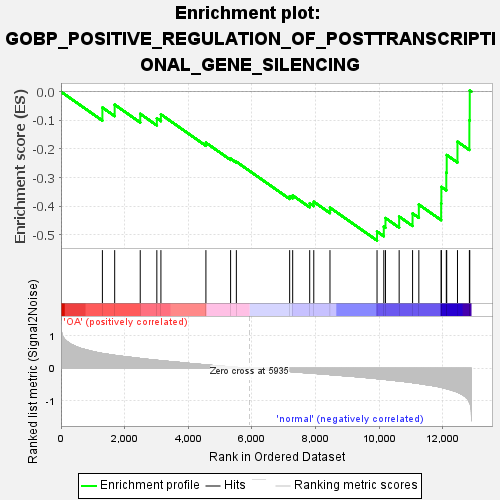

Supplement: Supplementary file 1 — Additional file 1. [file 12891_2023_6585_MOESM1_ESM.zip › BP.Gsea.1653623667859/enplot_GOBP_POSITIVE_REGULATION_OF_POSTTRANSCRIPTIONAL_GENE_SILENCING_1085.png]

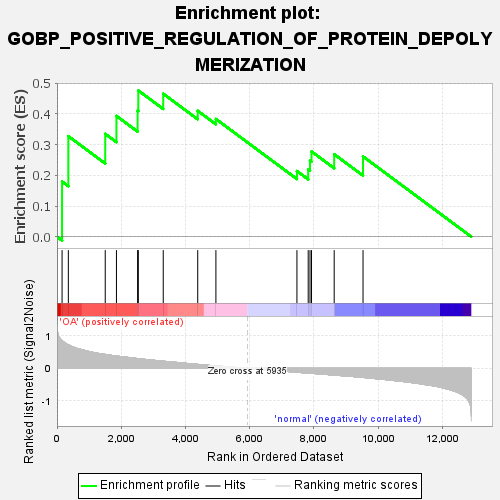

Supplement: Supplementary file 1 — Additional file 1. [file 12891_2023_6585_MOESM1_ESM.zip › BP.Gsea.1653623667859/enplot_GOBP_POSITIVE_REGULATION_OF_PROTEIN_DEPOLYMERIZATION_803.png]

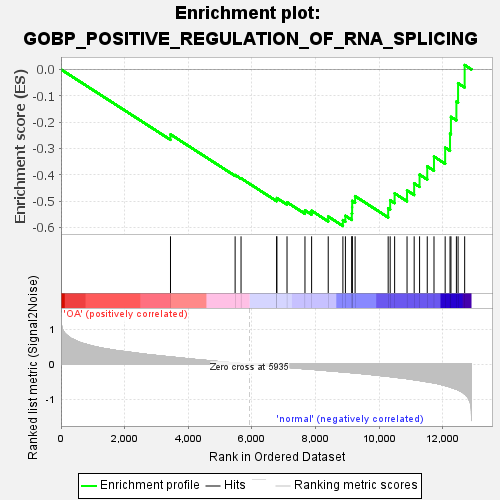

Supplement: Supplementary file 1 — Additional file 1. [file 12891_2023_6585_MOESM1_ESM.zip › BP.Gsea.1653623667859/enplot_GOBP_POSITIVE_REGULATION_OF_RNA_SPLICING_839.png]

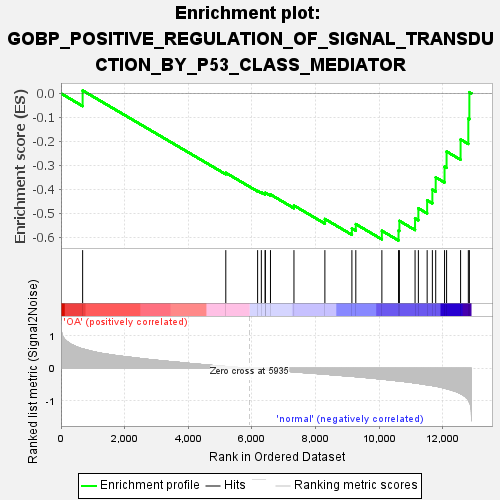

Supplement: Supplementary file 1 — Additional file 1. [file 12891_2023_6585_MOESM1_ESM.zip › BP.Gsea.1653623667859/enplot_GOBP_POSITIVE_REGULATION_OF_SIGNAL_TRANSDUCTION_BY_P53_CLASS_MEDIATOR_851.png]

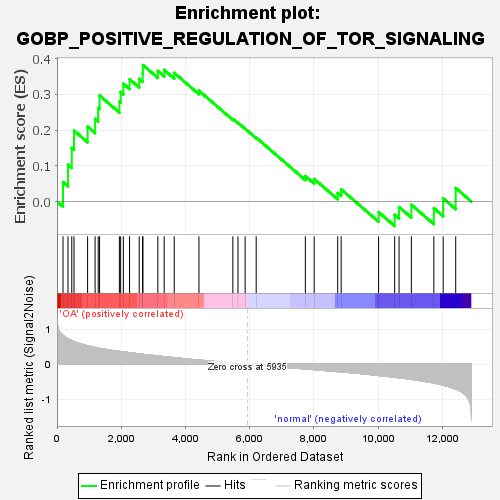

Supplement: Supplementary file 1 — Additional file 1. [file 12891_2023_6585_MOESM1_ESM.zip › BP.Gsea.1653623667859/enplot_GOBP_POSITIVE_REGULATION_OF_TOR_SIGNALING_794.png]

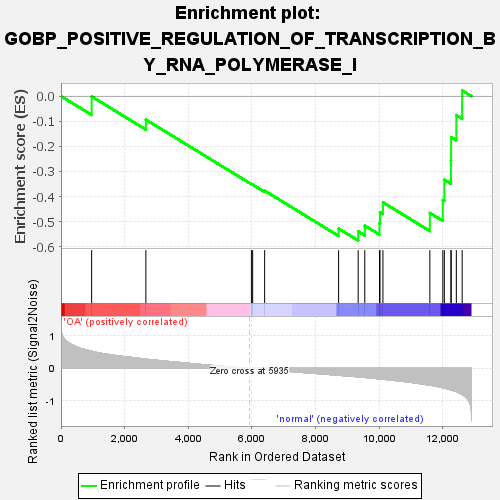

Supplement: Supplementary file 1 — Additional file 1. [file 12891_2023_6585_MOESM1_ESM.zip › BP.Gsea.1653623667859/enplot_GOBP_POSITIVE_REGULATION_OF_TRANSCRIPTION_BY_RNA_POLYMERASE_I_1109.png]

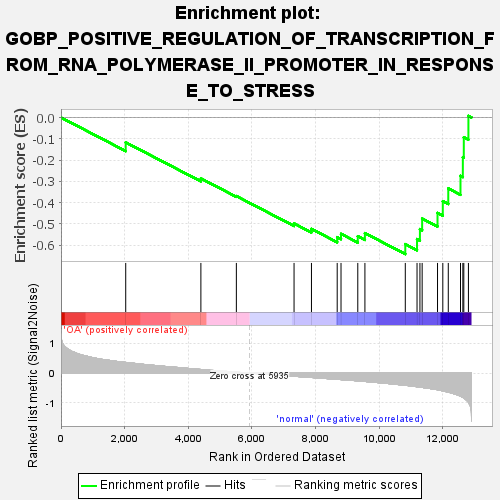

Supplement: Supplementary file 1 — Additional file 1. [file 12891_2023_6585_MOESM1_ESM.zip › BP.Gsea.1653623667859/enplot_GOBP_POSITIVE_REGULATION_OF_TRANSCRIPTION_FROM_RNA_POLYMERASE_II_PROMOTER_IN_RESPONSE_TO_STRE._866.png]

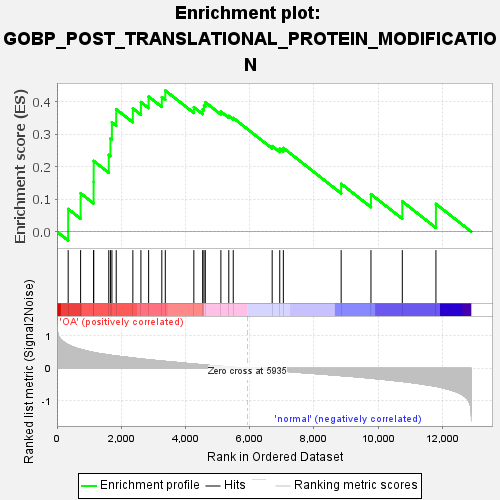

Supplement: Supplementary file 1 — Additional file 1. [file 12891_2023_6585_MOESM1_ESM.zip › BP.Gsea.1653623667859/enplot_GOBP_POST_TRANSLATIONAL_PROTEIN_MODIFICATION_731.png]

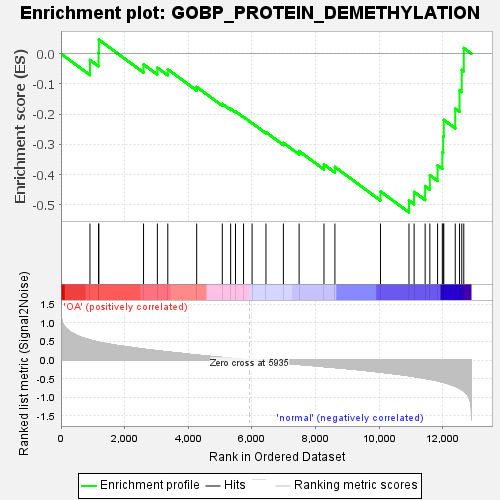

Supplement: Supplementary file 1 — Additional file 1. [file 12891_2023_6585_MOESM1_ESM.zip › BP.Gsea.1653623667859/enplot_GOBP_PROTEIN_DEMETHYLATION_992.png]

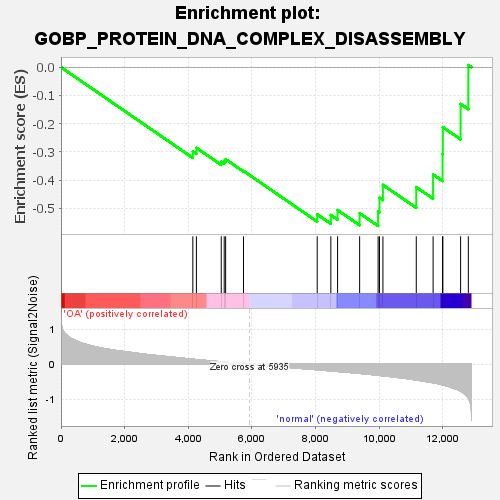

Supplement: Supplementary file 1 — Additional file 1. [file 12891_2023_6585_MOESM1_ESM.zip › BP.Gsea.1653623667859/enplot_GOBP_PROTEIN_DNA_COMPLEX_DISASSEMBLY_1070.png]

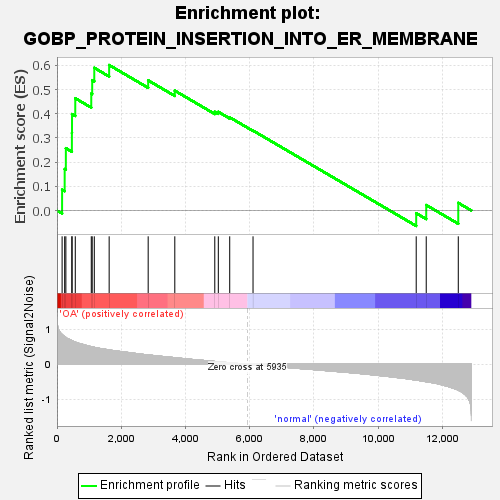

Supplement: Supplementary file 1 — Additional file 1. [file 12891_2023_6585_MOESM1_ESM.zip › BP.Gsea.1653623667859/enplot_GOBP_PROTEIN_INSERTION_INTO_ER_MEMBRANE_578.png]

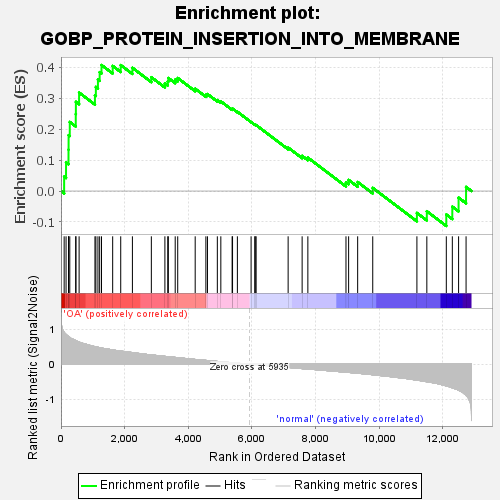

Supplement: Supplementary file 1 — Additional file 1. [file 12891_2023_6585_MOESM1_ESM.zip › BP.Gsea.1653623667859/enplot_GOBP_PROTEIN_INSERTION_INTO_MEMBRANE_638.png]

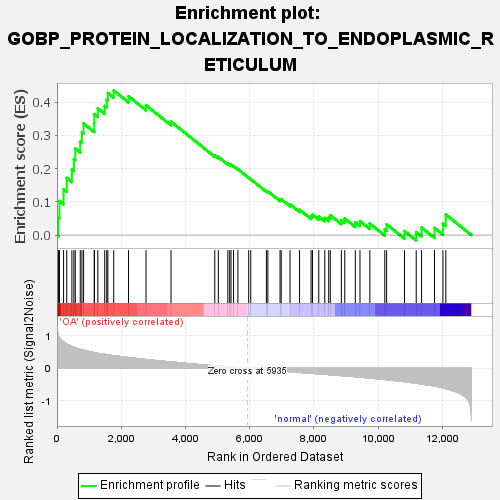

Supplement: Supplementary file 1 — Additional file 1. [file 12891_2023_6585_MOESM1_ESM.zip › BP.Gsea.1653623667859/enplot_GOBP_PROTEIN_LOCALIZATION_TO_ENDOPLASMIC_RETICULUM_608.png]

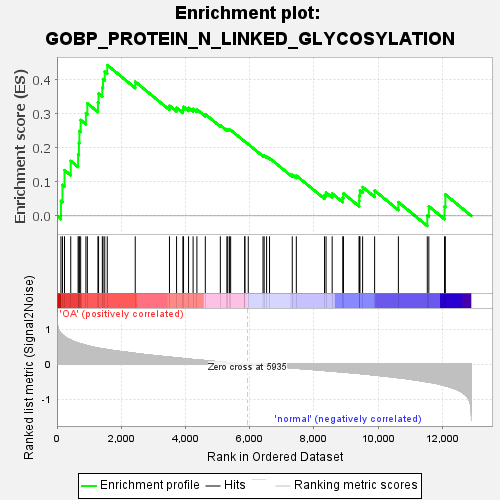

Supplement: Supplementary file 1 — Additional file 1. [file 12891_2023_6585_MOESM1_ESM.zip › BP.Gsea.1653623667859/enplot_GOBP_PROTEIN_N_LINKED_GLYCOSYLATION_599.png]

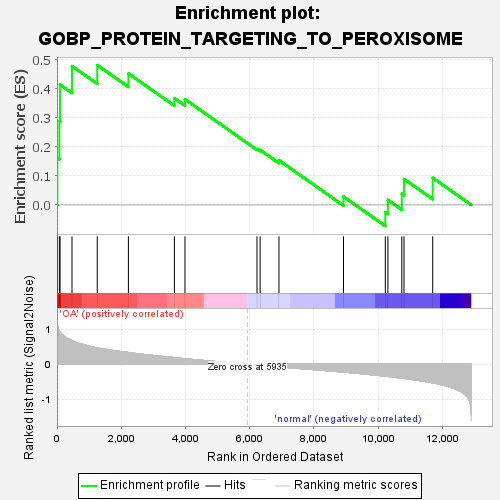

Supplement: Supplementary file 1 — Additional file 1. [file 12891_2023_6585_MOESM1_ESM.zip › BP.Gsea.1653623667859/enplot_GOBP_PROTEIN_TARGETING_TO_PEROXISOME_719.png]

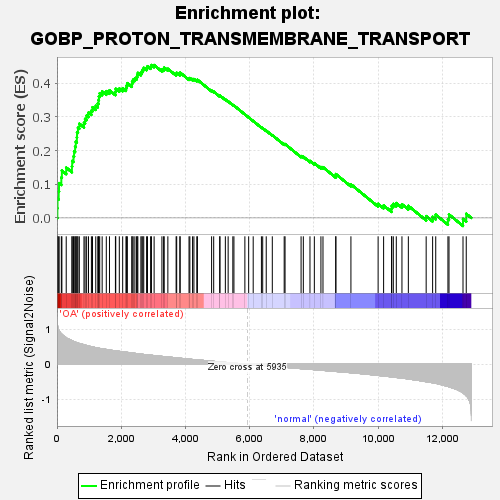

Supplement: Supplementary file 1 — Additional file 1. [file 12891_2023_6585_MOESM1_ESM.zip › BP.Gsea.1653623667859/enplot_GOBP_PROTON_TRANSMEMBRANE_TRANSPORT_542.png]

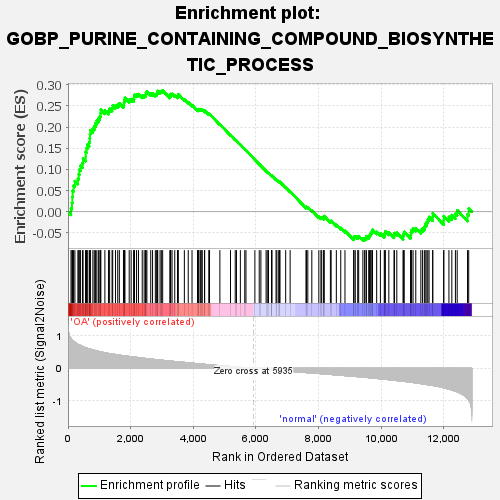

Supplement: Supplementary file 1 — Additional file 1. [file 12891_2023_6585_MOESM1_ESM.zip › BP.Gsea.1653623667859/enplot_GOBP_PURINE_CONTAINING_COMPOUND_BIOSYNTHETIC_PROCESS_785.png]

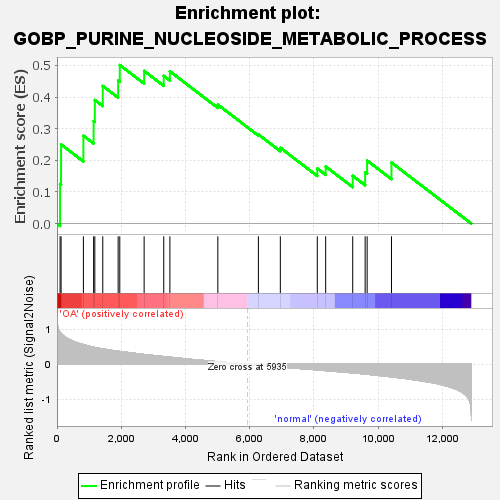

Supplement: Supplementary file 1 — Additional file 1. [file 12891_2023_6585_MOESM1_ESM.zip › BP.Gsea.1653623667859/enplot_GOBP_PURINE_NUCLEOSIDE_METABOLIC_PROCESS_668.png]

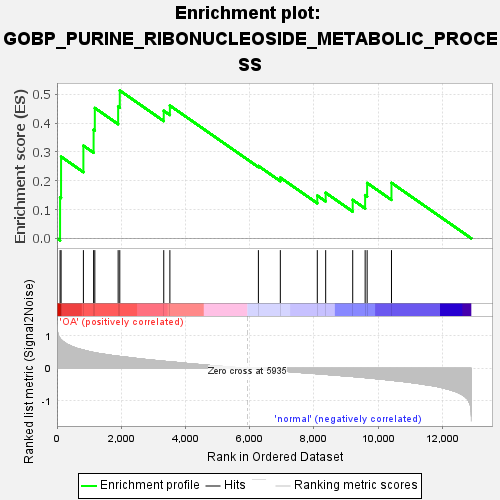

Supplement: Supplementary file 1 — Additional file 1. [file 12891_2023_6585_MOESM1_ESM.zip › BP.Gsea.1653623667859/enplot_GOBP_PURINE_RIBONUCLEOSIDE_METABOLIC_PROCESS_677.png]

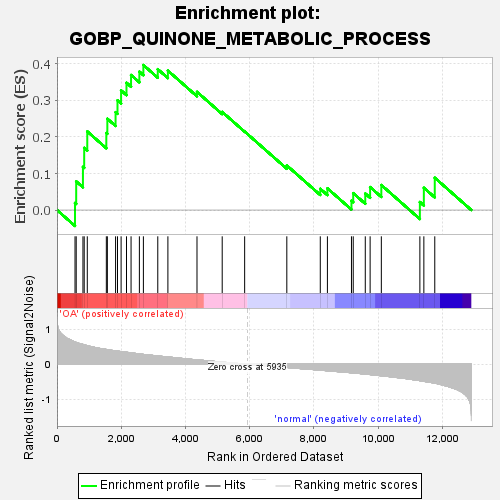

Supplement: Supplementary file 1 — Additional file 1. [file 12891_2023_6585_MOESM1_ESM.zip › BP.Gsea.1653623667859/enplot_GOBP_QUINONE_METABOLIC_PROCESS_809.png]

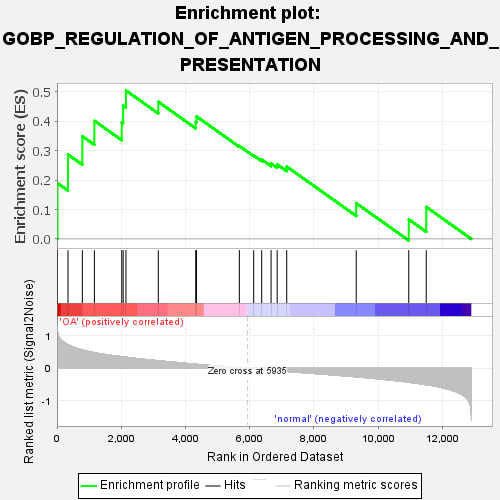

Supplement: Supplementary file 1 — Additional file 1. [file 12891_2023_6585_MOESM1_ESM.zip › BP.Gsea.1653623667859/enplot_GOBP_REGULATION_OF_ANTIGEN_PROCESSING_AND_PRESENTATION_704.png]

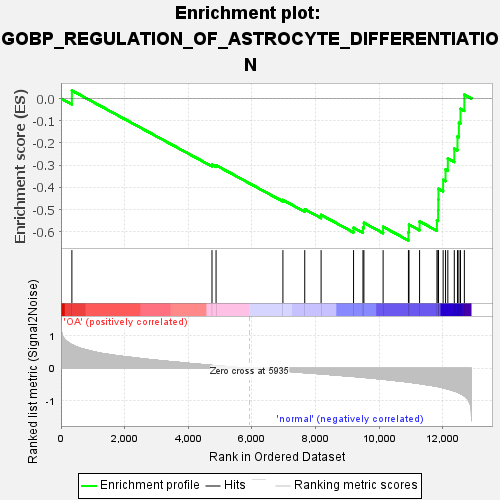

Supplement: Supplementary file 1 — Additional file 1. [file 12891_2023_6585_MOESM1_ESM.zip › BP.Gsea.1653623667859/enplot_GOBP_REGULATION_OF_ASTROCYTE_DIFFERENTIATION_836.png]

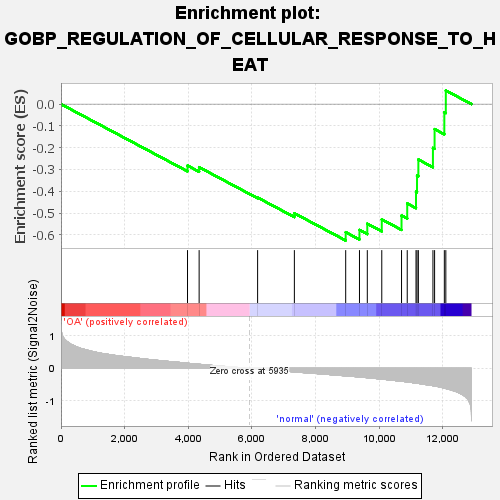

Supplement: Supplementary file 1 — Additional file 1. [file 12891_2023_6585_MOESM1_ESM.zip › BP.Gsea.1653623667859/enplot_GOBP_REGULATION_OF_CELLULAR_RESPONSE_TO_HEAT_938.png]

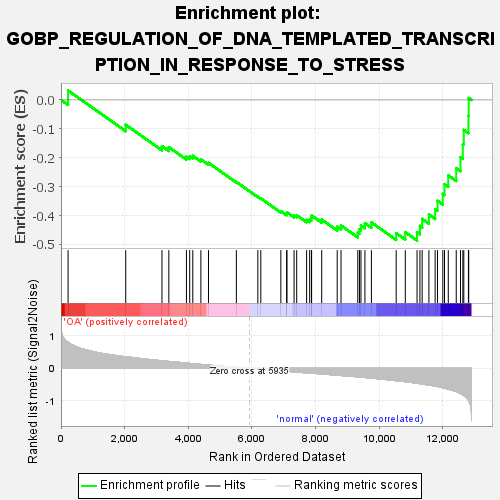

Supplement: Supplementary file 1 — Additional file 1. [file 12891_2023_6585_MOESM1_ESM.zip › BP.Gsea.1653623667859/enplot_GOBP_REGULATION_OF_DNA_TEMPLATED_TRANSCRIPTION_IN_RESPONSE_TO_STRESS_932.png]

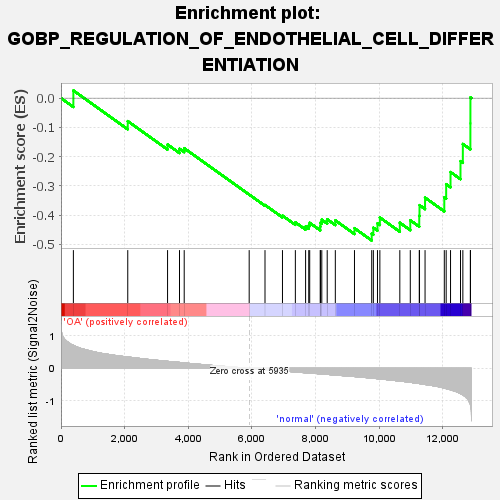

Supplement: Supplementary file 1 — Additional file 1. [file 12891_2023_6585_MOESM1_ESM.zip › BP.Gsea.1653623667859/enplot_GOBP_REGULATION_OF_ENDOTHELIAL_CELL_DIFFERENTIATION_1031.png]

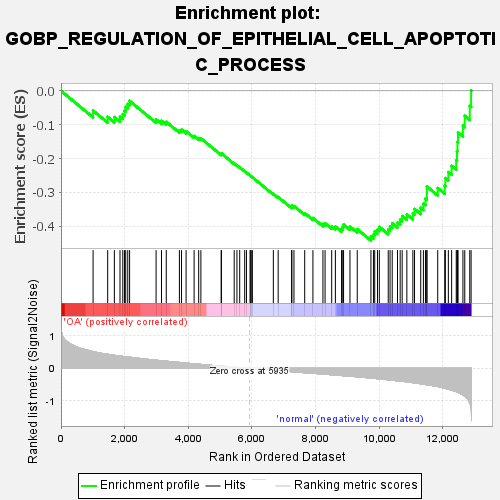

Supplement: Supplementary file 1 — Additional file 1. [file 12891_2023_6585_MOESM1_ESM.zip › BP.Gsea.1653623667859/enplot_GOBP_REGULATION_OF_EPITHELIAL_CELL_APOPTOTIC_PROCESS_929.png]

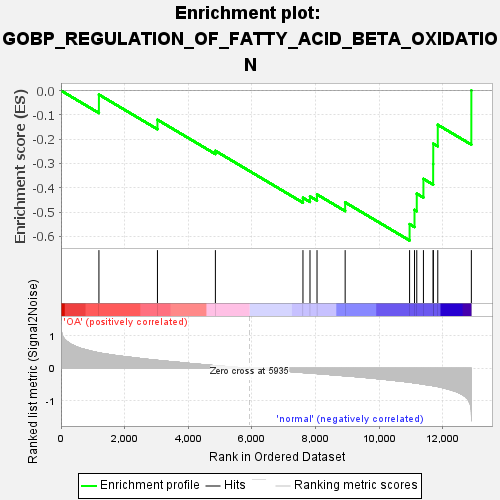

Supplement: Supplementary file 1 — Additional file 1. [file 12891_2023_6585_MOESM1_ESM.zip › BP.Gsea.1653623667859/enplot_GOBP_REGULATION_OF_FATTY_ACID_BETA_OXIDATION_1034.png]

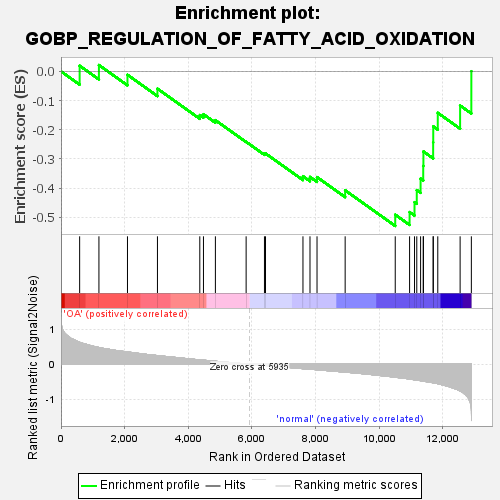

Supplement: Supplementary file 1 — Additional file 1. [file 12891_2023_6585_MOESM1_ESM.zip › BP.Gsea.1653623667859/enplot_GOBP_REGULATION_OF_FATTY_ACID_OXIDATION_986.png]

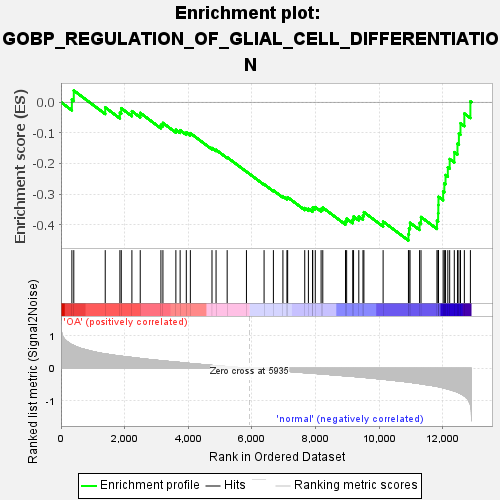

Supplement: Supplementary file 1 — Additional file 1. [file 12891_2023_6585_MOESM1_ESM.zip › BP.Gsea.1653623667859/enplot_GOBP_REGULATION_OF_GLIAL_CELL_DIFFERENTIATION_977.png]

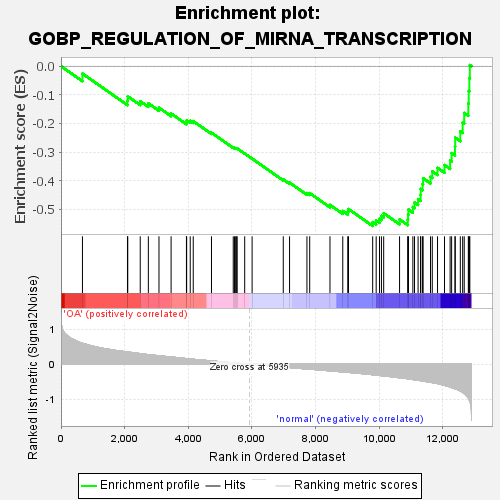

Supplement: Supplementary file 1 — Additional file 1. [file 12891_2023_6585_MOESM1_ESM.zip › BP.Gsea.1653623667859/enplot_GOBP_REGULATION_OF_MIRNA_TRANSCRIPTION_824.png]

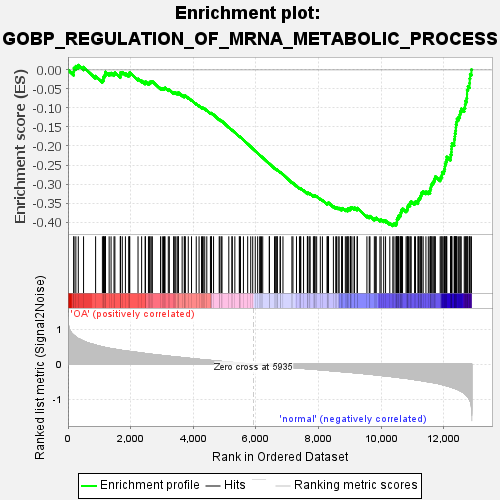

Supplement: Supplementary file 1 — Additional file 1. [file 12891_2023_6585_MOESM1_ESM.zip › BP.Gsea.1653623667859/enplot_GOBP_REGULATION_OF_MRNA_METABOLIC_PROCESS_848.png]

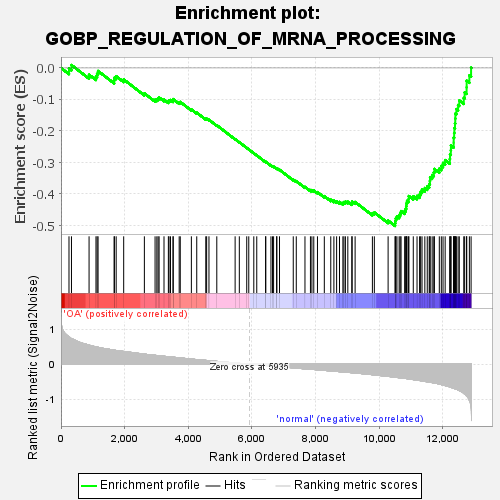

Supplement: Supplementary file 1 — Additional file 1. [file 12891_2023_6585_MOESM1_ESM.zip › BP.Gsea.1653623667859/enplot_GOBP_REGULATION_OF_MRNA_PROCESSING_821.png]

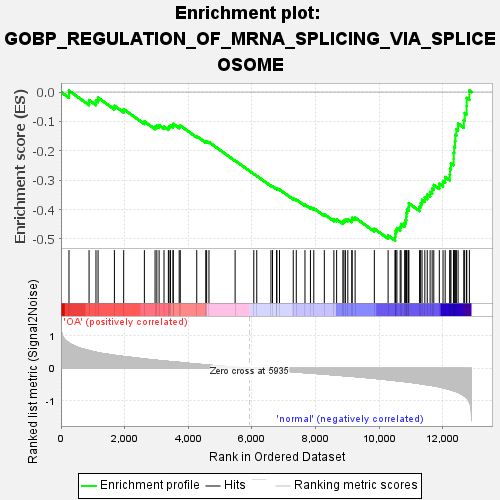

Supplement: Supplementary file 1 — Additional file 1. [file 12891_2023_6585_MOESM1_ESM.zip › BP.Gsea.1653623667859/enplot_GOBP_REGULATION_OF_MRNA_SPLICING_VIA_SPLICEOSOME_827.png]

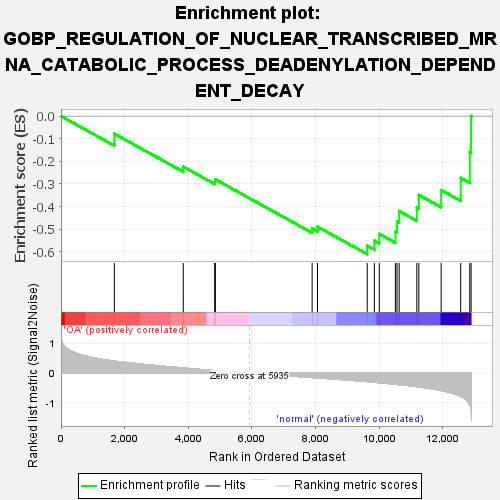

Supplement: Supplementary file 1 — Additional file 1. [file 12891_2023_6585_MOESM1_ESM.zip › BP.Gsea.1653623667859/enplot_GOBP_REGULATION_OF_NUCLEAR_TRANSCRIBED_MRNA_CATABOLIC_PROCESS_DEADENYLATION_DEPENDENT_DECAY_956.png]

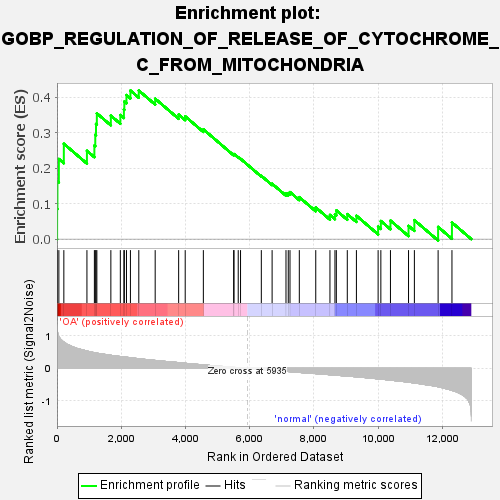

Supplement: Supplementary file 1 — Additional file 1. [file 12891_2023_6585_MOESM1_ESM.zip › BP.Gsea.1653623667859/enplot_GOBP_REGULATION_OF_RELEASE_OF_CYTOCHROME_C_FROM_MITOCHONDRIA_644.png]

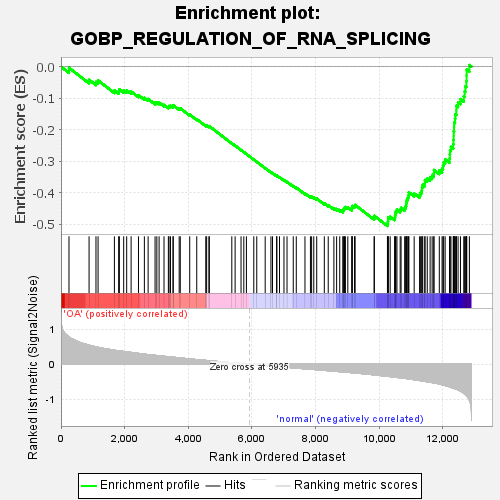

Supplement: Supplementary file 1 — Additional file 1. [file 12891_2023_6585_MOESM1_ESM.zip › BP.Gsea.1653623667859/enplot_GOBP_REGULATION_OF_RNA_SPLICING_815.png]

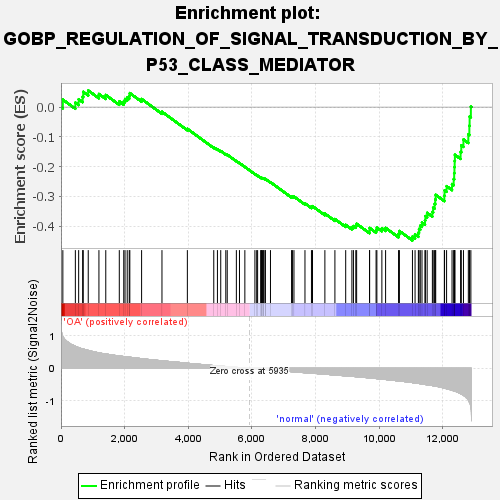

Supplement: Supplementary file 1 — Additional file 1. [file 12891_2023_6585_MOESM1_ESM.zip › BP.Gsea.1653623667859/enplot_GOBP_REGULATION_OF_SIGNAL_TRANSDUCTION_BY_P53_CLASS_MEDIATOR_890.png]

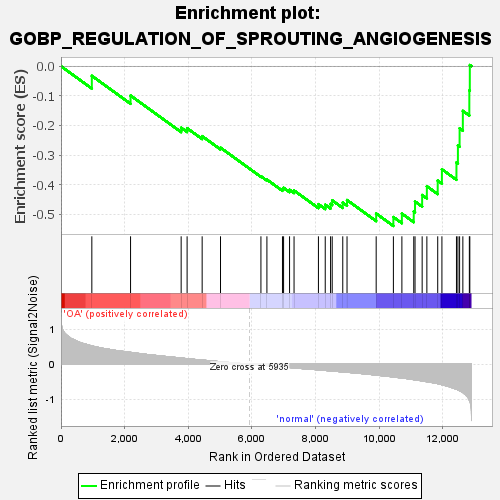

Supplement: Supplementary file 1 — Additional file 1. [file 12891_2023_6585_MOESM1_ESM.zip › BP.Gsea.1653623667859/enplot_GOBP_REGULATION_OF_SPROUTING_ANGIOGENESIS_905.png]

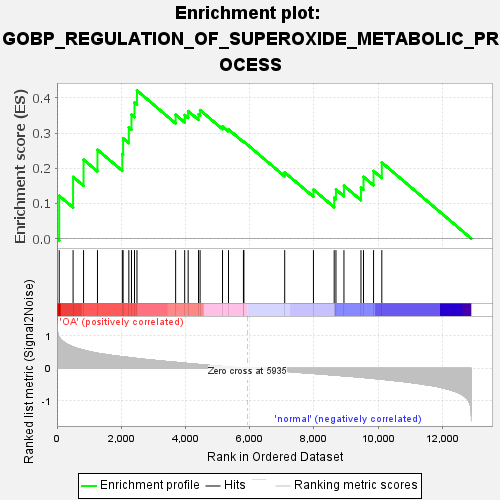

Supplement: Supplementary file 1 — Additional file 1. [file 12891_2023_6585_MOESM1_ESM.zip › BP.Gsea.1653623667859/enplot_GOBP_REGULATION_OF_SUPEROXIDE_METABOLIC_PROCESS_725.png]

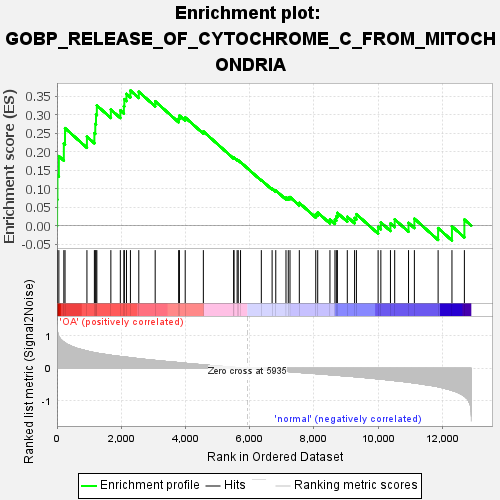

Supplement: Supplementary file 1 — Additional file 1. [file 12891_2023_6585_MOESM1_ESM.zip › BP.Gsea.1653623667859/enplot_GOBP_RELEASE_OF_CYTOCHROME_C_FROM_MITOCHONDRIA_770.png]

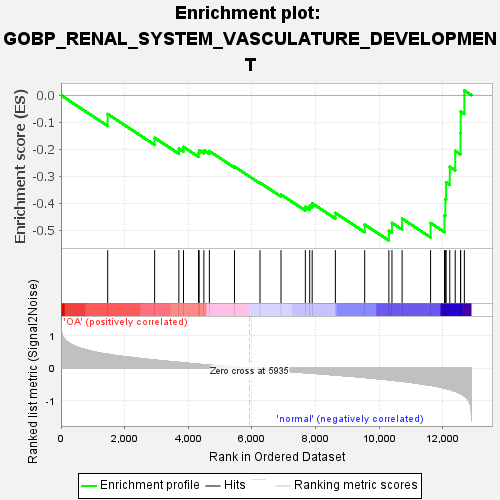

Supplement: Supplementary file 1 — Additional file 1. [file 12891_2023_6585_MOESM1_ESM.zip › BP.Gsea.1653623667859/enplot_GOBP_RENAL_SYSTEM_VASCULATURE_DEVELOPMENT_941.png]

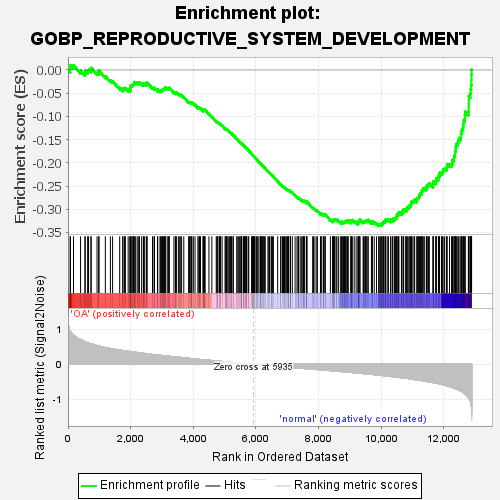

Supplement: Supplementary file 1 — Additional file 1. [file 12891_2023_6585_MOESM1_ESM.zip › BP.Gsea.1653623667859/enplot_GOBP_REPRODUCTIVE_SYSTEM_DEVELOPMENT_1043.png]

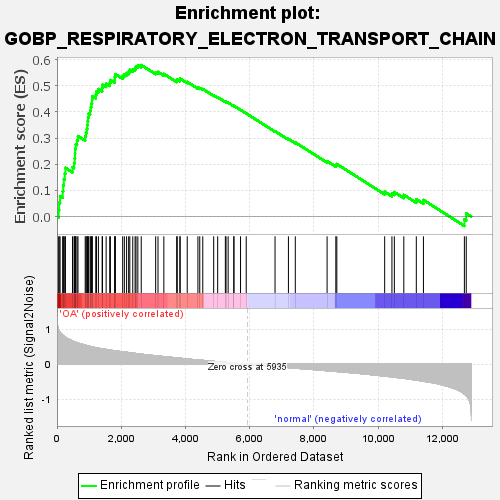

Supplement: Supplementary file 1 — Additional file 1. [file 12891_2023_6585_MOESM1_ESM.zip › BP.Gsea.1653623667859/enplot_GOBP_RESPIRATORY_ELECTRON_TRANSPORT_CHAIN_530.png]

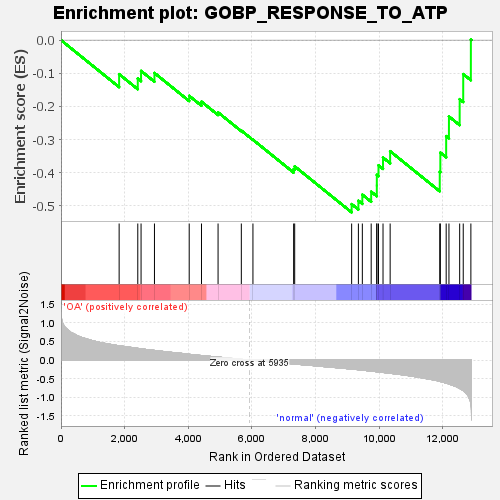

Supplement: Supplementary file 1 — Additional file 1. [file 12891_2023_6585_MOESM1_ESM.zip › BP.Gsea.1653623667859/enplot_GOBP_RESPONSE_TO_ATP_1052.png]

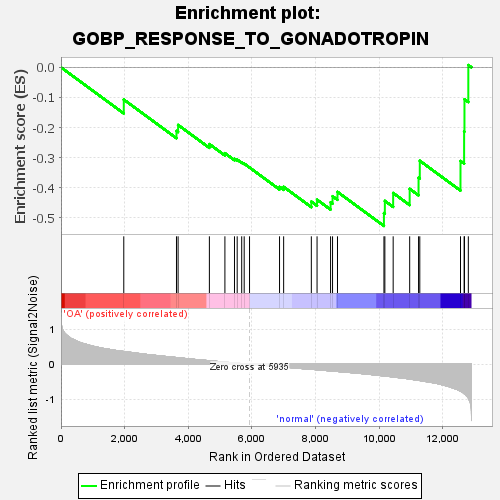

Supplement: Supplementary file 1 — Additional file 1. [file 12891_2023_6585_MOESM1_ESM.zip › BP.Gsea.1653623667859/enplot_GOBP_RESPONSE_TO_GONADOTROPIN_968.png]

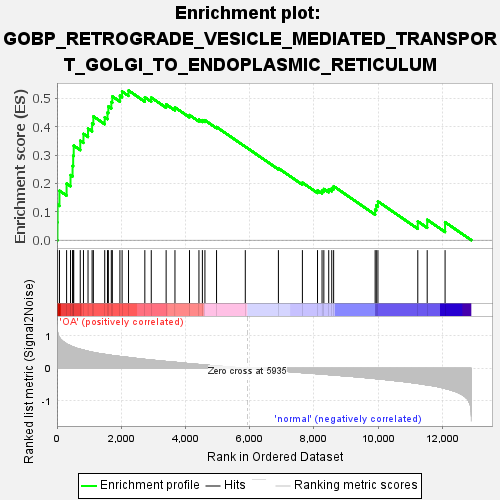

Supplement: Supplementary file 1 — Additional file 1. [file 12891_2023_6585_MOESM1_ESM.zip › BP.Gsea.1653623667859/enplot_GOBP_RETROGRADE_VESICLE_MEDIATED_TRANSPORT_GOLGI_TO_ENDOPLASMIC_RETICULUM_548.png]

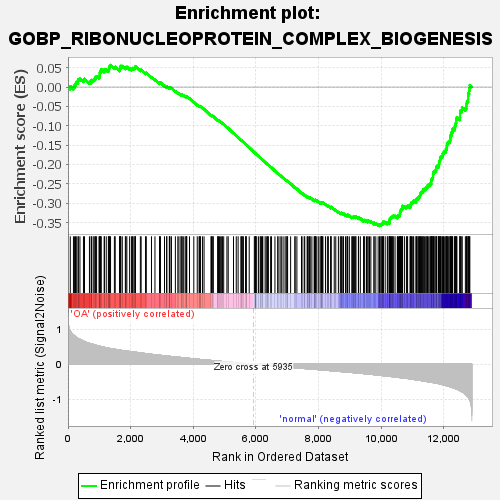

Supplement: Supplementary file 1 — Additional file 1. [file 12891_2023_6585_MOESM1_ESM.zip › BP.Gsea.1653623667859/enplot_GOBP_RIBONUCLEOPROTEIN_COMPLEX_BIOGENESIS_947.png]

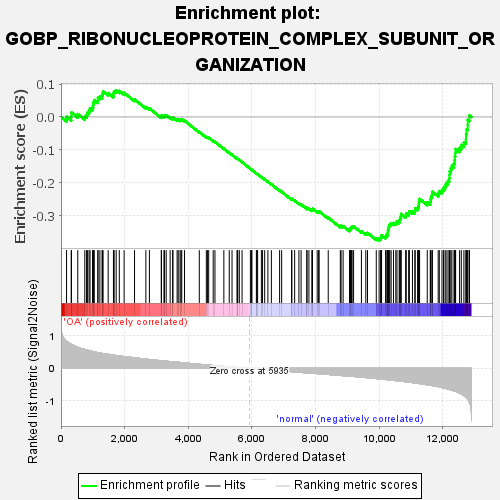

Supplement: Supplementary file 1 — Additional file 1. [file 12891_2023_6585_MOESM1_ESM.zip › BP.Gsea.1653623667859/enplot_GOBP_RIBONUCLEOPROTEIN_COMPLEX_SUBUNIT_ORGANIZATION_1028.png]

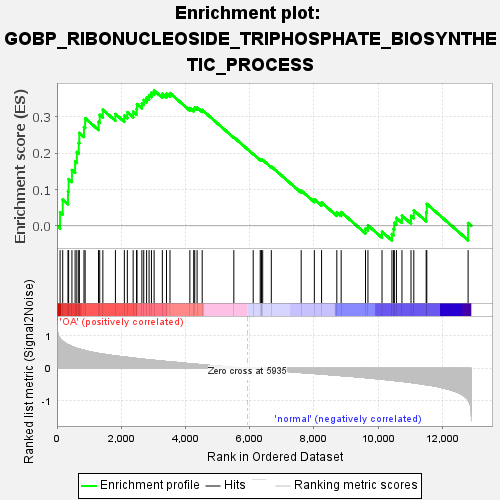

Supplement: Supplementary file 1 — Additional file 1. [file 12891_2023_6585_MOESM1_ESM.zip › BP.Gsea.1653623667859/enplot_GOBP_RIBONUCLEOSIDE_TRIPHOSPHATE_BIOSYNTHETIC_PROCESS_698.png]

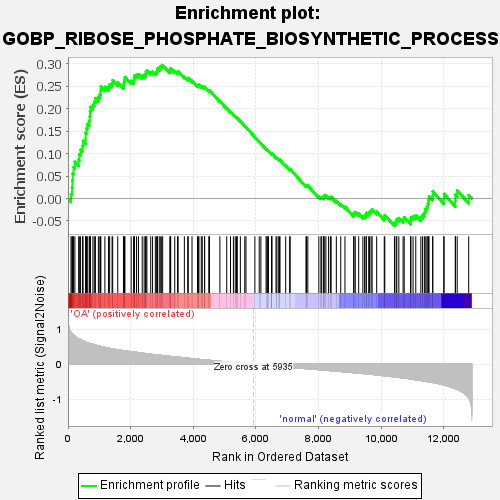

Supplement: Supplementary file 1 — Additional file 1. [file 12891_2023_6585_MOESM1_ESM.zip › BP.Gsea.1653623667859/enplot_GOBP_RIBOSE_PHOSPHATE_BIOSYNTHETIC_PROCESS_752.png]

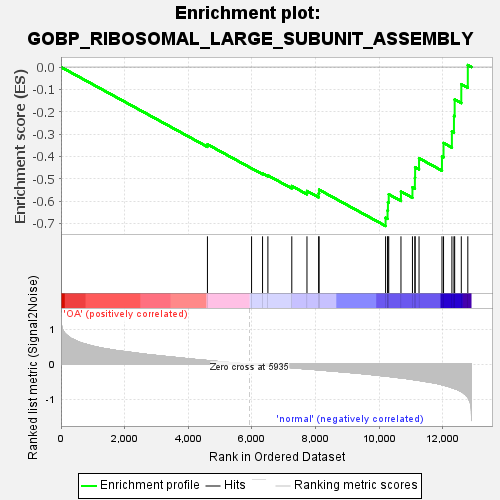

Supplement: Supplementary file 1 — Additional file 1. [file 12891_2023_6585_MOESM1_ESM.zip › BP.Gsea.1653623667859/enplot_GOBP_RIBOSOMAL_LARGE_SUBUNIT_ASSEMBLY_818.png]

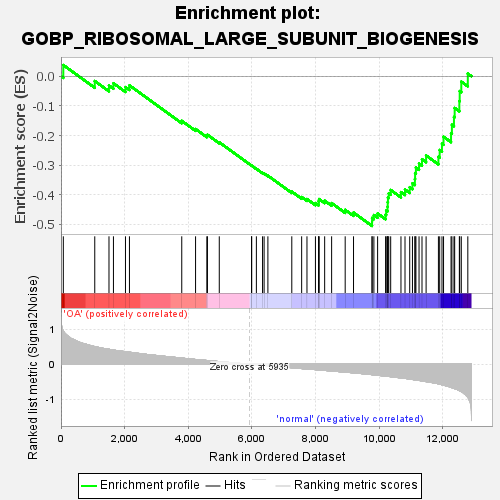

Supplement: Supplementary file 1 — Additional file 1. [file 12891_2023_6585_MOESM1_ESM.zip › BP.Gsea.1653623667859/enplot_GOBP_RIBOSOMAL_LARGE_SUBUNIT_BIOGENESIS_842.png]

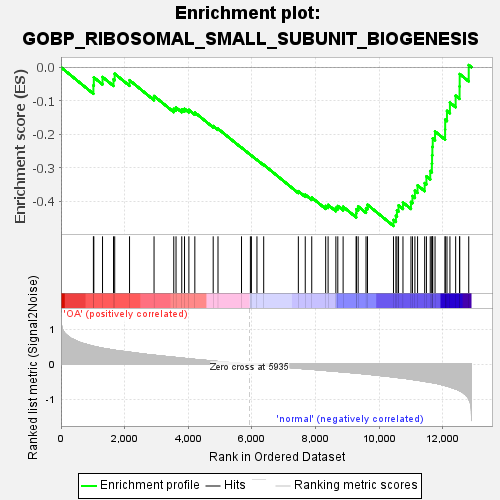

Supplement: Supplementary file 1 — Additional file 1. [file 12891_2023_6585_MOESM1_ESM.zip › BP.Gsea.1653623667859/enplot_GOBP_RIBOSOMAL_SMALL_SUBUNIT_BIOGENESIS_917.png]

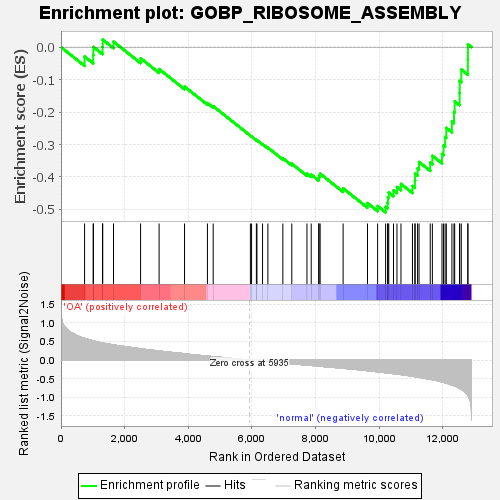

Supplement: Supplementary file 1 — Additional file 1. [file 12891_2023_6585_MOESM1_ESM.zip › BP.Gsea.1653623667859/enplot_GOBP_RIBOSOME_ASSEMBLY_872.png]

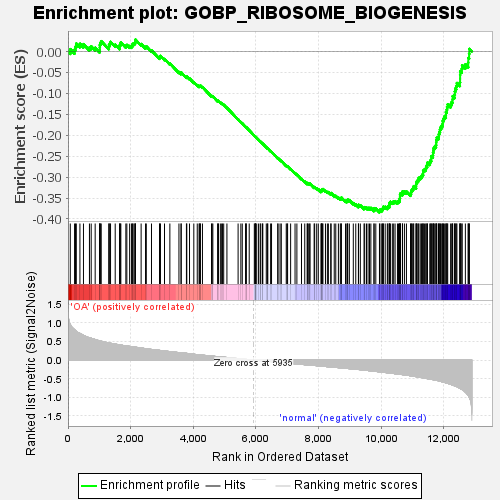

Supplement: Supplementary file 1 — Additional file 1. [file 12891_2023_6585_MOESM1_ESM.zip › BP.Gsea.1653623667859/enplot_GOBP_RIBOSOME_BIOGENESIS_902.png]

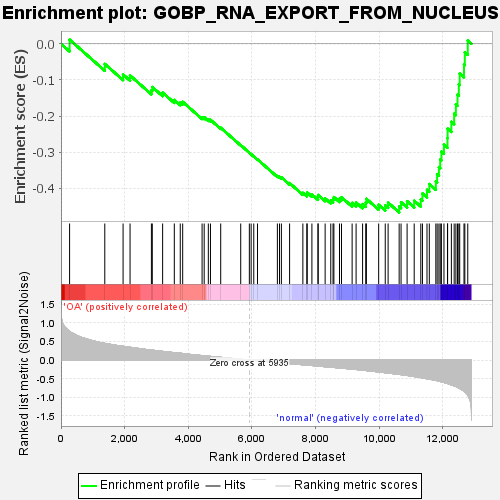

Supplement: Supplementary file 1 — Additional file 1. [file 12891_2023_6585_MOESM1_ESM.zip › BP.Gsea.1653623667859/enplot_GOBP_RNA_EXPORT_FROM_NUCLEUS_878.png]

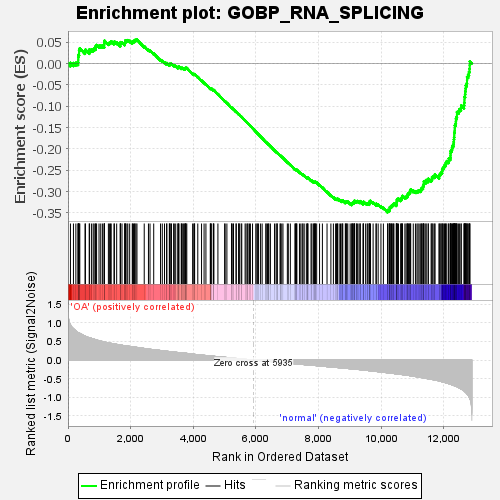

Supplement: Supplementary file 1 — Additional file 1. [file 12891_2023_6585_MOESM1_ESM.zip › BP.Gsea.1653623667859/enplot_GOBP_RNA_SPLICING_980.png]

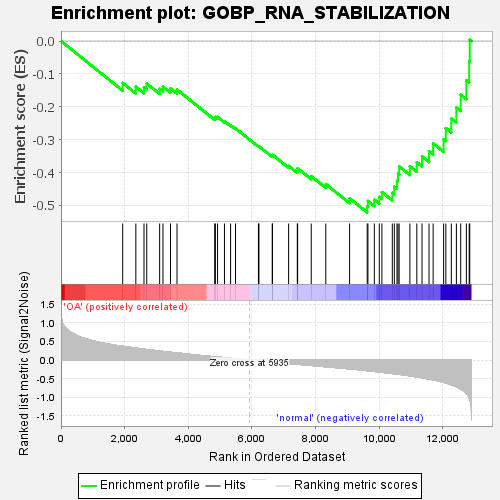

Supplement: Supplementary file 1 — Additional file 1. [file 12891_2023_6585_MOESM1_ESM.zip › BP.Gsea.1653623667859/enplot_GOBP_RNA_STABILIZATION_863.png]

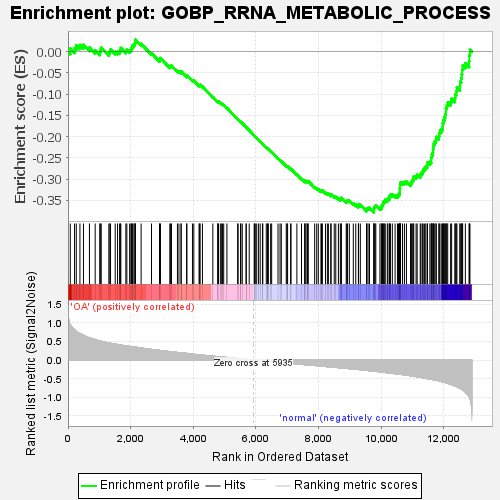

Supplement: Supplementary file 1 — Additional file 1. [file 12891_2023_6585_MOESM1_ESM.zip › BP.Gsea.1653623667859/enplot_GOBP_RRNA_METABOLIC_PROCESS_950.png]

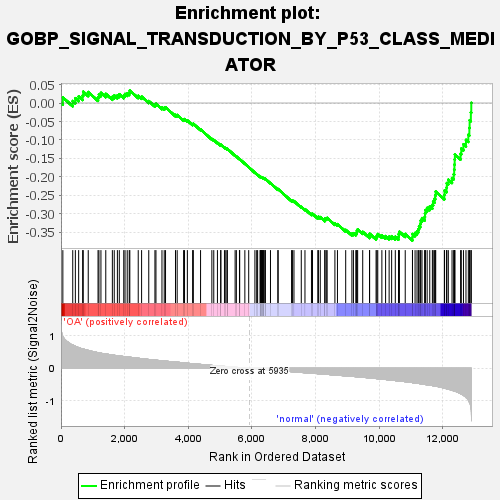

Supplement: Supplementary file 1 — Additional file 1. [file 12891_2023_6585_MOESM1_ESM.zip › BP.Gsea.1653623667859/enplot_GOBP_SIGNAL_TRANSDUCTION_BY_P53_CLASS_MEDIATOR_1103.png]

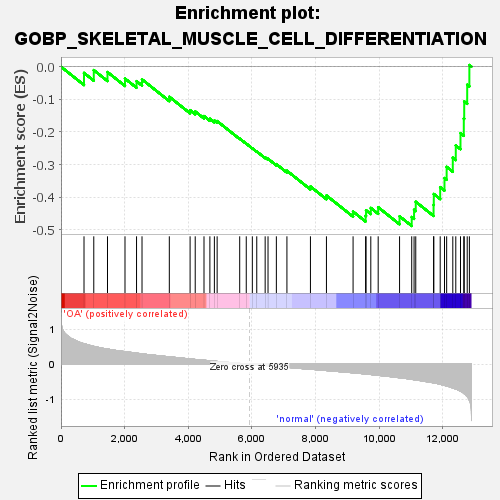

Supplement: Supplementary file 1 — Additional file 1. [file 12891_2023_6585_MOESM1_ESM.zip › BP.Gsea.1653623667859/enplot_GOBP_SKELETAL_MUSCLE_CELL_DIFFERENTIATION_959.png]

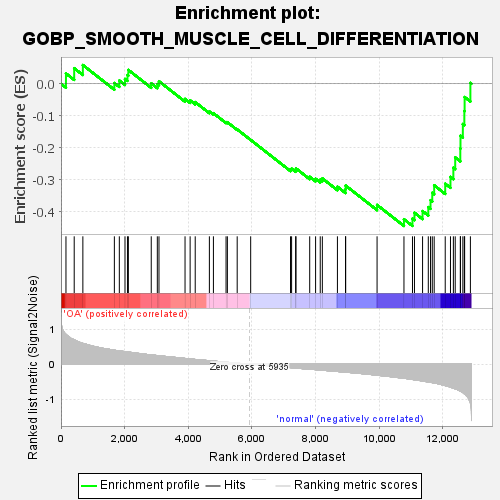

Supplement: Supplementary file 1 — Additional file 1. [file 12891_2023_6585_MOESM1_ESM.zip › BP.Gsea.1653623667859/enplot_GOBP_SMOOTH_MUSCLE_CELL_DIFFERENTIATION_1106.png]

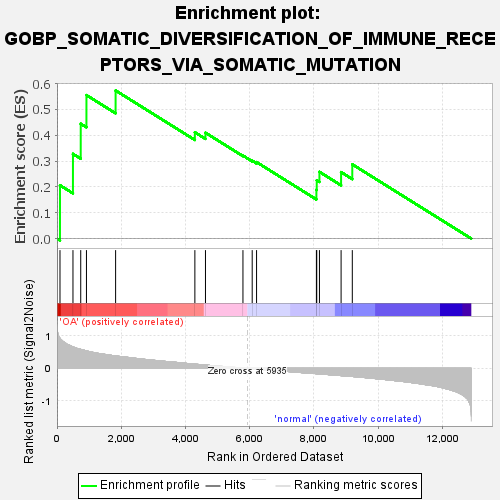

Supplement: Supplementary file 1 — Additional file 1. [file 12891_2023_6585_MOESM1_ESM.zip › BP.Gsea.1653623667859/enplot_GOBP_SOMATIC_DIVERSIFICATION_OF_IMMUNE_RECEPTORS_VIA_SOMATIC_MUTATION_629.png]

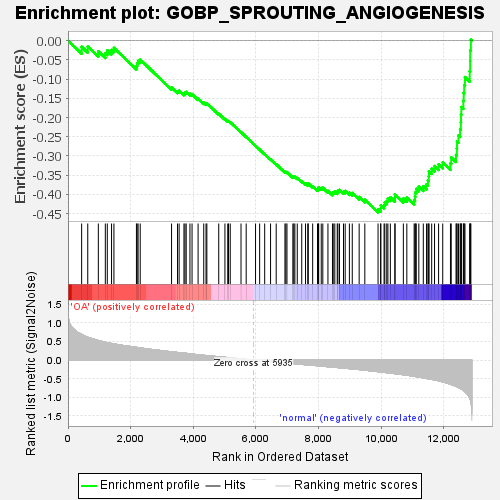

Supplement: Supplementary file 1 — Additional file 1. [file 12891_2023_6585_MOESM1_ESM.zip › BP.Gsea.1653623667859/enplot_GOBP_SPROUTING_ANGIOGENESIS_857.png]

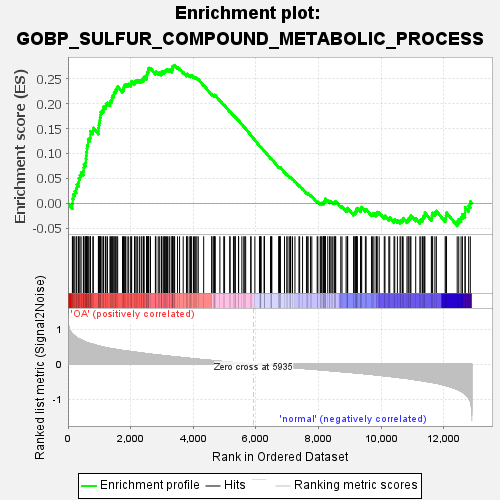

Supplement: Supplementary file 1 — Additional file 1. [file 12891_2023_6585_MOESM1_ESM.zip › BP.Gsea.1653623667859/enplot_GOBP_SULFUR_COMPOUND_METABOLIC_PROCESS_758.png]

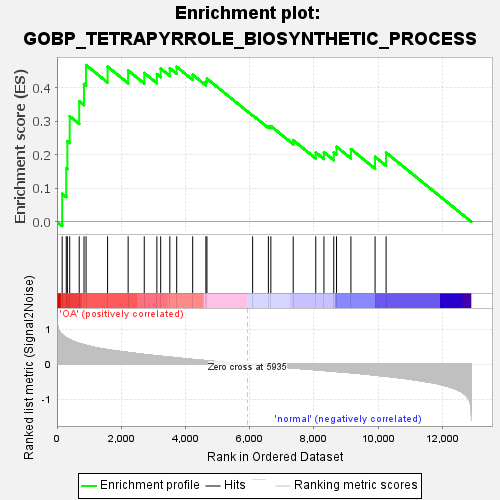

Supplement: Supplementary file 1 — Additional file 1. [file 12891_2023_6585_MOESM1_ESM.zip › BP.Gsea.1653623667859/enplot_GOBP_TETRAPYRROLE_BIOSYNTHETIC_PROCESS_641.png]
