# Supplementary figures and images for: Pyruvate Dehydrogenase Kinase 1 inhibition mediated oxidative phosphorylation enhancement in cartilage promotes osteoarthritis progression (part 1 of 2)
Source: BMC Musculoskelet Disord. 2023 Jul 20;24:597. doi: 10.1186/s12891-023-06585-6 (PMC10357736; doi:10.1186/s12891-023-06585-6)

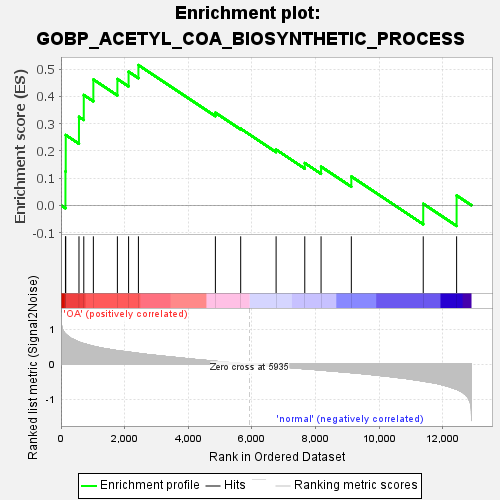

Supplement: Supplementary file 1 — Additional file 1. [file 12891_2023_6585_MOESM1_ESM.zip › BP.Gsea.1653623667859/enplot_GOBP_ACETYL_COA_BIOSYNTHETIC_PROCESS_683.png]

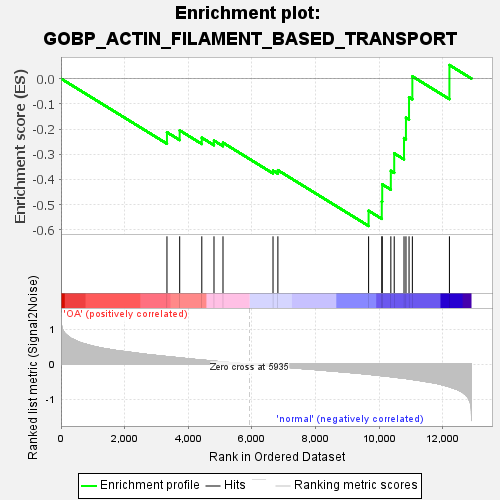

Supplement: Supplementary file 1 — Additional file 1. [file 12891_2023_6585_MOESM1_ESM.zip › BP.Gsea.1653623667859/enplot_GOBP_ACTIN_FILAMENT_BASED_TRANSPORT_1061.png]

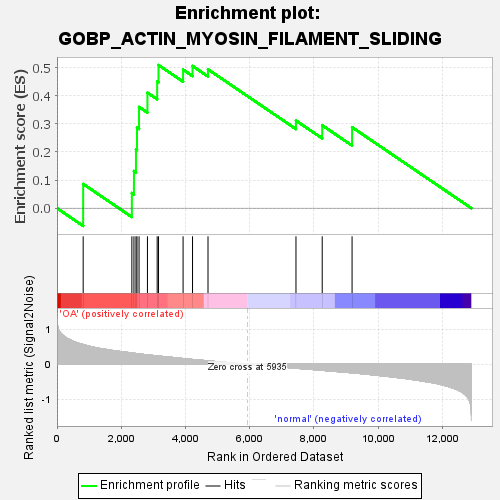

Supplement: Supplementary file 1 — Additional file 1. [file 12891_2023_6585_MOESM1_ESM.zip › BP.Gsea.1653623667859/enplot_GOBP_ACTIN_MYOSIN_FILAMENT_SLIDING_722.png]

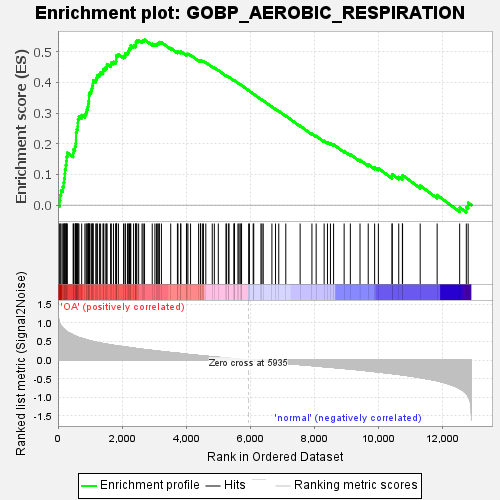

Supplement: Supplementary file 1 — Additional file 1. [file 12891_2023_6585_MOESM1_ESM.zip › BP.Gsea.1653623667859/enplot_GOBP_AEROBIC_RESPIRATION_527.png]

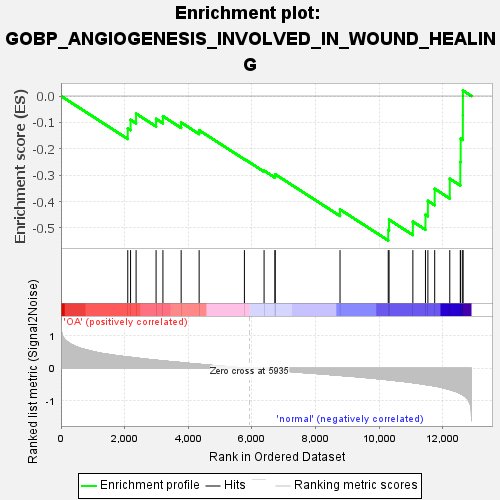

Supplement: Supplementary file 1 — Additional file 1. [file 12891_2023_6585_MOESM1_ESM.zip › BP.Gsea.1653623667859/enplot_GOBP_ANGIOGENESIS_INVOLVED_IN_WOUND_HEALING_1022.png]

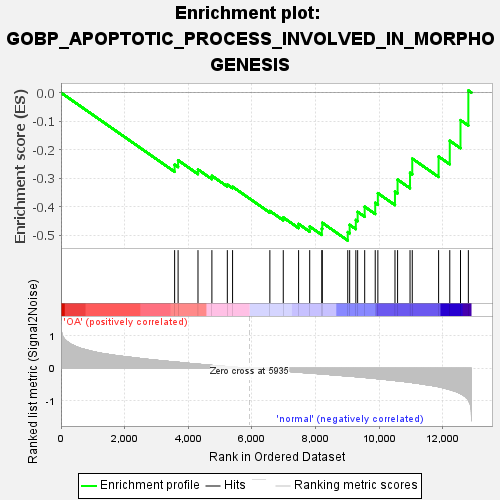

Supplement: Supplementary file 1 — Additional file 1. [file 12891_2023_6585_MOESM1_ESM.zip › BP.Gsea.1653623667859/enplot_GOBP_APOPTOTIC_PROCESS_INVOLVED_IN_MORPHOGENESIS_1058.png]

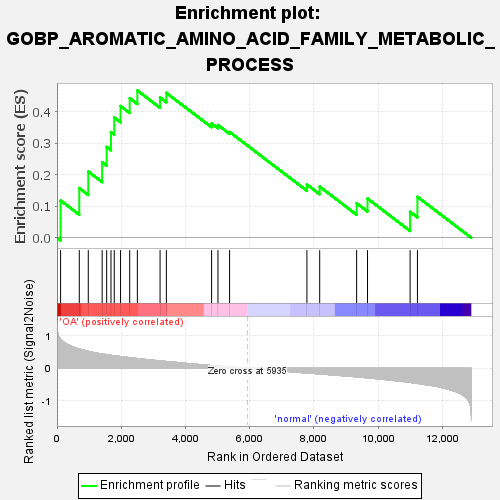

Supplement: Supplementary file 1 — Additional file 1. [file 12891_2023_6585_MOESM1_ESM.zip › BP.Gsea.1653623667859/enplot_GOBP_AROMATIC_AMINO_ACID_FAMILY_METABOLIC_PROCESS_707.png]

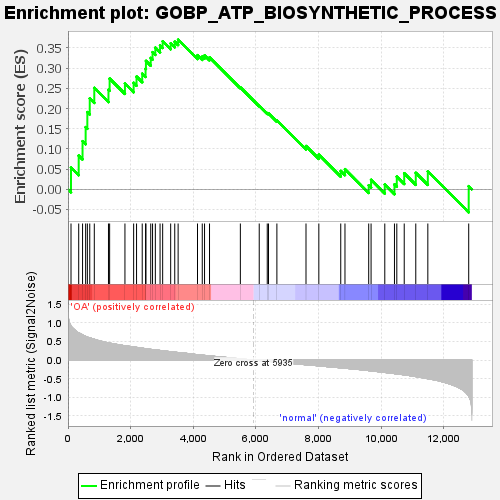

Supplement: Supplementary file 1 — Additional file 1. [file 12891_2023_6585_MOESM1_ESM.zip › BP.Gsea.1653623667859/enplot_GOBP_ATP_BIOSYNTHETIC_PROCESS_749.png]

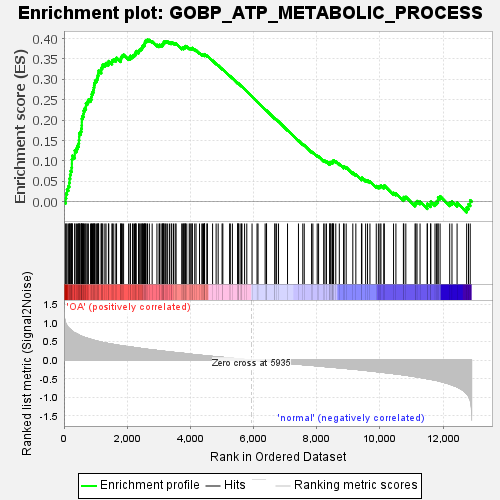

Supplement: Supplementary file 1 — Additional file 1. [file 12891_2023_6585_MOESM1_ESM.zip › BP.Gsea.1653623667859/enplot_GOBP_ATP_METABOLIC_PROCESS_551.png]

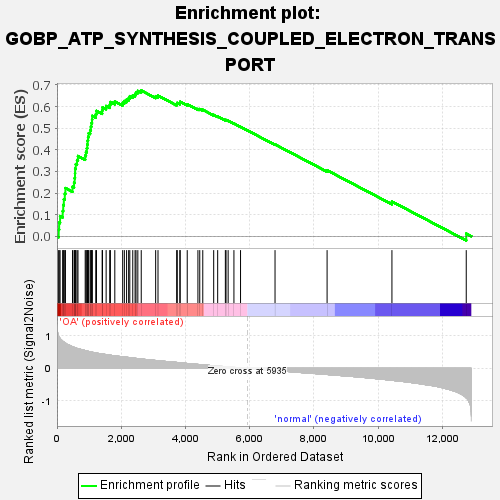

Supplement: Supplementary file 1 — Additional file 1. [file 12891_2023_6585_MOESM1_ESM.zip › BP.Gsea.1653623667859/enplot_GOBP_ATP_SYNTHESIS_COUPLED_ELECTRON_TRANSPORT_512.png]

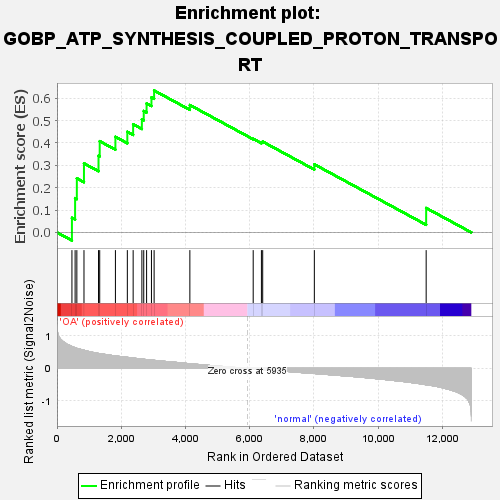

Supplement: Supplementary file 1 — Additional file 1. [file 12891_2023_6585_MOESM1_ESM.zip › BP.Gsea.1653623667859/enplot_GOBP_ATP_SYNTHESIS_COUPLED_PROTON_TRANSPORT_557.png]

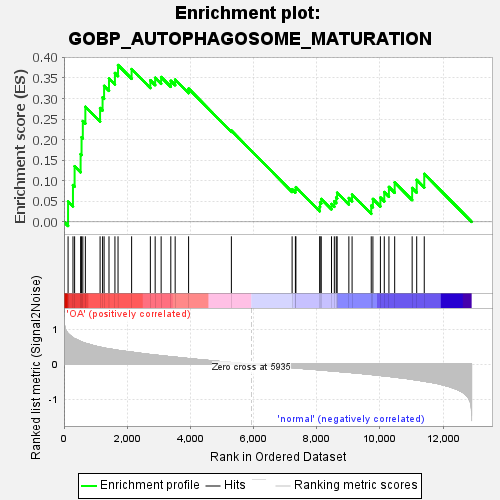

Supplement: Supplementary file 1 — Additional file 1. [file 12891_2023_6585_MOESM1_ESM.zip › BP.Gsea.1653623667859/enplot_GOBP_AUTOPHAGOSOME_MATURATION_776.png]

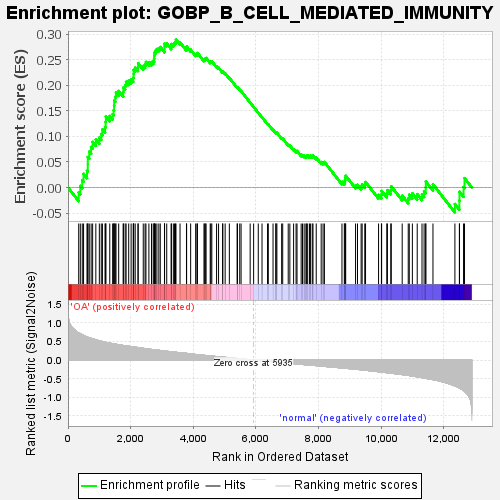

Supplement: Supplementary file 1 — Additional file 1. [file 12891_2023_6585_MOESM1_ESM.zip › BP.Gsea.1653623667859/enplot_GOBP_B_CELL_MEDIATED_IMMUNITY_791.png]

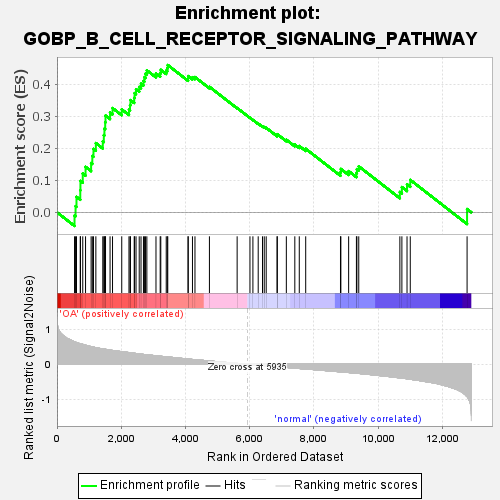

Supplement: Supplementary file 1 — Additional file 1. [file 12891_2023_6585_MOESM1_ESM.zip › BP.Gsea.1653623667859/enplot_GOBP_B_CELL_RECEPTOR_SIGNALING_PATHWAY_554.png]

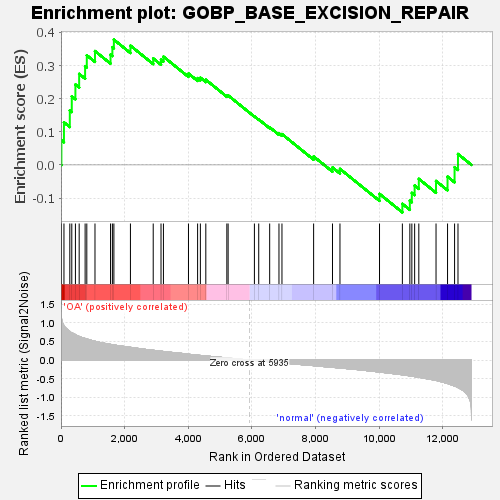

Supplement: Supplementary file 1 — Additional file 1. [file 12891_2023_6585_MOESM1_ESM.zip › BP.Gsea.1653623667859/enplot_GOBP_BASE_EXCISION_REPAIR_779.png]

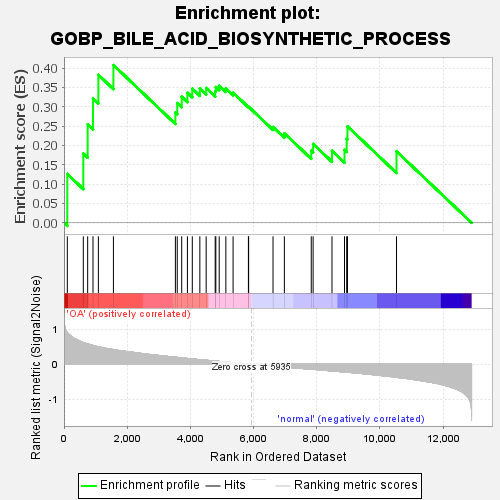

Supplement: Supplementary file 1 — Additional file 1. [file 12891_2023_6585_MOESM1_ESM.zip › BP.Gsea.1653623667859/enplot_GOBP_BILE_ACID_BIOSYNTHETIC_PROCESS_734.png]

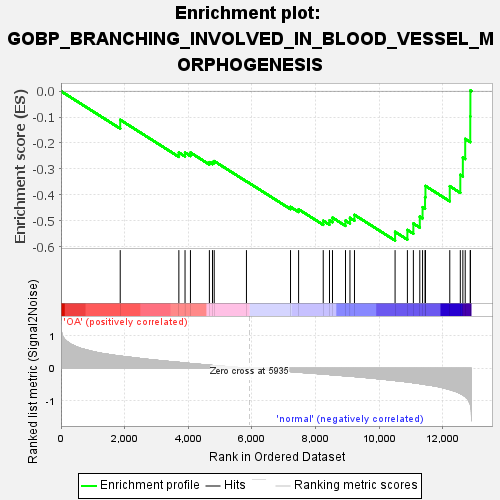

Supplement: Supplementary file 1 — Additional file 1. [file 12891_2023_6585_MOESM1_ESM.zip › BP.Gsea.1653623667859/enplot_GOBP_BRANCHING_INVOLVED_IN_BLOOD_VESSEL_MORPHOGENESIS_875.png]

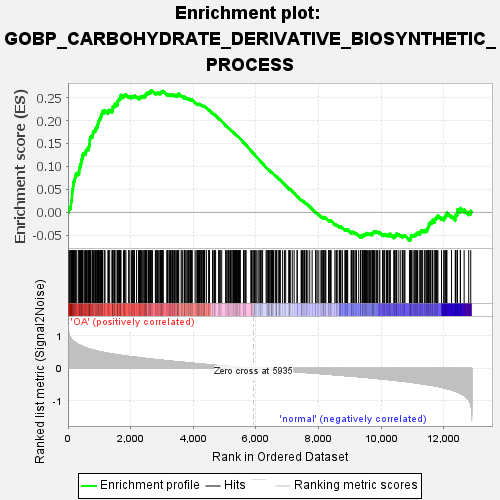

Supplement: Supplementary file 1 — Additional file 1. [file 12891_2023_6585_MOESM1_ESM.zip › BP.Gsea.1653623667859/enplot_GOBP_CARBOHYDRATE_DERIVATIVE_BIOSYNTHETIC_PROCESS_728.png]

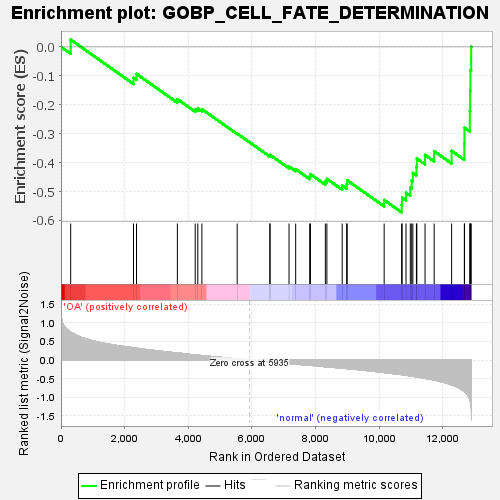

Supplement: Supplementary file 1 — Additional file 1. [file 12891_2023_6585_MOESM1_ESM.zip › BP.Gsea.1653623667859/enplot_GOBP_CELL_FATE_DETERMINATION_833.png]

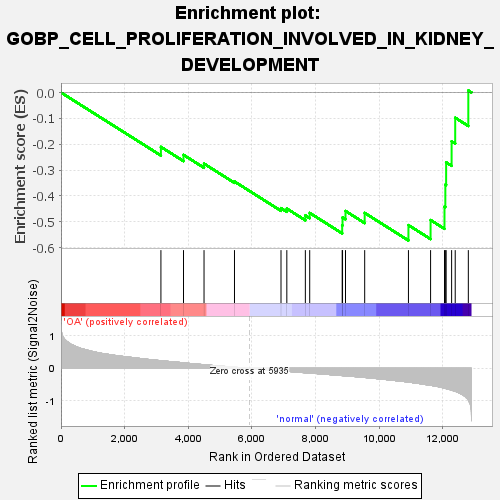

Supplement: Supplementary file 1 — Additional file 1. [file 12891_2023_6585_MOESM1_ESM.zip › BP.Gsea.1653623667859/enplot_GOBP_CELL_PROLIFERATION_INVOLVED_IN_KIDNEY_DEVELOPMENT_1010.png]

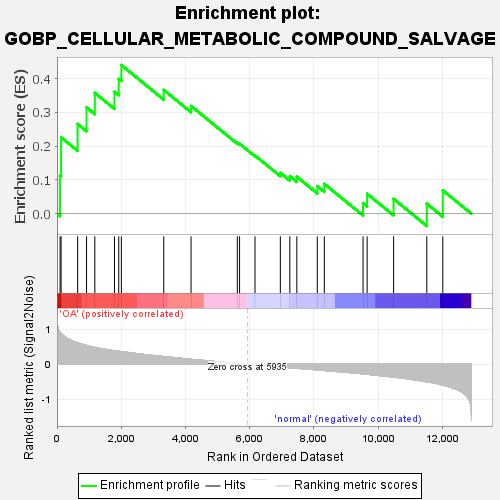

Supplement: Supplementary file 1 — Additional file 1. [file 12891_2023_6585_MOESM1_ESM.zip › BP.Gsea.1653623667859/enplot_GOBP_CELLULAR_METABOLIC_COMPOUND_SALVAGE_755.png]

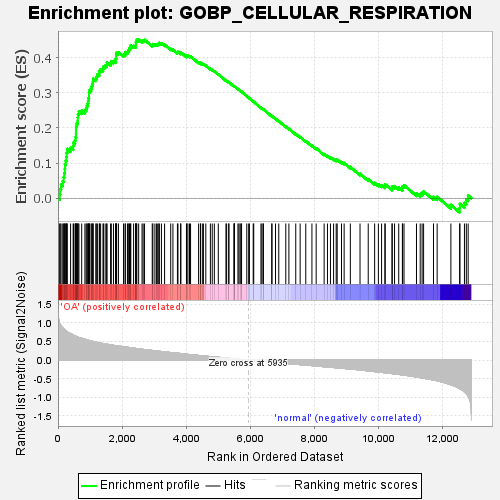

Supplement: Supplementary file 1 — Additional file 1. [file 12891_2023_6585_MOESM1_ESM.zip › BP.Gsea.1653623667859/enplot_GOBP_CELLULAR_RESPIRATION_536.png]

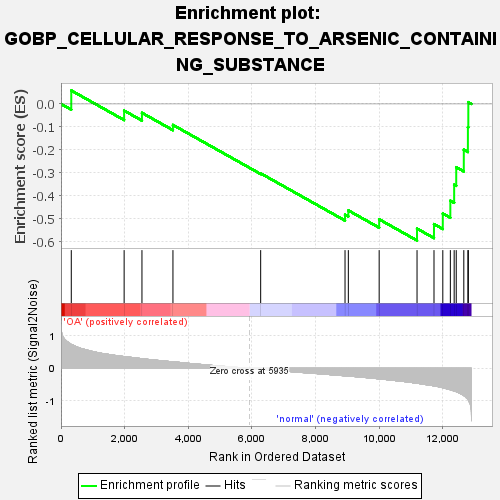

Supplement: Supplementary file 1 — Additional file 1. [file 12891_2023_6585_MOESM1_ESM.zip › BP.Gsea.1653623667859/enplot_GOBP_CELLULAR_RESPONSE_TO_ARSENIC_CONTAINING_SUBSTANCE_1073.png]

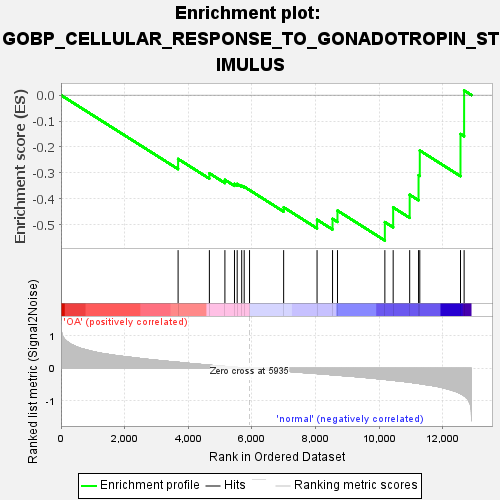

Supplement: Supplementary file 1 — Additional file 1. [file 12891_2023_6585_MOESM1_ESM.zip › BP.Gsea.1653623667859/enplot_GOBP_CELLULAR_RESPONSE_TO_GONADOTROPIN_STIMULUS_1067.png]

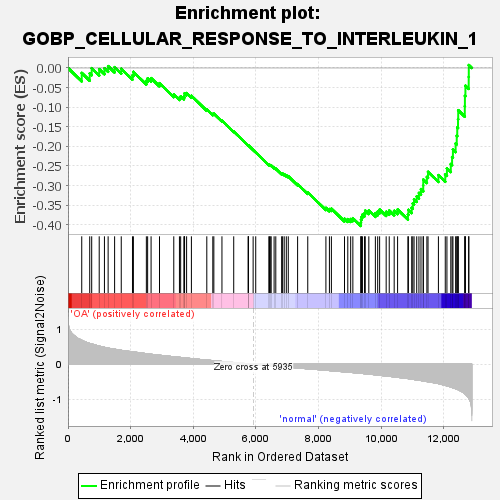

Supplement: Supplementary file 1 — Additional file 1. [file 12891_2023_6585_MOESM1_ESM.zip › BP.Gsea.1653623667859/enplot_GOBP_CELLULAR_RESPONSE_TO_INTERLEUKIN_1_1046.png]

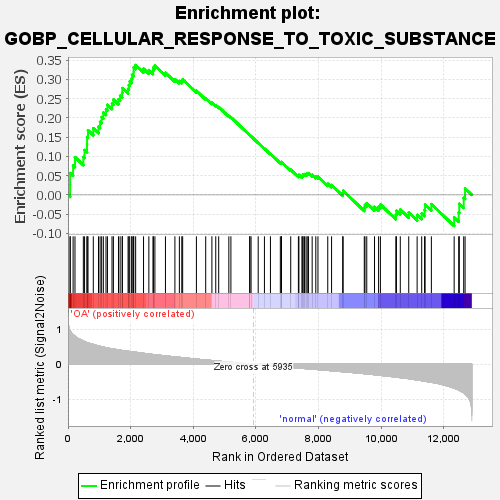

Supplement: Supplementary file 1 — Additional file 1. [file 12891_2023_6585_MOESM1_ESM.zip › BP.Gsea.1653623667859/enplot_GOBP_CELLULAR_RESPONSE_TO_TOXIC_SUBSTANCE_716.png]

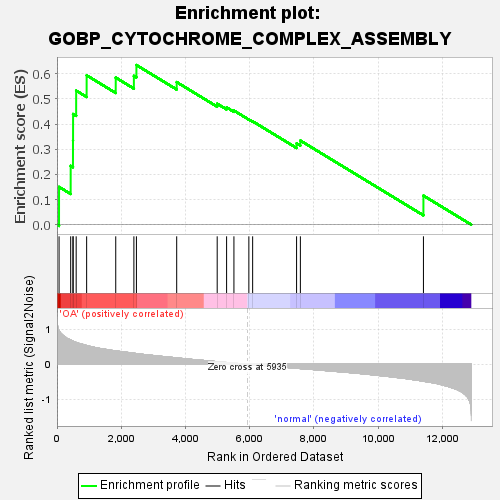

Supplement: Supplementary file 1 — Additional file 1. [file 12891_2023_6585_MOESM1_ESM.zip › BP.Gsea.1653623667859/enplot_GOBP_CYTOCHROME_COMPLEX_ASSEMBLY_560.png]

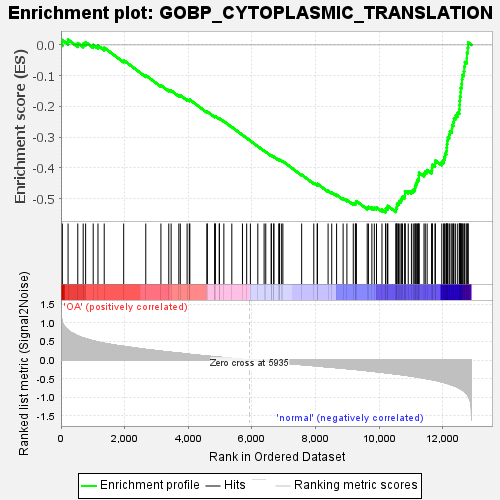

Supplement: Supplementary file 1 — Additional file 1. [file 12891_2023_6585_MOESM1_ESM.zip › BP.Gsea.1653623667859/enplot_GOBP_CYTOPLASMIC_TRANSLATION_812.png]

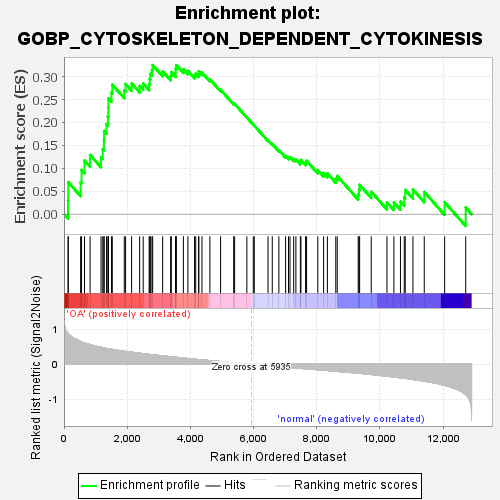

Supplement: Supplementary file 1 — Additional file 1. [file 12891_2023_6585_MOESM1_ESM.zip › BP.Gsea.1653623667859/enplot_GOBP_CYTOSKELETON_DEPENDENT_CYTOKINESIS_797.png]

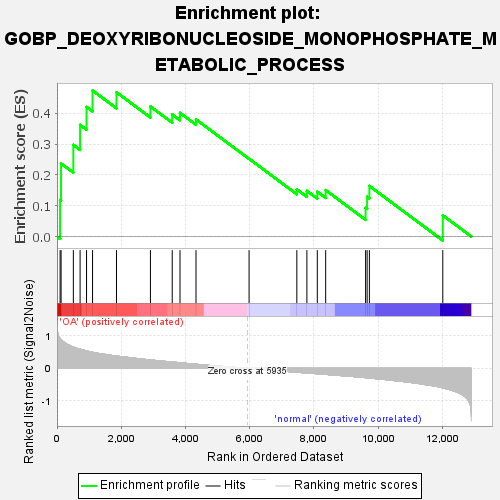

Supplement: Supplementary file 1 — Additional file 1. [file 12891_2023_6585_MOESM1_ESM.zip › BP.Gsea.1653623667859/enplot_GOBP_DEOXYRIBONUCLEOSIDE_MONOPHOSPHATE_METABOLIC_PROCESS_740.png]

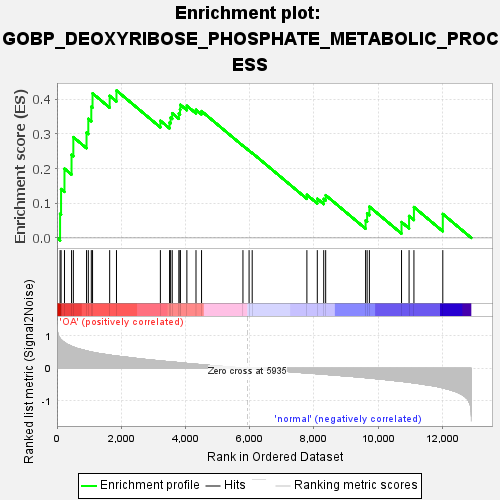

Supplement: Supplementary file 1 — Additional file 1. [file 12891_2023_6585_MOESM1_ESM.zip › BP.Gsea.1653623667859/enplot_GOBP_DEOXYRIBOSE_PHOSPHATE_METABOLIC_PROCESS_665.png]

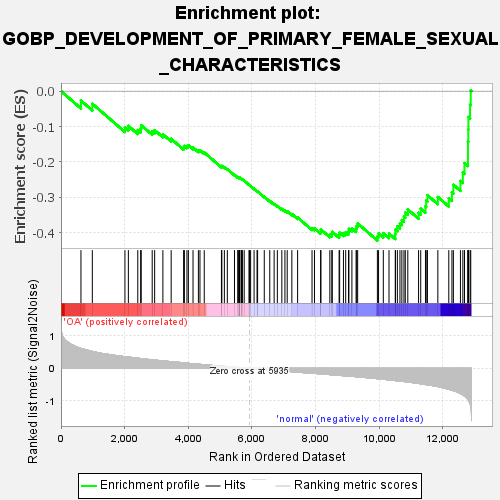

Supplement: Supplementary file 1 — Additional file 1. [file 12891_2023_6585_MOESM1_ESM.zip › BP.Gsea.1653623667859/enplot_GOBP_DEVELOPMENT_OF_PRIMARY_FEMALE_SEXUAL_CHARACTERISTICS_944.png]

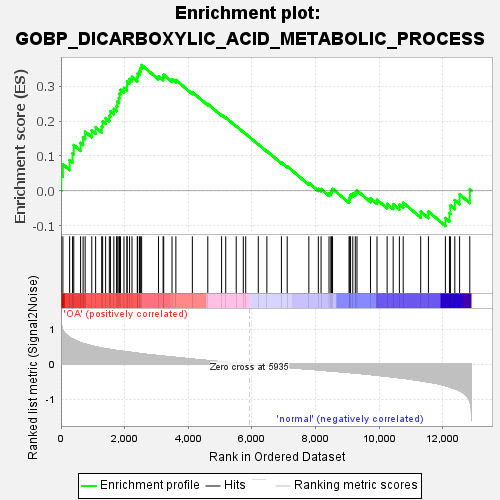

Supplement: Supplementary file 1 — Additional file 1. [file 12891_2023_6585_MOESM1_ESM.zip › BP.Gsea.1653623667859/enplot_GOBP_DICARBOXYLIC_ACID_METABOLIC_PROCESS_680.png]

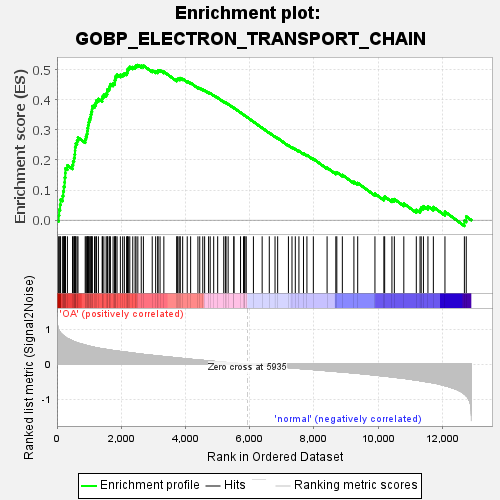

Supplement: Supplementary file 1 — Additional file 1. [file 12891_2023_6585_MOESM1_ESM.zip › BP.Gsea.1653623667859/enplot_GOBP_ELECTRON_TRANSPORT_CHAIN_533.png]

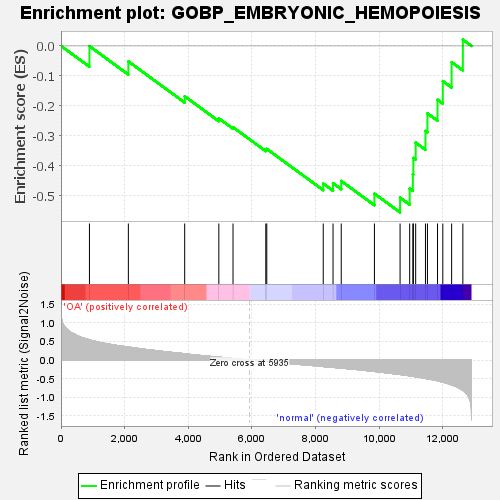

Supplement: Supplementary file 1 — Additional file 1. [file 12891_2023_6585_MOESM1_ESM.zip › BP.Gsea.1653623667859/enplot_GOBP_EMBRYONIC_HEMOPOIESIS_1013.png]

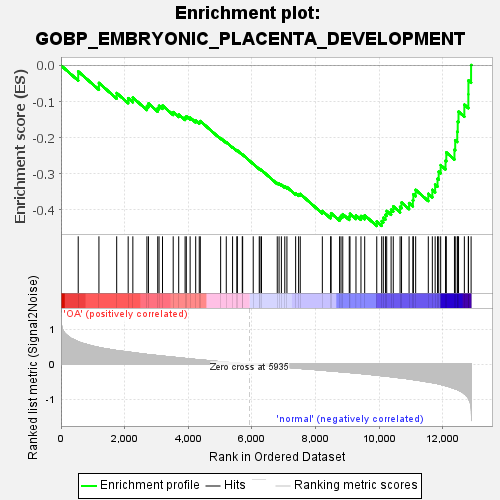

Supplement: Supplementary file 1 — Additional file 1. [file 12891_2023_6585_MOESM1_ESM.zip › BP.Gsea.1653623667859/enplot_GOBP_EMBRYONIC_PLACENTA_DEVELOPMENT_923.png]

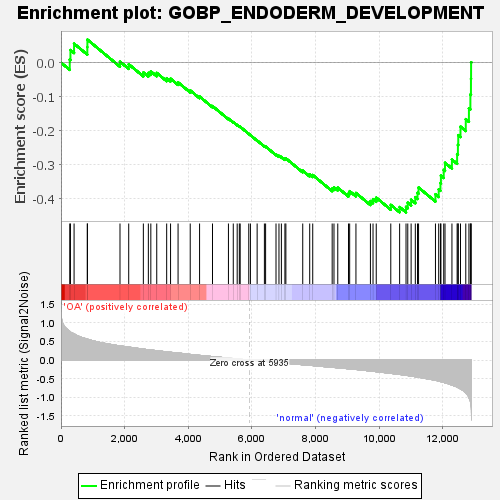

Supplement: Supplementary file 1 — Additional file 1. [file 12891_2023_6585_MOESM1_ESM.zip › BP.Gsea.1653623667859/enplot_GOBP_ENDODERM_DEVELOPMENT_965.png]

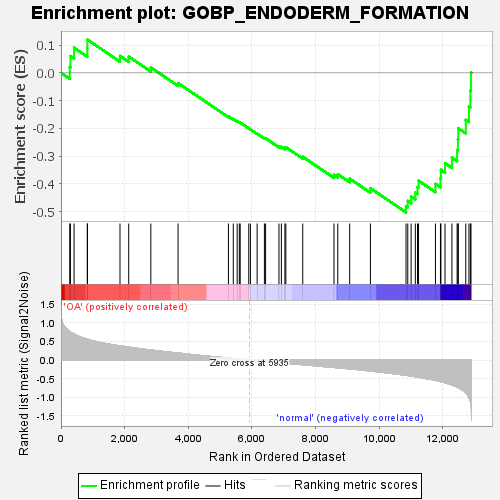

Supplement: Supplementary file 1 — Additional file 1. [file 12891_2023_6585_MOESM1_ESM.zip › BP.Gsea.1653623667859/enplot_GOBP_ENDODERM_FORMATION_887.png]

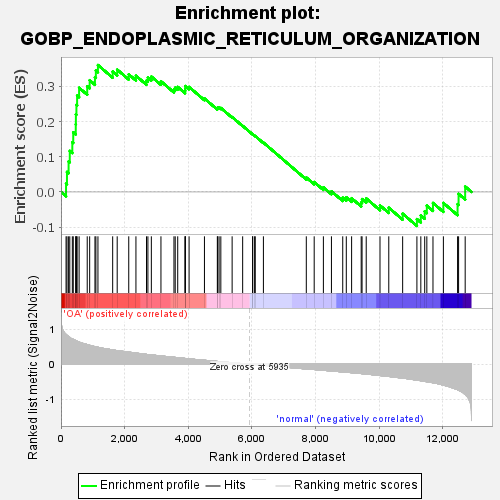

Supplement: Supplementary file 1 — Additional file 1. [file 12891_2023_6585_MOESM1_ESM.zip › BP.Gsea.1653623667859/enplot_GOBP_ENDOPLASMIC_RETICULUM_ORGANIZATION_689.png]

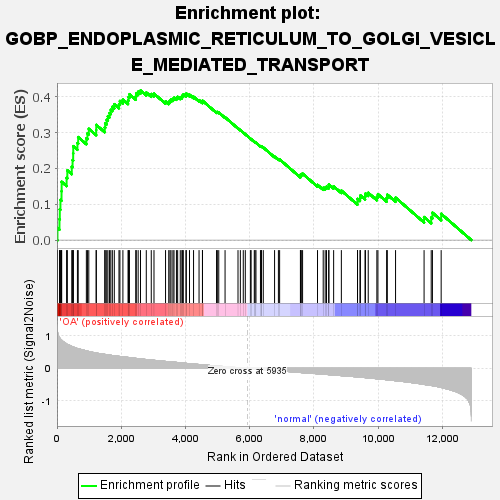

Supplement: Supplementary file 1 — Additional file 1. [file 12891_2023_6585_MOESM1_ESM.zip › BP.Gsea.1653623667859/enplot_GOBP_ENDOPLASMIC_RETICULUM_TO_GOLGI_VESICLE_MEDIATED_TRANSPORT_563.png]

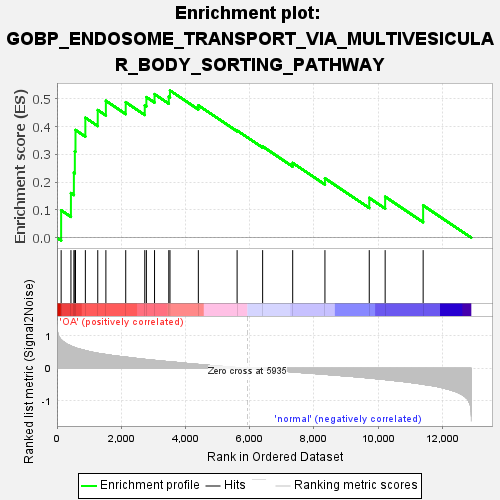

Supplement: Supplementary file 1 — Additional file 1. [file 12891_2023_6585_MOESM1_ESM.zip › BP.Gsea.1653623667859/enplot_GOBP_ENDOSOME_TRANSPORT_VIA_MULTIVESICULAR_BODY_SORTING_PATHWAY_611.png]

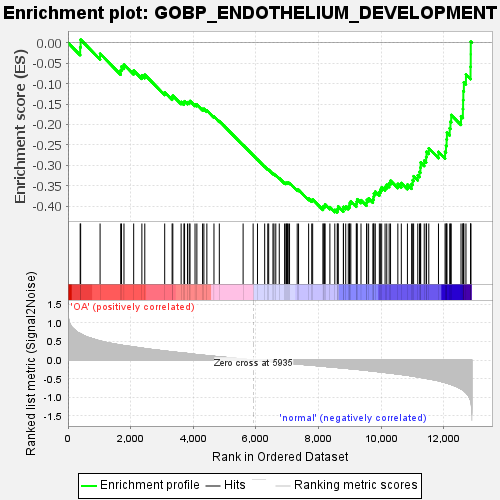

Supplement: Supplementary file 1 — Additional file 1. [file 12891_2023_6585_MOESM1_ESM.zip › BP.Gsea.1653623667859/enplot_GOBP_ENDOTHELIUM_DEVELOPMENT_920.png]

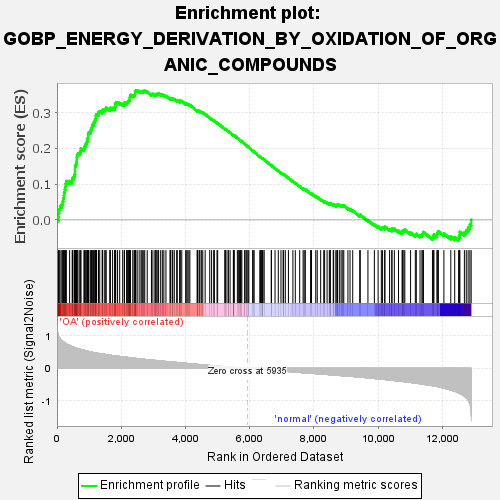

Supplement: Supplementary file 1 — Additional file 1. [file 12891_2023_6585_MOESM1_ESM.zip › BP.Gsea.1653623667859/enplot_GOBP_ENERGY_DERIVATION_BY_OXIDATION_OF_ORGANIC_COMPOUNDS_569.png]

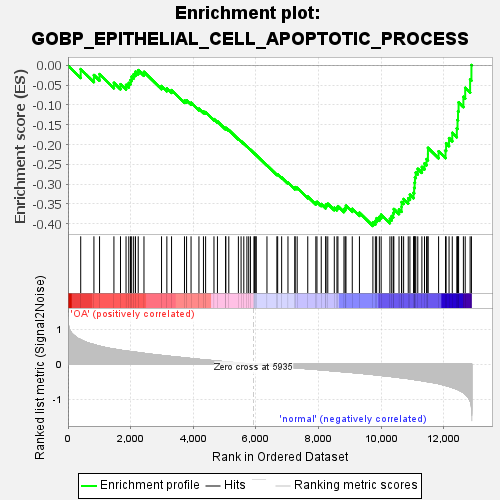

Supplement: Supplementary file 1 — Additional file 1. [file 12891_2023_6585_MOESM1_ESM.zip › BP.Gsea.1653623667859/enplot_GOBP_EPITHELIAL_CELL_APOPTOTIC_PROCESS_971.png]

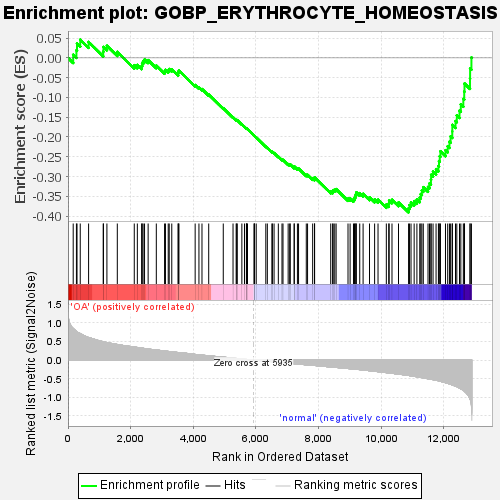

Supplement: Supplementary file 1 — Additional file 1. [file 12891_2023_6585_MOESM1_ESM.zip › BP.Gsea.1653623667859/enplot_GOBP_ERYTHROCYTE_HOMEOSTASIS_1025.png]

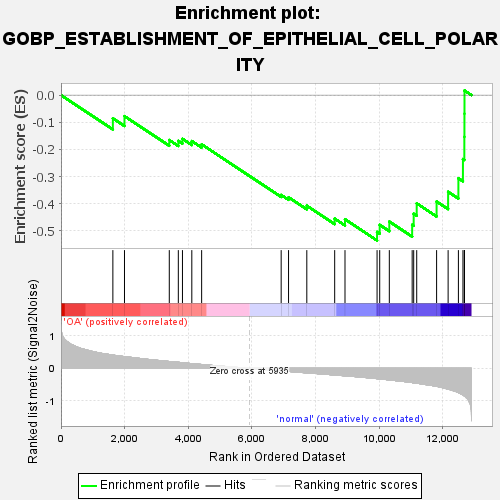

Supplement: Supplementary file 1 — Additional file 1. [file 12891_2023_6585_MOESM1_ESM.zip › BP.Gsea.1653623667859/enplot_GOBP_ESTABLISHMENT_OF_EPITHELIAL_CELL_POLARITY_1064.png]

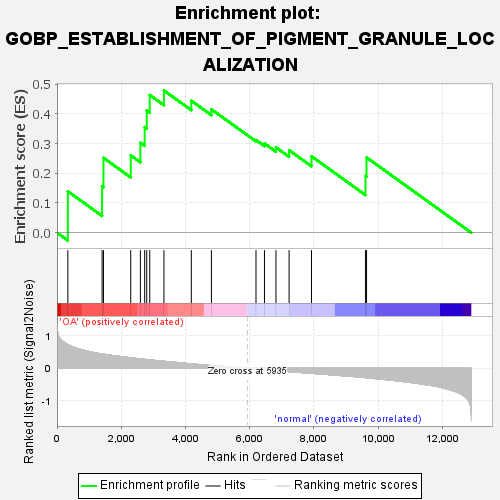

Supplement: Supplementary file 1 — Additional file 1. [file 12891_2023_6585_MOESM1_ESM.zip › BP.Gsea.1653623667859/enplot_GOBP_ESTABLISHMENT_OF_PIGMENT_GRANULE_LOCALIZATION_764.png]

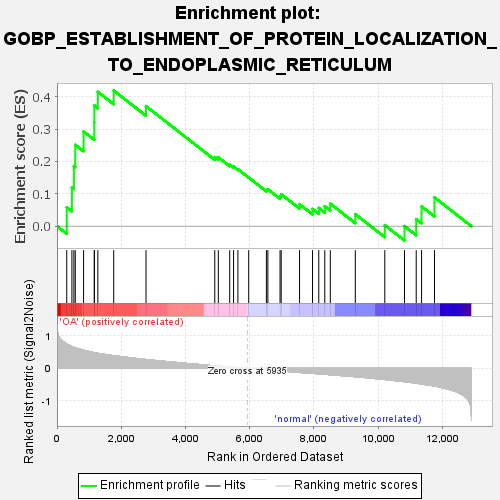

Supplement: Supplementary file 1 — Additional file 1. [file 12891_2023_6585_MOESM1_ESM.zip › BP.Gsea.1653623667859/enplot_GOBP_ESTABLISHMENT_OF_PROTEIN_LOCALIZATION_TO_ENDOPLASMIC_RETICULUM_713.png]

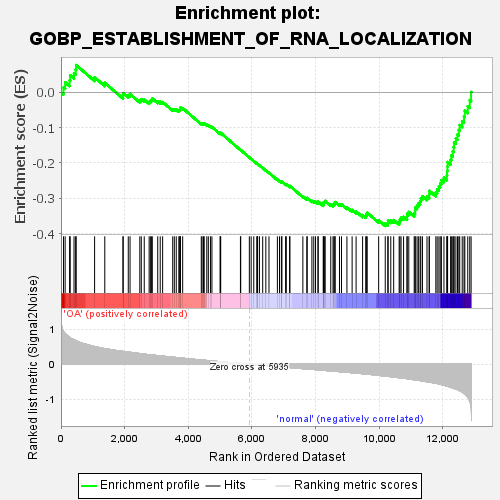

Supplement: Supplementary file 1 — Additional file 1. [file 12891_2023_6585_MOESM1_ESM.zip › BP.Gsea.1653623667859/enplot_GOBP_ESTABLISHMENT_OF_RNA_LOCALIZATION_1037.png]

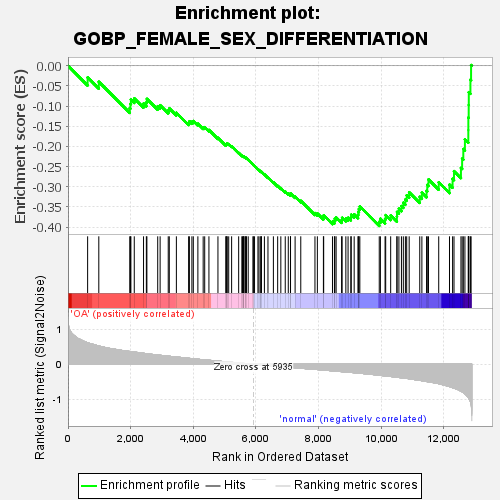

Supplement: Supplementary file 1 — Additional file 1. [file 12891_2023_6585_MOESM1_ESM.zip › BP.Gsea.1653623667859/enplot_GOBP_FEMALE_SEX_DIFFERENTIATION_974.png]

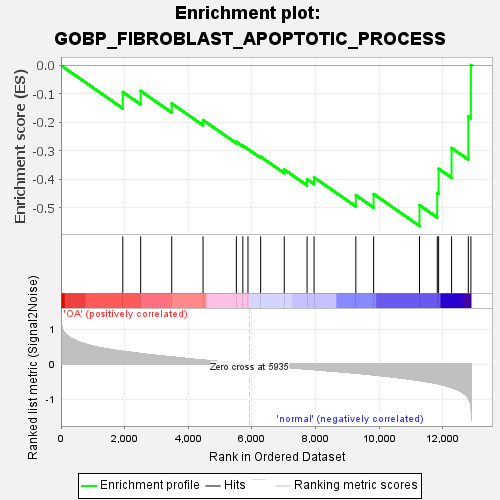

Supplement: Supplementary file 1 — Additional file 1. [file 12891_2023_6585_MOESM1_ESM.zip › BP.Gsea.1653623667859/enplot_GOBP_FIBROBLAST_APOPTOTIC_PROCESS_1082.png]

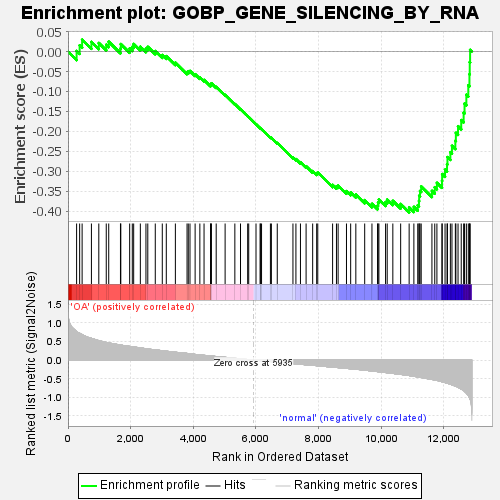

Supplement: Supplementary file 1 — Additional file 1. [file 12891_2023_6585_MOESM1_ESM.zip › BP.Gsea.1653623667859/enplot_GOBP_GENE_SILENCING_BY_RNA_1055.png]

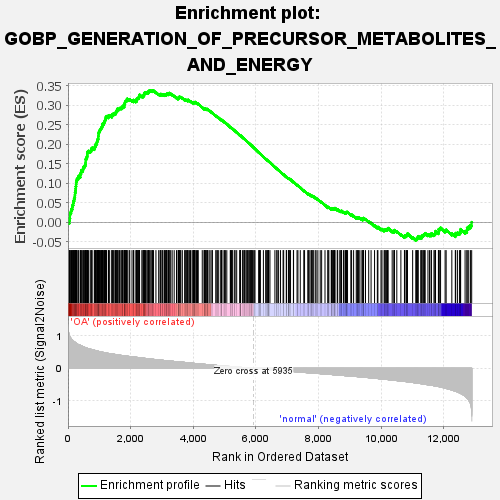

Supplement: Supplementary file 1 — Additional file 1. [file 12891_2023_6585_MOESM1_ESM.zip › BP.Gsea.1653623667859/enplot_GOBP_GENERATION_OF_PRECURSOR_METABOLITES_AND_ENERGY_575.png]

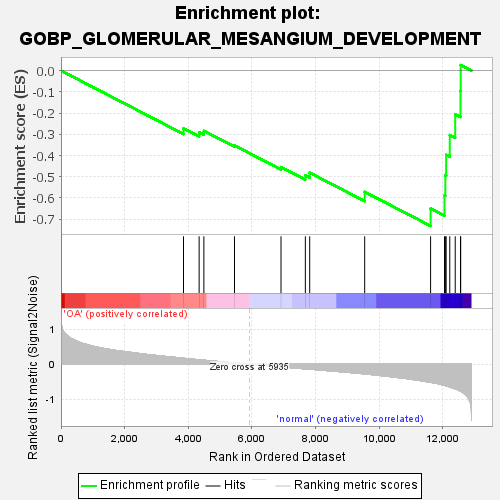

Supplement: Supplementary file 1 — Additional file 1. [file 12891_2023_6585_MOESM1_ESM.zip › BP.Gsea.1653623667859/enplot_GOBP_GLOMERULAR_MESANGIUM_DEVELOPMENT_830.png]

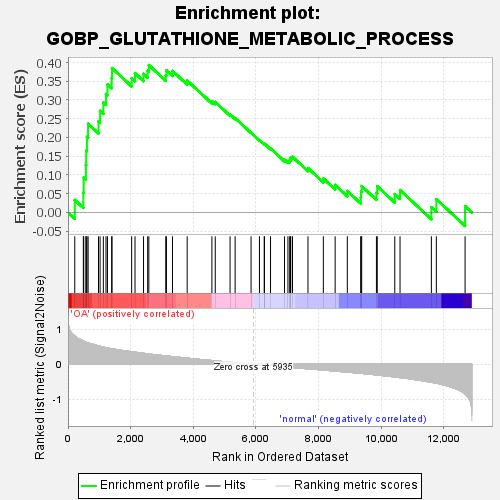

Supplement: Supplementary file 1 — Additional file 1. [file 12891_2023_6585_MOESM1_ESM.zip › BP.Gsea.1653623667859/enplot_GOBP_GLUTATHIONE_METABOLIC_PROCESS_650.png]

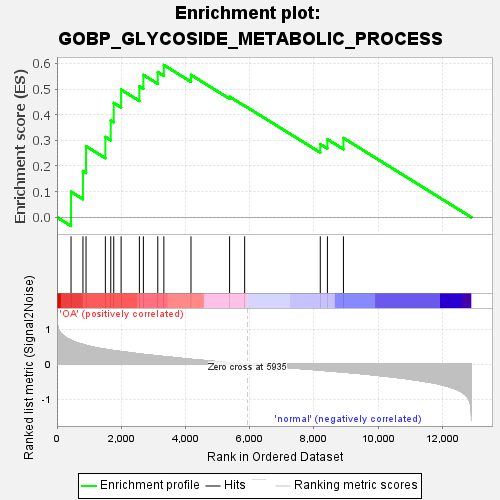

Supplement: Supplementary file 1 — Additional file 1. [file 12891_2023_6585_MOESM1_ESM.zip › BP.Gsea.1653623667859/enplot_GOBP_GLYCOSIDE_METABOLIC_PROCESS_602.png]

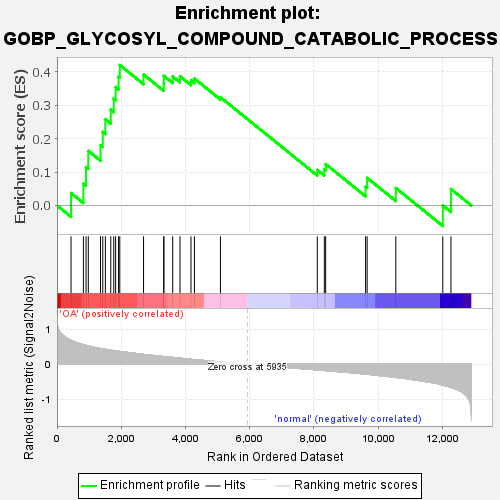

Supplement: Supplementary file 1 — Additional file 1. [file 12891_2023_6585_MOESM1_ESM.zip › BP.Gsea.1653623667859/enplot_GOBP_GLYCOSYL_COMPOUND_CATABOLIC_PROCESS_761.png]

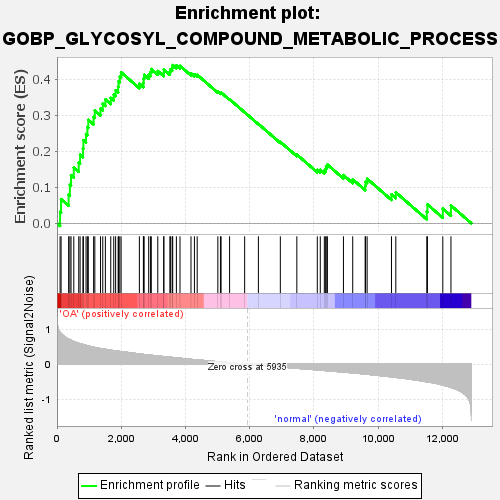

Supplement: Supplementary file 1 — Additional file 1. [file 12891_2023_6585_MOESM1_ESM.zip › BP.Gsea.1653623667859/enplot_GOBP_GLYCOSYL_COMPOUND_METABOLIC_PROCESS_581.png]

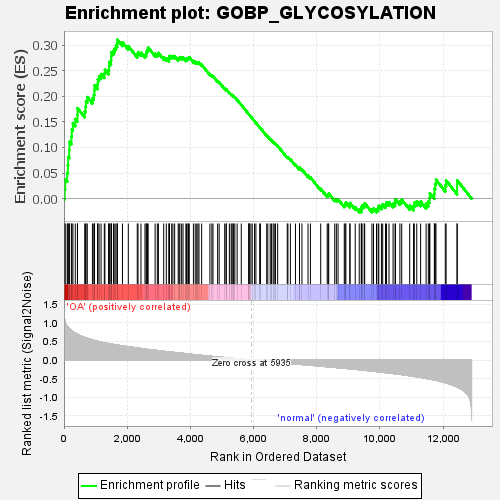

Supplement: Supplementary file 1 — Additional file 1. [file 12891_2023_6585_MOESM1_ESM.zip › BP.Gsea.1653623667859/enplot_GOBP_GLYCOSYLATION_686.png]

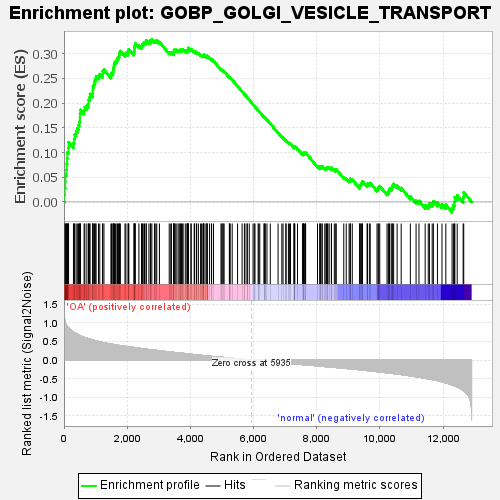

Supplement: Supplementary file 1 — Additional file 1. [file 12891_2023_6585_MOESM1_ESM.zip › BP.Gsea.1653623667859/enplot_GOBP_GOLGI_VESICLE_TRANSPORT_617.png]

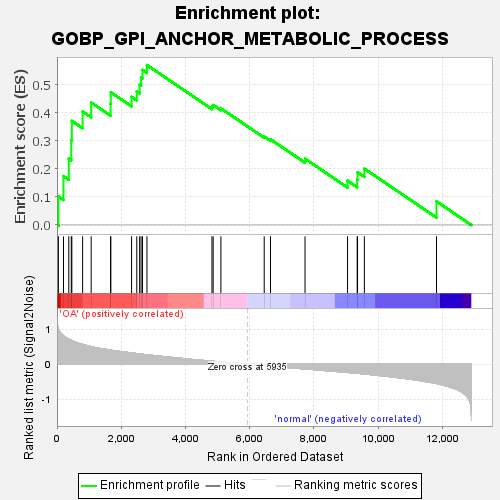

Supplement: Supplementary file 1 — Additional file 1. [file 12891_2023_6585_MOESM1_ESM.zip › BP.Gsea.1653623667859/enplot_GOBP_GPI_ANCHOR_METABOLIC_PROCESS_566.png]

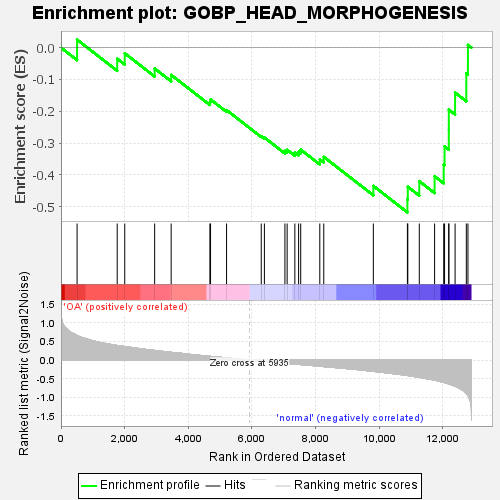

Supplement: Supplementary file 1 — Additional file 1. [file 12891_2023_6585_MOESM1_ESM.zip › BP.Gsea.1653623667859/enplot_GOBP_HEAD_MORPHOGENESIS_1049.png]

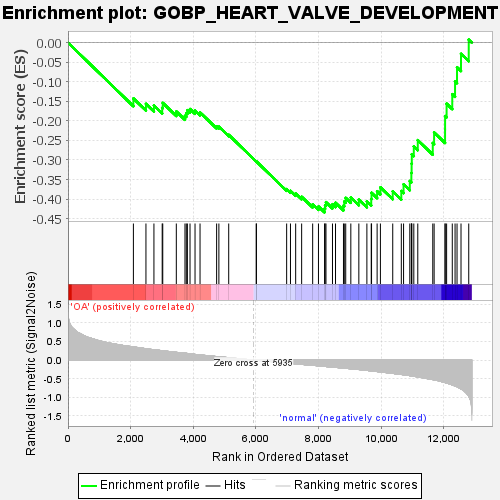

Supplement: Supplementary file 1 — Additional file 1. [file 12891_2023_6585_MOESM1_ESM.zip › BP.Gsea.1653623667859/enplot_GOBP_HEART_VALVE_DEVELOPMENT_1094.png]

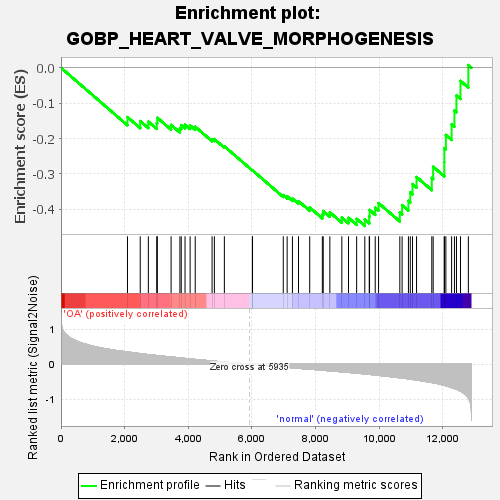

Supplement: Supplementary file 1 — Additional file 1. [file 12891_2023_6585_MOESM1_ESM.zip › BP.Gsea.1653623667859/enplot_GOBP_HEART_VALVE_MORPHOGENESIS_1076.png]

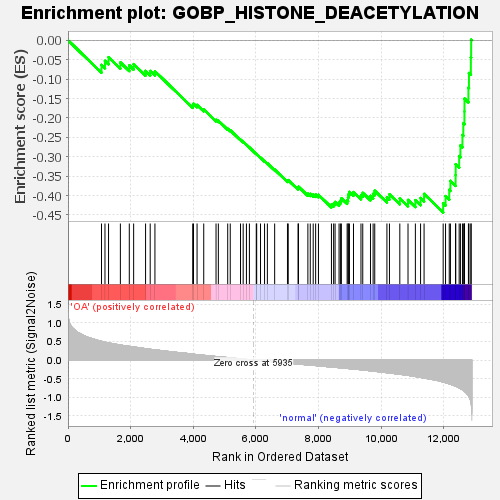

Supplement: Supplementary file 1 — Additional file 1. [file 12891_2023_6585_MOESM1_ESM.zip › BP.Gsea.1653623667859/enplot_GOBP_HISTONE_DEACETYLATION_953.png]

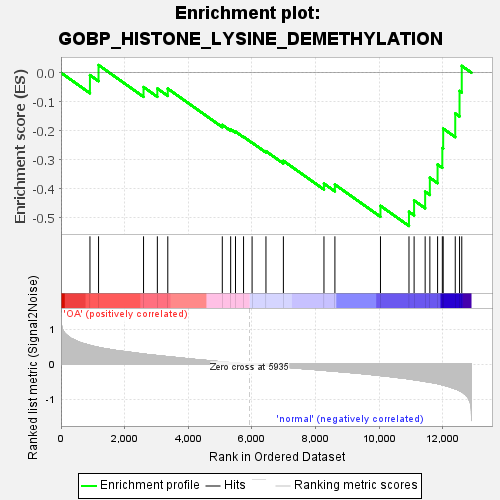

Supplement: Supplementary file 1 — Additional file 1. [file 12891_2023_6585_MOESM1_ESM.zip › BP.Gsea.1653623667859/enplot_GOBP_HISTONE_LYSINE_DEMETHYLATION_1091.png]

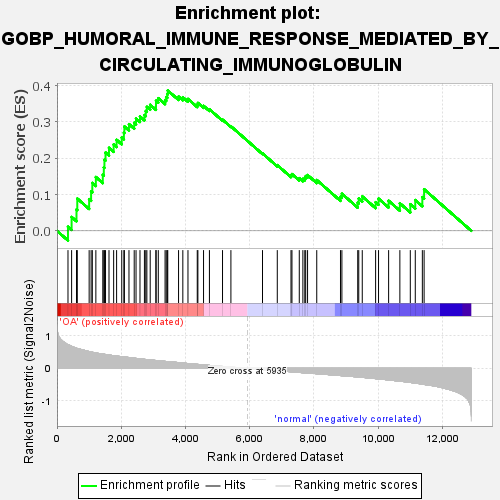

Supplement: Supplementary file 1 — Additional file 1. [file 12891_2023_6585_MOESM1_ESM.zip › BP.Gsea.1653623667859/enplot_GOBP_HUMORAL_IMMUNE_RESPONSE_MEDIATED_BY_CIRCULATING_IMMUNOGLOBULIN_626.png]

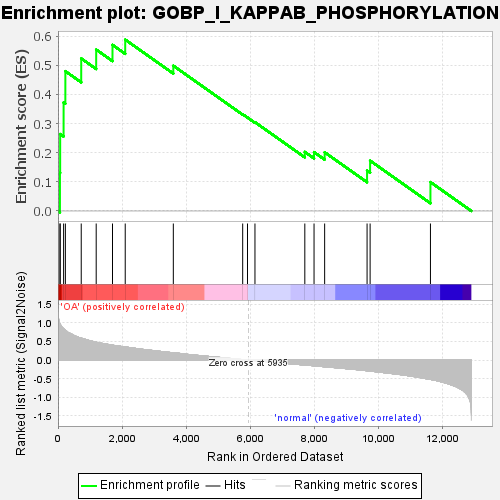

Supplement: Supplementary file 1 — Additional file 1. [file 12891_2023_6585_MOESM1_ESM.zip › BP.Gsea.1653623667859/enplot_GOBP_I_KAPPAB_PHOSPHORYLATION_584.png]

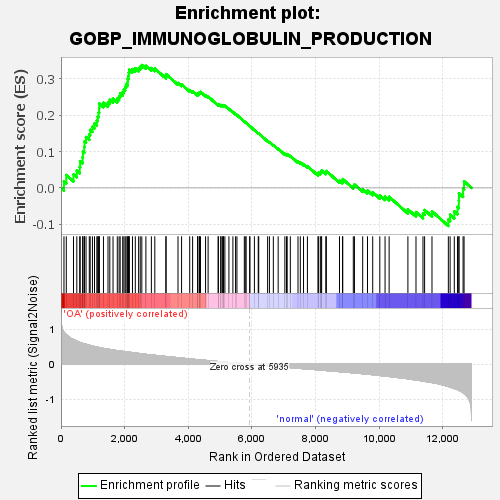

Supplement: Supplementary file 1 — Additional file 1. [file 12891_2023_6585_MOESM1_ESM.zip › BP.Gsea.1653623667859/enplot_GOBP_IMMUNOGLOBULIN_PRODUCTION_662.png]

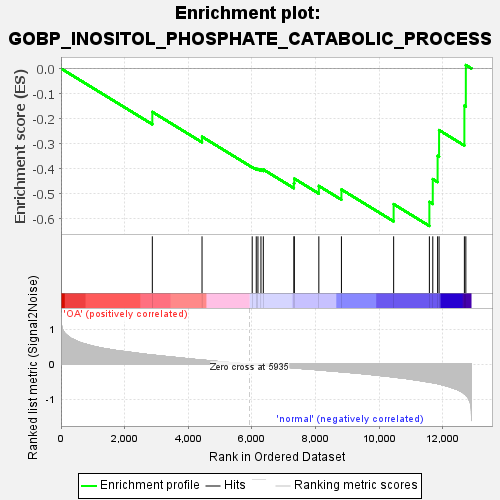

Supplement: Supplementary file 1 — Additional file 1. [file 12891_2023_6585_MOESM1_ESM.zip › BP.Gsea.1653623667859/enplot_GOBP_INOSITOL_PHOSPHATE_CATABOLIC_PROCESS_914.png]

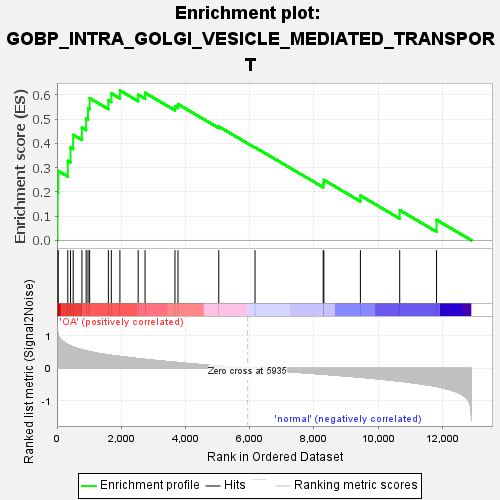

Supplement: Supplementary file 1 — Additional file 1. [file 12891_2023_6585_MOESM1_ESM.zip › BP.Gsea.1653623667859/enplot_GOBP_INTRA_GOLGI_VESICLE_MEDIATED_TRANSPORT_539.png]

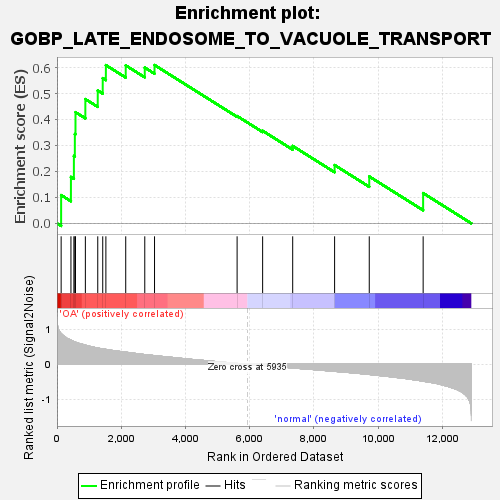

Supplement: Supplementary file 1 — Additional file 1. [file 12891_2023_6585_MOESM1_ESM.zip › BP.Gsea.1653623667859/enplot_GOBP_LATE_ENDOSOME_TO_VACUOLE_TRANSPORT_572.png]

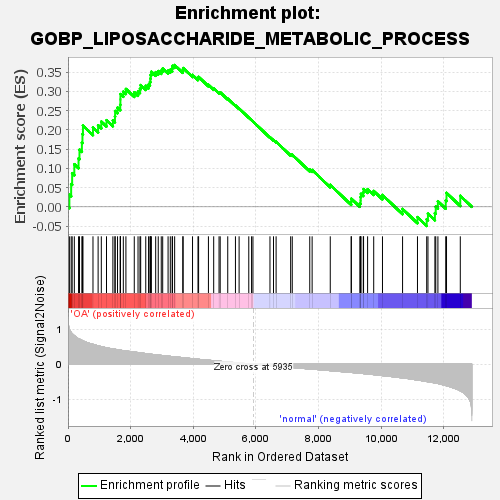

Supplement: Supplementary file 1 — Additional file 1. [file 12891_2023_6585_MOESM1_ESM.zip › BP.Gsea.1653623667859/enplot_GOBP_LIPOSACCHARIDE_METABOLIC_PROCESS_635.png]

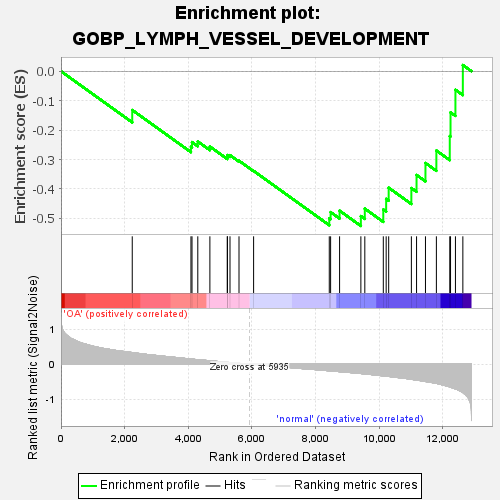

Supplement: Supplementary file 1 — Additional file 1. [file 12891_2023_6585_MOESM1_ESM.zip › BP.Gsea.1653623667859/enplot_GOBP_LYMPH_VESSEL_DEVELOPMENT_1088.png]

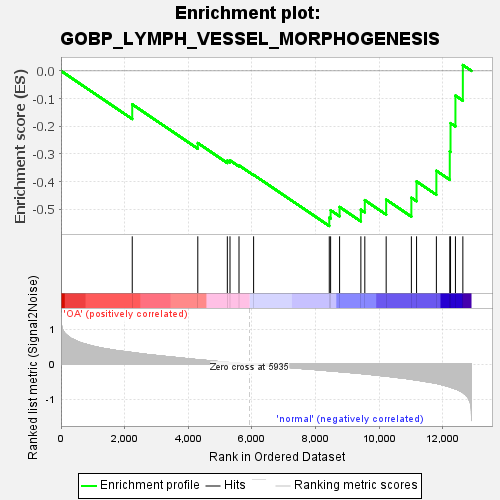

Supplement: Supplementary file 1 — Additional file 1. [file 12891_2023_6585_MOESM1_ESM.zip › BP.Gsea.1653623667859/enplot_GOBP_LYMPH_VESSEL_MORPHOGENESIS_1079.png]

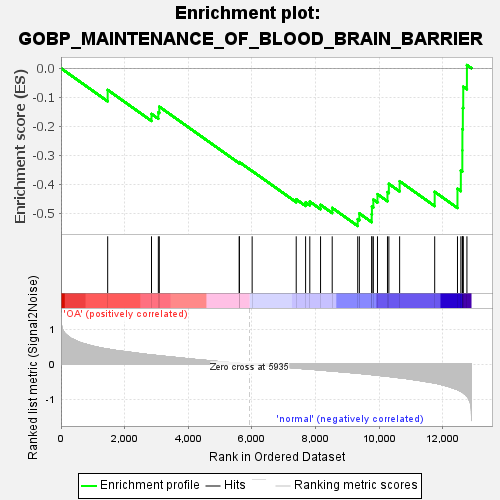

Supplement: Supplementary file 1 — Additional file 1. [file 12891_2023_6585_MOESM1_ESM.zip › BP.Gsea.1653623667859/enplot_GOBP_MAINTENANCE_OF_BLOOD_BRAIN_BARRIER_908.png]

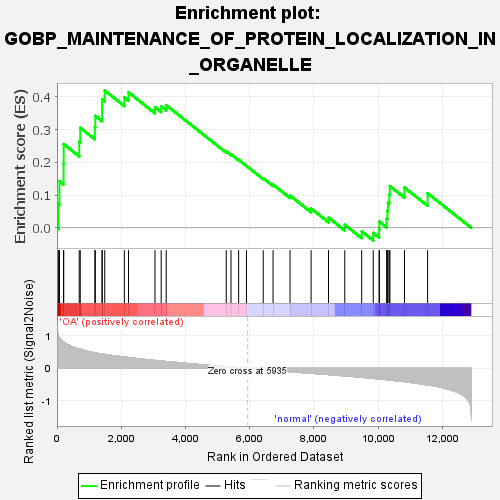

Supplement: Supplementary file 1 — Additional file 1. [file 12891_2023_6585_MOESM1_ESM.zip › BP.Gsea.1653623667859/enplot_GOBP_MAINTENANCE_OF_PROTEIN_LOCALIZATION_IN_ORGANELLE_695.png]

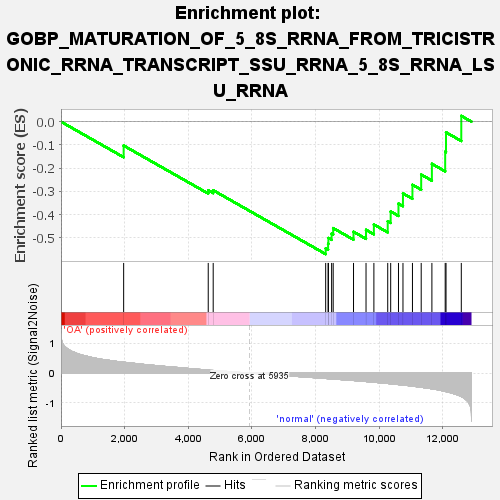

Supplement: Supplementary file 1 — Additional file 1. [file 12891_2023_6585_MOESM1_ESM.zip › BP.Gsea.1653623667859/enplot_GOBP_MATURATION_OF_5_8S_RRNA_FROM_TRICISTRONIC_RRNA_TRANSCRIPT_SSU_RRNA_5_8S_RRNA_LSU_RRNA_1004.png]

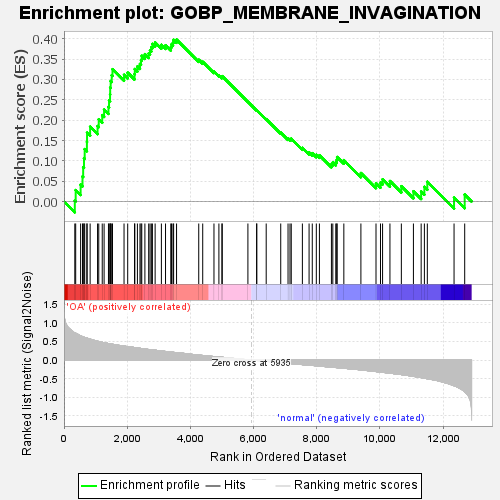

Supplement: Supplementary file 1 — Additional file 1. [file 12891_2023_6585_MOESM1_ESM.zip › BP.Gsea.1653623667859/enplot_GOBP_MEMBRANE_INVAGINATION_614.png]

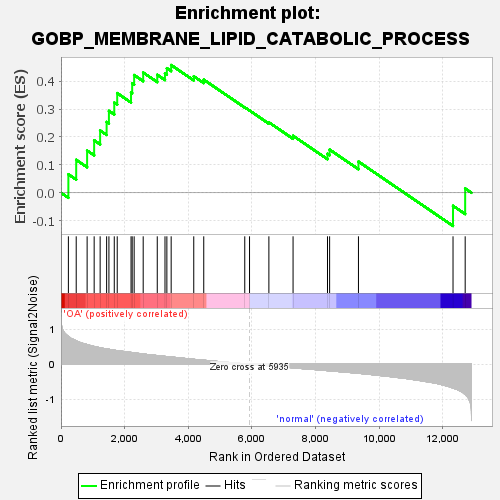

Supplement: Supplementary file 1 — Additional file 1. [file 12891_2023_6585_MOESM1_ESM.zip › BP.Gsea.1653623667859/enplot_GOBP_MEMBRANE_LIPID_CATABOLIC_PROCESS_659.png]

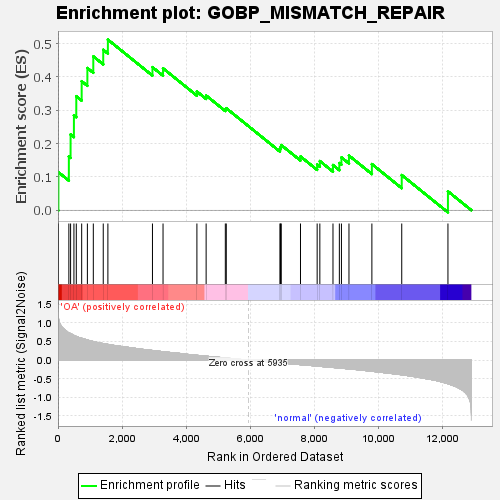

Supplement: Supplementary file 1 — Additional file 1. [file 12891_2023_6585_MOESM1_ESM.zip › BP.Gsea.1653623667859/enplot_GOBP_MISMATCH_REPAIR_593.png]

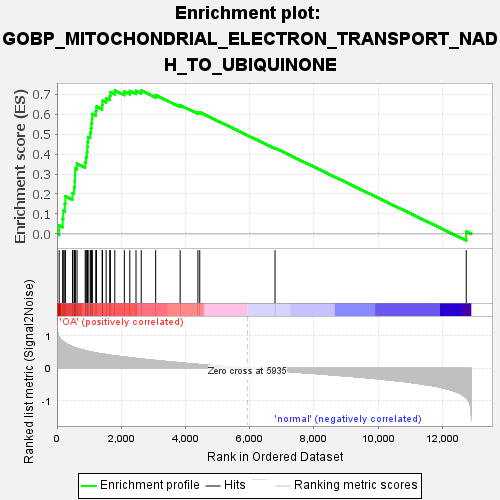

Supplement: Supplementary file 1 — Additional file 1. [file 12891_2023_6585_MOESM1_ESM.zip › BP.Gsea.1653623667859/enplot_GOBP_MITOCHONDRIAL_ELECTRON_TRANSPORT_NADH_TO_UBIQUINONE_515.png]

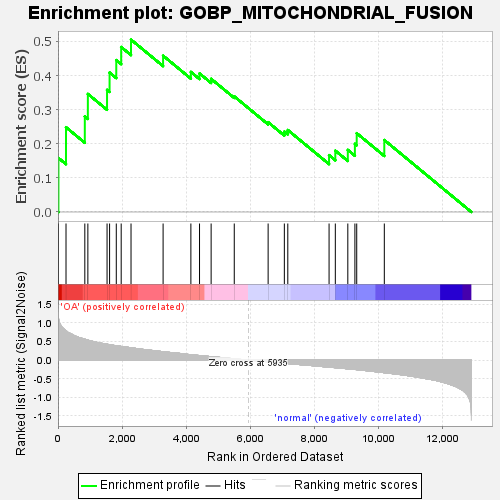

Supplement: Supplementary file 1 — Additional file 1. [file 12891_2023_6585_MOESM1_ESM.zip › BP.Gsea.1653623667859/enplot_GOBP_MITOCHONDRIAL_FUSION_632.png]

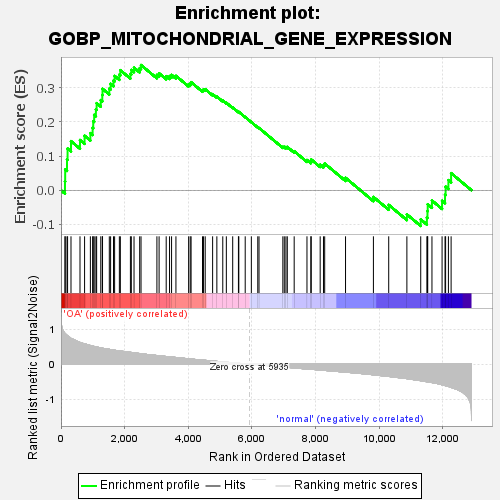

Supplement: Supplementary file 1 — Additional file 1. [file 12891_2023_6585_MOESM1_ESM.zip › BP.Gsea.1653623667859/enplot_GOBP_MITOCHONDRIAL_GENE_EXPRESSION_656.png]

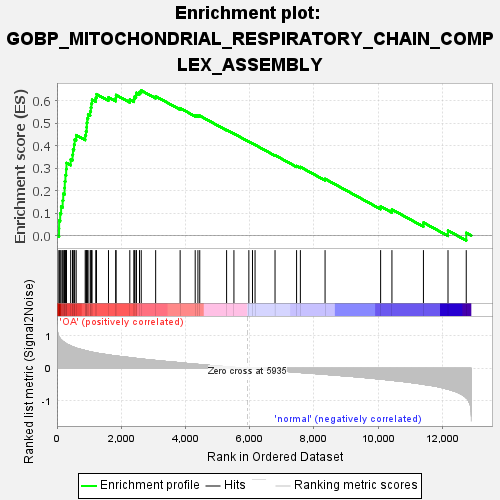

Supplement: Supplementary file 1 — Additional file 1. [file 12891_2023_6585_MOESM1_ESM.zip › BP.Gsea.1653623667859/enplot_GOBP_MITOCHONDRIAL_RESPIRATORY_CHAIN_COMPLEX_ASSEMBLY_521.png]

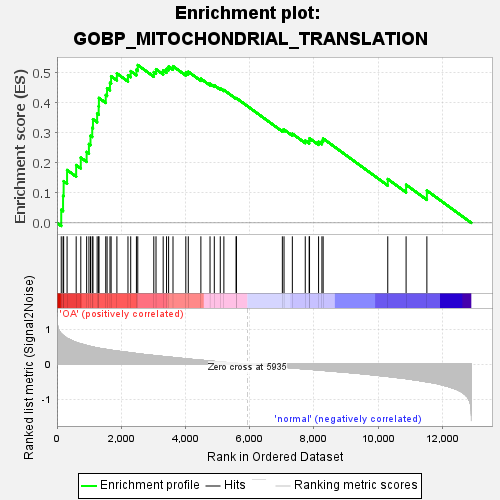

Supplement: Supplementary file 1 — Additional file 1. [file 12891_2023_6585_MOESM1_ESM.zip › BP.Gsea.1653623667859/enplot_GOBP_MITOCHONDRIAL_TRANSLATION_545.png]

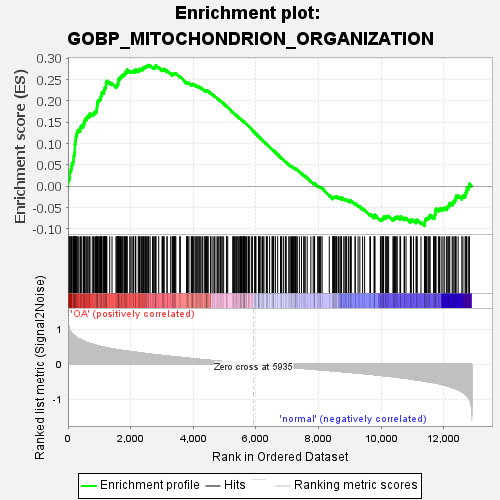

Supplement: Supplementary file 1 — Additional file 1. [file 12891_2023_6585_MOESM1_ESM.zip › BP.Gsea.1653623667859/enplot_GOBP_MITOCHONDRION_ORGANIZATION_671.png]

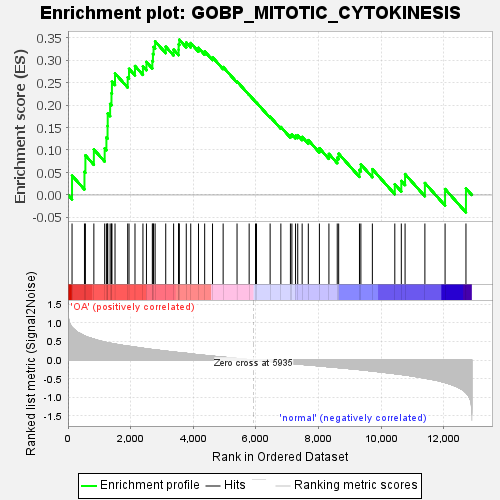

Supplement: Supplementary file 1 — Additional file 1. [file 12891_2023_6585_MOESM1_ESM.zip › BP.Gsea.1653623667859/enplot_GOBP_MITOTIC_CYTOKINESIS_773.png]

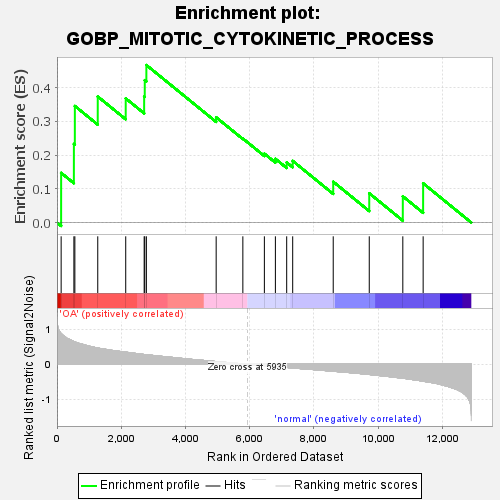

Supplement: Supplementary file 1 — Additional file 1. [file 12891_2023_6585_MOESM1_ESM.zip › BP.Gsea.1653623667859/enplot_GOBP_MITOTIC_CYTOKINETIC_PROCESS_767.png]

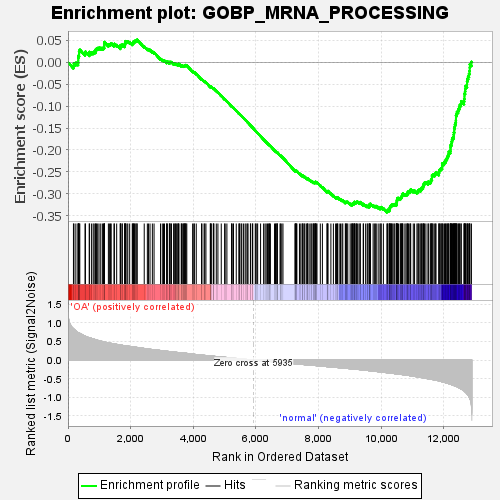

Supplement: Supplementary file 1 — Additional file 1. [file 12891_2023_6585_MOESM1_ESM.zip › BP.Gsea.1653623667859/enplot_GOBP_MRNA_PROCESSING_1007.png]

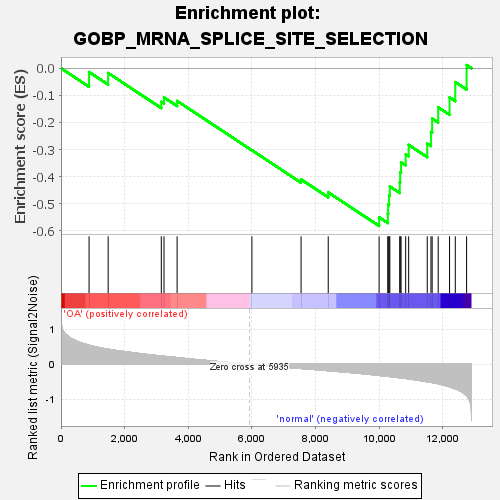

Supplement: Supplementary file 1 — Additional file 1. [file 12891_2023_6585_MOESM1_ESM.zip › BP.Gsea.1653623667859/enplot_GOBP_MRNA_SPLICE_SITE_SELECTION_884.png]

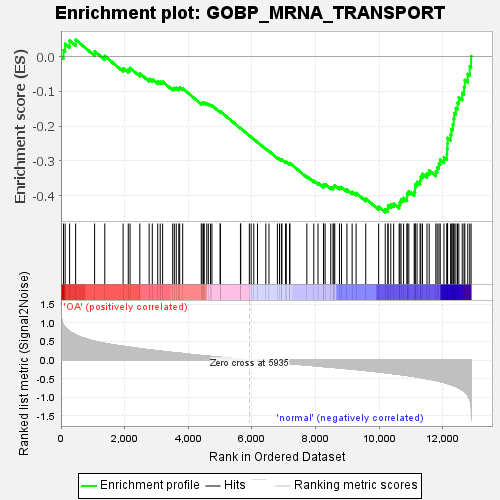

Supplement: Supplementary file 1 — Additional file 1. [file 12891_2023_6585_MOESM1_ESM.zip › BP.Gsea.1653623667859/enplot_GOBP_MRNA_TRANSPORT_854.png]

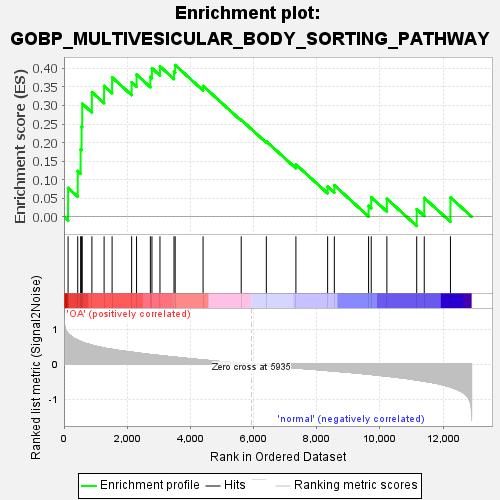

Supplement: Supplementary file 1 — Additional file 1. [file 12891_2023_6585_MOESM1_ESM.zip › BP.Gsea.1653623667859/enplot_GOBP_MULTIVESICULAR_BODY_SORTING_PATHWAY_788.png]

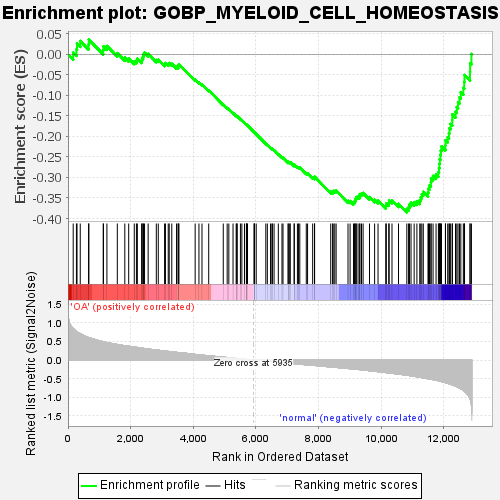

Supplement: Supplementary file 1 — Additional file 1. [file 12891_2023_6585_MOESM1_ESM.zip › BP.Gsea.1653623667859/enplot_GOBP_MYELOID_CELL_HOMEOSTASIS_983.png]

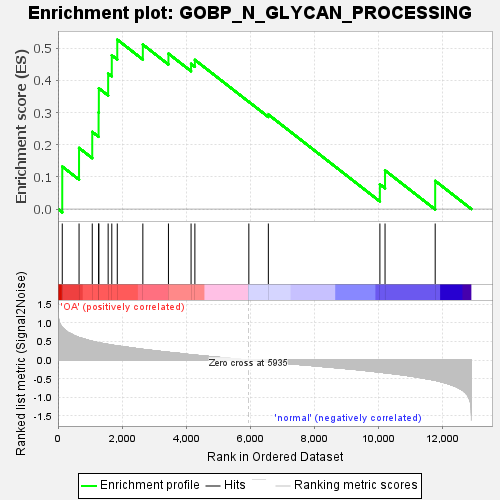

Supplement: Supplementary file 1 — Additional file 1. [file 12891_2023_6585_MOESM1_ESM.zip › BP.Gsea.1653623667859/enplot_GOBP_N_GLYCAN_PROCESSING_653.png]

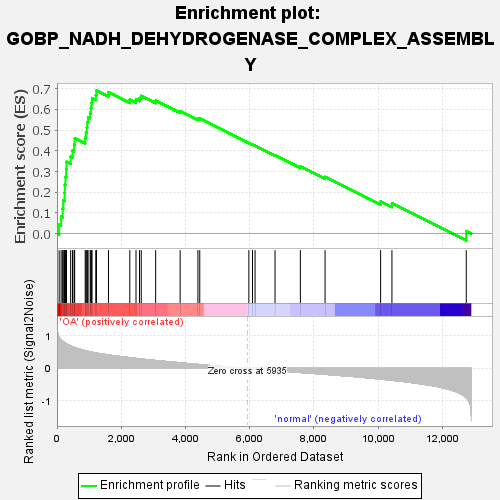

Supplement: Supplementary file 1 — Additional file 1. [file 12891_2023_6585_MOESM1_ESM.zip › BP.Gsea.1653623667859/enplot_GOBP_NADH_DEHYDROGENASE_COMPLEX_ASSEMBLY_524.png]

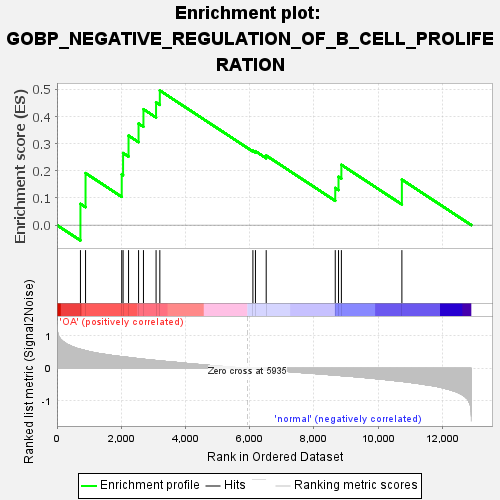

Supplement: Supplementary file 1 — Additional file 1. [file 12891_2023_6585_MOESM1_ESM.zip › BP.Gsea.1653623667859/enplot_GOBP_NEGATIVE_REGULATION_OF_B_CELL_PROLIFERATION_743.png]

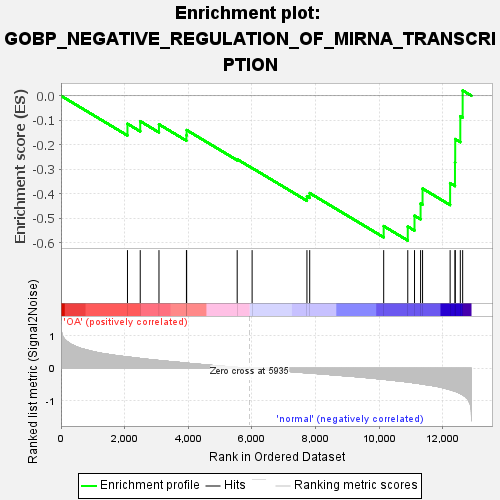

Supplement: Supplementary file 1 — Additional file 1. [file 12891_2023_6585_MOESM1_ESM.zip › BP.Gsea.1653623667859/enplot_GOBP_NEGATIVE_REGULATION_OF_MIRNA_TRANSCRIPTION_962.png]

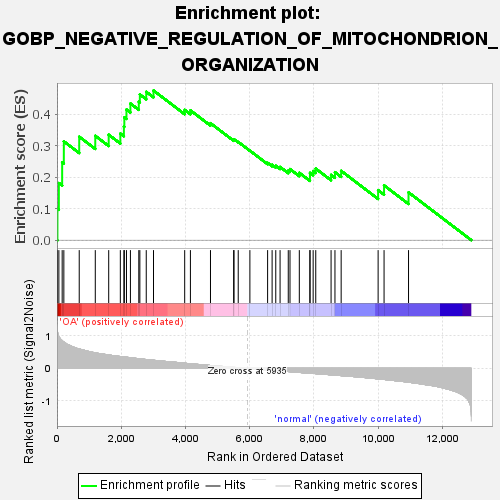

Supplement: Supplementary file 1 — Additional file 1. [file 12891_2023_6585_MOESM1_ESM.zip › BP.Gsea.1653623667859/enplot_GOBP_NEGATIVE_REGULATION_OF_MITOCHONDRION_ORGANIZATION_587.png]

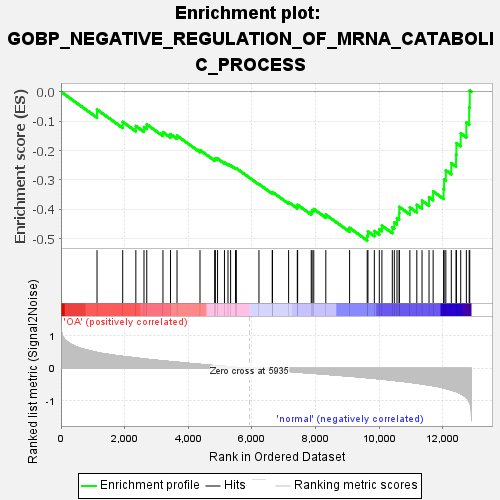

Supplement: Supplementary file 1 — Additional file 1. [file 12891_2023_6585_MOESM1_ESM.zip › BP.Gsea.1653623667859/enplot_GOBP_NEGATIVE_REGULATION_OF_MRNA_CATABOLIC_PROCESS_869.png]
